# SubQ-Sim: A Subcutaneous Physiologically Based Biopharmaceutics Model. Part 1: The Injection and System Parameters

**DOI:** 10.1007/s11095-023-03567-0

**Published:** 2023-08-27

**Authors:** Xavier J. H. Pepin, Iain Grant, J. Matthew Wood

**Affiliations:** 1 Regulatory Affairs, Simulations Plus, Lancaster, CA USA; 2 Innovation Strategy & External Liaison, Pharmaceutical Technology & Development, Operations, AstraZeneca, Charter Way, Macclesfield, SK10 2NA UK; 3 New Modalities and Parenteral Development, Pharmaceutical Technology & Development, Operations, AstraZeneca, Macclesfield, UK

**Keywords:** back-flow, backpressure, depot, injection, modeling and simulation, PBBM, physiology, subcutaneous

## Abstract

**Purpose:**

To construct a detailed mechanistic and physiologically based biopharmaceutics model capable of predicting 1) device-formulation-tissue interaction during the injection process and 2) binding, degradation, local distribution, diffusion, and drug absorption, following subcutaneous injection. This paper is part of a series and focusses on the first aspect.

**Methods:**

A mathematical model, SubQ-Sim, was developed incorporating the details of the various substructures within the subcutaneous environment together with the calculation of dynamic drug disposition towards the lymph ducts and venous capillaries. Literature was searched to derive key model parameters in healthy and diseased subjects. External factors such as body temperature, exercise, body position, food or stress provide a means to calculate the impact of “life events” on the pharmacokinetics of subcutaneously administered drugs.

**Results:**

The model predicts the tissue backpressure time profile during the injection as a function of injection rate, volume injected, solution viscosity, and interstitial fluid viscosity. The shape of the depot and the concentrations of the formulation and proteins in the depot are described. The model enables prediction of formulation backflow following premature needle removal and the resulting formulation losses. Finally, the effect of disease (type 2 diabetes) or the presence of recombinant human hyaluronidase in the formulation on the injection pressure, are explored.

**Conclusions:**

This novel model can successfully predict tissue back pressure, depot dimensions, drug and protein concentration and formulation losses due to incorrect injection, which are all important starting conditions for predicting drug absorption from a subcutaneous dose. The next article will describe the absorption model and validation against clinical data.

**Supplementary Information:**

The online version contains supplementary material available at 10.1007/s11095-023-03567-0.

## Introduction

The subcutaneous (SC) route is an important administration route for new molecular entities such as peptides, antisense oligonucleotides or antibody drug conjugates, including long-acting formulations designed to release the drug over months to improve patient compliance. With adequate formulation strategies, the absorption rate in the systemic circulation can be manipulated to prolong the duration of pharmacological action and reduce the administration frequency of selected drugs. The SC route is compatible with more types of excipients and formulations than the intravenous or other parenteral routes and is well suited with patient self-administration. To achieve long-term oncology or HIV treatments, subcutaneous administration is increasingly utilized [1–3]. The absorption pathways for drugs from the SC space or adipose tissue (AT) have been described in the literature and some models have been proposed to predict the absorption rate from the subcutaneous space [4–6]. However, the impact of physiology, life events, administration site and disease have not been extensively covered. With this series of articles, the authors propose a novel physiologically based biopharmaceutics model named SubQ-Sim, which will address the mechanisms of drug absorption from the SC space, allow a description of the system parameters to a level of detail sufficient to predict within and between subject variability and elucidate the impact of external factors which may impact drug absorption. SubQ-Sim v2.0 focusses on solutions and mostly non-small molecules, but future versions will comprise several types of formulations, including prolonged release suspensions.

This series will start with a first article describing the overall model structure, main system parameters and the injection model. This first article will also detail the sources and the data utilized to parameterize individual physiological models. The second article will cover the drug absorption processes and related physiological and biopharmaceutical phenomena, together with the model validation against clinical data. The articles will present in the body text the main model assumptions and results and will also provide supplementary materials with the detailed equations and input parameters. The aim of this series of articles is to provide the scientific community (across academics, pharmaceutical industry, software developers and regulatory bodies) with new mechanistic insights to build upon and refine, hopefully catalyzing an increased level of cross-discipline collaboration. The objective of SubQ-Sim is that the number of unnecessary animal and human evaluations needed to develop subcutaneous formulations and their associated devices, can be reduced to the strict minimum.

## Model Structure and System Parameters

**Fig. 1:**
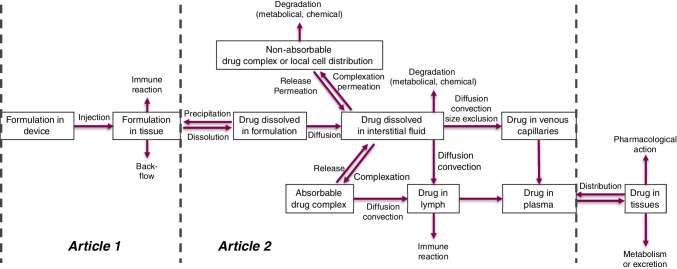
Main model processes in SubQ-Sim v2.0.

The model structure and main processes involved in the administration, release and absorption of drugs from the SC space are schematized in Fig. 1.

Upon administration of the drug product, the injection parameters and properties of the formulation will define the shape and size of the depot that is formed in the SC space, as well as the potential losses of formulation through the puncture hole (formulation backflow). As soon as injection commences, the drug can be released from the depot, associate with the constituents of the subcutaneous space and diffuse towards the capillaries or the lymph ducts. There are potential drug losses during the absorption process, through irreversible binding, chemical or metabolic degradation, which result from the environmental conditions of the formulation, the presence of mobile or transmembrane enzymes in the tissue to which the drug is a substrate, or to the capture of drug aggregates by the lymphoid tissue. Both the lymphatic and venous capillary absorption pathways lead to systemic availability, although lymphatic drainage is expected to induce a delay due to the time spent in the lymphatic network prior to reaching the aorta through the thoracic duct. This first article will focus on the injection depot formation and associated parameters, and provide an overview of the system parameters for the model.

**Fig. 2:**
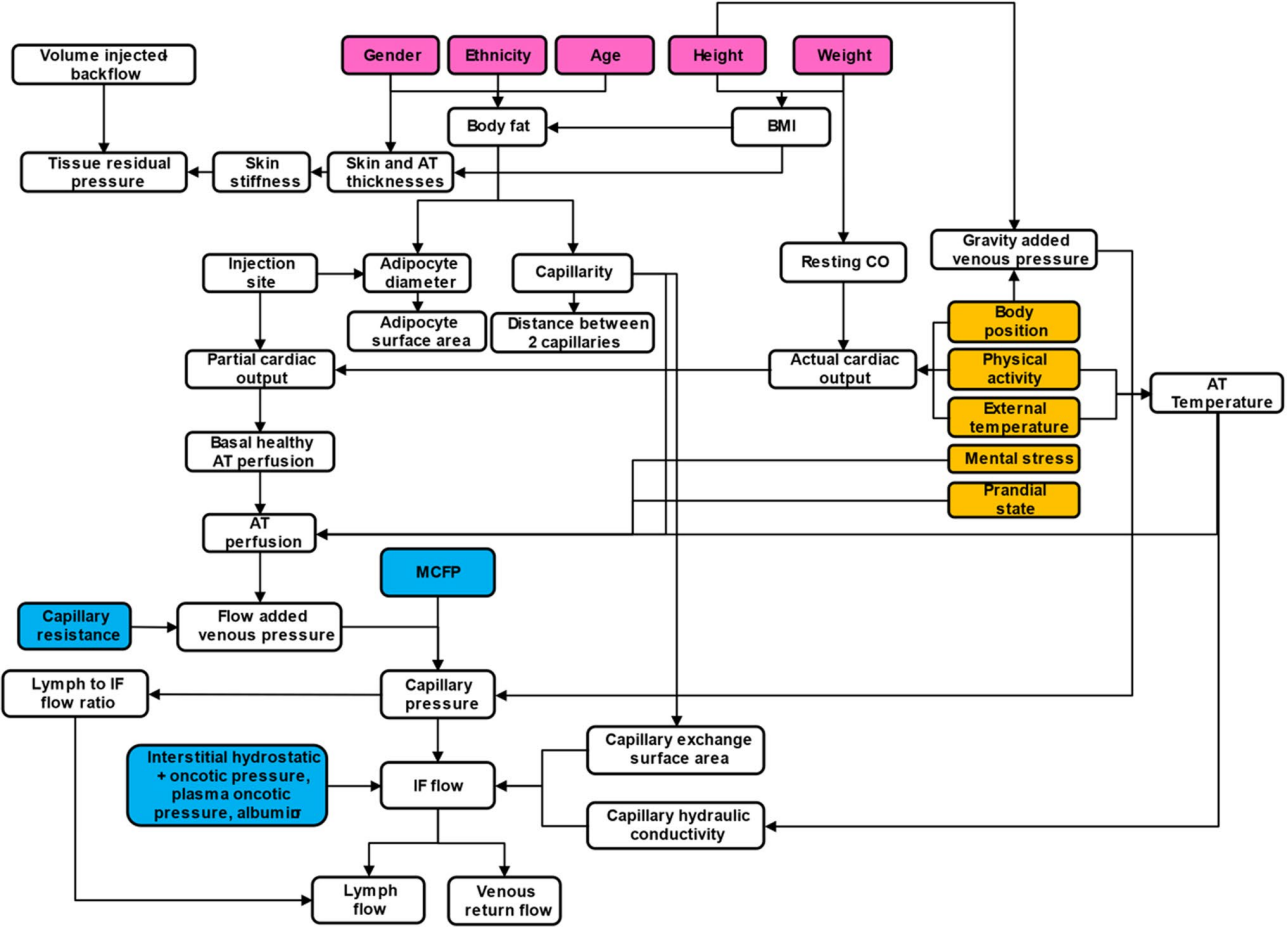
Main system parameters in SubQ-Sim 2.0 and how they are linked. Notes: AT: Adipose tissue, BMI: Body mass index, CO: Cardiac output, IF: Interstitial fluid, MCFP: Mean circulatory filling pressure, σ: capillary pore reflection coefficient. The pink background parameters are the subject covariates, the yellow background parameters are the main “life events”. Blue background parameters are physiological constants. All other parameters are variable.

To derive an adaptable and personalized model, all the absorption processes were described based on first principles and the system parameters were defined based on reported values in the literature. Special care was taken to link the system parameters to patient covariates to enable individualized simulations. The relationship between the system parameters within SubQ-Sim v2.0 are shown schematically in Fig. 2 for humans. These system parameters focus on the inputs needed to describe the dynamic fluid flow and protein concentrations in the AT as well as the capillarity, size of the cells as a function of health or disease. The main system parameters are subsequently used to derive the values needed to model most of the processes described in Fig. 1. Once the drug is absorbed, a classical compartmental model is attached to the SC space, although in the future, the SC space could be easily integrated to a full PBPK model.

In addition to the impact of subject co-variates on the values for the adipose tissue system parameters, the model can also be modulated by “life events”. These correspond to the external factors that the subject is exposed to, that are known to influence the physiology of the subcutaneous space. For example, parameters such as the intake of food, its calorific value and timing, the temperature external to the body, the level of mental stress, the body position, and the amount of physical exercise are called “life events”. These parameters for example all impact the adipose tissue blood flow. The presence of disease such as diabetes or obesity may modulate this response due to the changes in the number and properties of the capillaries in the subcutaneous space. Other factors such as the choice of injection site, needle size, injection angle, injection rate and hold time at the end of injection prior to needle removal will be described in this article as potential human factors which control the size and shape of the depot, together with the amount of formulation and subsequently drug dose which can be lost by the formulation backflow through the puncture hole. Overall, the system parameters in SubQ-Sim are inter-linked, and provide for an individualized approach for both basal system parameter calculation in health and disease, but also for evaluating the impact of inter-occasion variability. This variability can be related to how the injection itself is performed, or how the patient life events before or during the drug absorption phase influence the SC physiology and therefore the rate of drug absorption. A full description of SubQ-Sim system parameters, and how they are linked, is provided in the supplementary materials.

## The Adipose Tissue

A detailed description of the AT is not the purpose of this article, but a summary is needed to support the understanding of how certain model parameters are derived for the injection simulation. The AT is situated approximately 1.1 mm below the skin, after the epidermis and dermis. The actual thickness of AT will vary on the anatomical region and on the body mass index (BMI) (See supplementary materials). The AT comprises arteries and veins joined by a capillary bed, tissue cells and lymphatic capillaries. The tissue cells comprise mature adipocytes embedded in the stroma with preadipocytes, fibroblasts, immune cells, and endothelial cells. The interstitial space comprises fluid and insoluble proteo-glycans which hold the tissue together and provide its elasticity and resistance to tear.

**Fig. 3:**
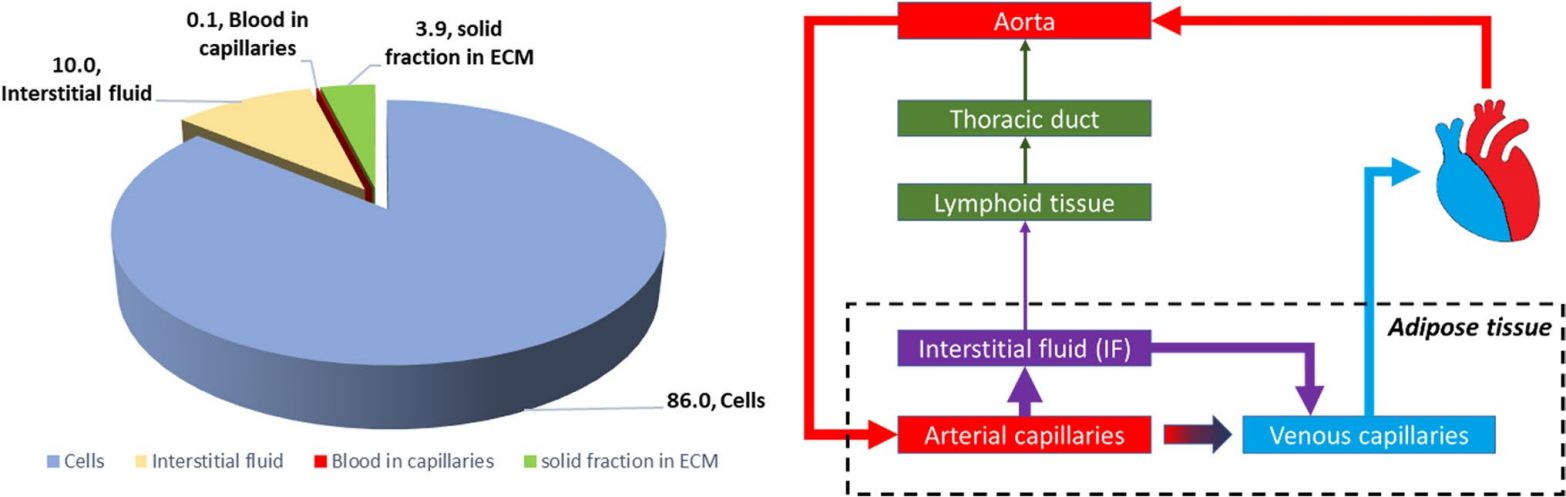
Percent volume distribution in the adipose tissue (left panel) and liquid fluxes through the adipose tissue (right panel).

The volumes in the adipose tissue can be expressed as follows: The tissue density is *D*_adipose_ = 0.92 g/mL [7, 8], the total adipose tissue mass (*M*_adipose_) is 12.5 kg and 17.5 kg in adult males and females respectively [9], and the partial cardiac output (*PCO*_adipose_) to the adipose tissue is 5% to 8.5% for adult males and females respectively. Fractional cell volumes and extracellular volumes are reported in the physiologically based PBPK literature and for adipose amount to 0.86 and 0.14 respectively [10]. The fractional blood volume *θ*_Blood_ comprised in the capillaries of the tissue can be given by the following equation:

1 $${\theta}_{Blood}=\frac{\ {V}_{Blood}\times {D}_{adipose}}{M_{adipose}}\times \frac{\kern0.50em {PBlood}_{cap}}{100}\times \frac{\ {PCO}_{adipose}}{100}$$

where *V*_*Blood*_ is the volume of blood in the body, *PBlood*_*cap*_ is the percentage of blood in the capillaries which is on average 6% [11]. The fractional blood volume of the adipose tissue is therefore around 0.00115 [0.00087–0.00143]. The interstitial fluid (IF) fractional volume is estimated at 0.10. The remaining volume can be estimated as the extracellular solid volume fraction. The overall picture for adipose tissue volume distribution is given in Fig. 3.

Due to their volume and vicinity with the SC depot, adipocytes may serve as a reservoir for the drug depending on its affinity with the cell’s constituents or surface and how fast the drug can permeate the cell membranes. The cell membranes themselves can serve to adsorb drug. Macrophages will also be found in the interstitial space, as they infiltrate the depot and eliminate particulate matter from the administration site. The extracellular matrix (ECM) is composed of an immobile fibrous collagen network, and glycosaminoglycans (GAGs) which form a gel phase. There are four main classes of GAGs: heparin/heparan sulfate, chondroitin/dermatan sulfate, keratin sulfate, and hyaluronan. Only hyaluronan is free and unconjugated in the ECM and the other GAGs are attached to proteins to form proteoglycans which are either membrane bound or free in the ECM. Interstitial fibres carry many fixed negative charges. Collagen itself carries very little charge at physiological pH. The GAGs carry one (hyaluronate, keratan sulphate) to two (chondroitin sulphate) fixed negative charges per disaccharide unit (450–513 Da) at physiological pH. All the solutes, fluids or white cells diffuse through the ECM and the diffusion coefficients of these moieties will need to account for the steric hindrance due to the fibres in the matrix. Similar to what is seen in other physiological gel diffusions, the charge of the diffusing species will also influence the apparent diffusion coefficient [12]. This fibrillar matrix may also serve as a binding site for drugs and the constituents of this matrix serve as building blocks for the fibrous capsule which may form around the injected depots over time [13]. In addition, it provides an “exoskeleton” to adipocytes and allows them to resist to large variations of external mechanical pressure or internal pressure due to the storage of fat vesicles. Proteoglycans are a specific subset of glycoproteins found at the cell surface and in the extracellular matrix, where they interact with a plethora of proteins involved in metabolic homeostasis and meta-inflammation. They can be linked to the cell surfaces or secreted and found in the ECM. For a thorough review on proteoglycans the reader is directed to Pessentheiner *et al.* [14].

### Fluid Fluxes in the Adipose Tissue

A schematic diagram of the liquid fluxes in the adipose tissue is proposed in Fig. 3. The interstitial fluid (IF) is ultra-filtrated from the plasma through the pores of the arterial capillaries and in normal healthy adults, roughly 20 L IF are produced out of the 8400 L blood pumped every day by the heart. On the average the IF to blood filtration ratio *R*_IF/B_ can be given by the ratio of these fluxes, i.e., in normal conditions of around 0.0024. Out of the 20 L IF produced every day, 3 L are returned to the systematic circulation through the lymphatic system and the rest enters the venous capillaries (Fig. 3). The values for cardiac output (CO) of a 73 kg supine adult male is of 6700 mL/min and that for a 60 kg supine adult female of 5800 mL/min [9]. When standing or sitting, these blood flow values are typically increased by 20%. Blood volumes in a male and a female amount to *V*_Blood_ = 5.21 L and 4.29 L respectively and the hematocrit Ht = 0.47 and 0.42 respectively [15]. Partial cardiac outputs to the adipose tissues are reported to have regional differences: from 3.7% in the leg, 5% in the feet and 11.8% in the abdomen in males [15]. A range in males can be given from 3.7–11.8% and in females from 6.3–20.1%. Following these considerations, the adipose tissue blood perfusion rates can be given by *PR*_adipose_, with:

2 $${\displaystyle \begin{array}{c}{PR}_{adipose}=\frac{CO\times {PCO}_{adipose}}{100}\end{array}}$$

These blood perfusion rates range with the following PCO ranges from 2 to 6.6 ml/min/100 g tissue. The IF flow to the adipose tissue can be given by *QIF*_adipose_ with

3 $${\displaystyle \begin{array}{c}{QIF}_{adipose}=\frac{\ {R}_{IF/B}\times {PR}_{adipose}}{M_{adipose}}\end{array}}$$

Typical values range between 3.8 [2.8–9.5] μL/h/g tissue for males to 4 [3–9.5] μL/h/g tissue for females. This IF flow is in agreement with measured values in the skin of rat and rabbit [4–6] μL/h/g [16]. This IF separates in lymph for 15% and the remaining volume 85% of the IF is reabsorbed by the venous capillaries within the tissue. Therefore, the flow of IF to Lymph or lymph flow (*Q*_Lymph_) is estimated at 0.57 [0.42–1.36] μL/h/g tissue in males and 0.6 [0.45–1.42] μL/h/g tissue in females. The venous return of IF (*QIF*_venous_) is then given by the difference, i.e., 3.25 [2.41–7.68] μL/h/g tissue in males and 3.42[2.53–8.07] μL/h/g tissue in adult females.

The ranges of blood perfusion and IF, lymph and venous return flow above are given for illustration of an average situation. In SubQ-Sim, the IF flow is calculated for each individual based on the revised Starling principle, following the exchanges across the capillaries between the plasma and the IF at the injection site. A basal healthy IF flow is calculated from basal cardiac output and partial cardiac output for the tissue of interest. The impact of disease and environmental factors, or “life events,” are incorporated into the model to calculate local IF flow as a function of time. These calculations are detailed in the supplementary materials Sections 12 to 19.

### Viscosity of the Interstitial Fluid

Interstitial fluid contains all the constituents of the plasma but at a lesser concentration for large solutes which are sterically hindered from extravasation from the plasma [17]. For albumin, the reported concentration ranges from 17 g/L vs 44 g/L for plasma [18]. The albumin concentration in the IF corresponds to approximately 40% that of the plasma [18] but for each plasma protein, the concentration ratio between the IF and plasma depends on the hydrodynamic radius of the protein [18] and the plasma filtration rate which can result in protein dilution. It is therefore not a constant value. Authors have explained this phenomenon through pore theories which represent the ultrafiltration of plasma components [19]. The adipose tissue also secretes a number of proteins and for these proteins, the concentration in the IF is higher than in the plasma [18]. All small electrolyte concentrations and pH are identical to plasma since they are small enough to pass through the pores of the capillary walls and their free concentration can equilibrate between plasma and IF. The IF density is reported at 1.02 g/mL with a viscosity of 1.5–2.2 mPa·s [13, 20]. The relative viscosity of IF compared to that of water is of 1.5 [21] to 1.6 [22]. Furthermore, disease states can influence protein concentrations in the IF. For example, Type I young diabetic subjects have a 1.6 fold lower concentration of albumin in the IF compared to healthy adults [23]. The main contributor to the viscosity of IF fluid is the free hyaluronan (HA), one out of four types of glycosaminoglycan. The concentration of HA was reported to be between 0.07 to 0.09 mg/g of wet ECM [20], whilst the concentration of HA measured in human lymph is around 9 μg/mL [24]. The serum HA concentration is markedly lower than the tissue level with an average of 0.22 μg/mL [0.1–0.4 μg/mL] in healthy humans [25]. HA aqueous solutions are Newtonian at low shear rates and then rheo-thinning above a critical shear rate of 3 s^−1^ [26]. During an injection into the adipose tissue, IF is displaced by the injected fluid towards the lymph. The IF fluid shear rate was shown to be less than 3 s^−1^ for more than 95% of the injection time up to 200 μL/s (Supplementary materials). Berriaud *et al.* proposed to fit the specific viscosity *η*_sp_ of hyaluronan aqueous solutions according to [26]:

4 $${\displaystyle \begin{array}{c}{\eta}_{sp}=\frac{\eta }{\eta_0}-1=C\left[\eta \right]+0.42\times {\left(C\left[\eta \right]\right)}^2+7.77\cdot {10}^{-3}\times {\left(C\left[\eta \right]\right)}^{4.18}\end{array}}$$

where *η* is the viscosity of the hyaluronan solution, *η*_0_ the viscosity of water, [*η*] the intrinsic viscosity of the hyaluronan solution in the Newtonian range (mL/g), and *C* the hyaluronan concentration (g/mL). This equation is close to experimental measurements in the range of *C*[*η*] from 0.1 to 46. The intrinsic viscosity of the hyaluronan solution is itself defined by, and has units inverse to, the polymer concentration.

5 $${\displaystyle \begin{array}{c}\left[\eta \right]=\frac{\eta -{\eta}_0}{{C\eta}_0}\end{array}}$$

[*η*] was found to be related to the hyaluronan molecular weight by the following relationship [26]

6 $${\displaystyle \begin{array}{c}\left[\eta \right]=1.56\cdot {10}^{-3}\times MW+125\end{array}}$$

with MW the hyaluronan molecular weight in g·mol^−1^ and [*η*] the intrinsic viscosity in mL/g. In human, molecular weights of hyaluronans between 1400 to 3300 kDa were reported by Tengblad *et al.* [24]. The HA concentration in humans is also affected by disease state: in type 2 diabetes (and not in type 1), the level of HA is multiplied by 1.5 [25]. Using the higher molecular weight reported for HA in human and the concentration ranges of HA in both lymph and adipose tissue for healthy humans and Type 2 diabetic subjects, the relative viscosity of lymph and adipose tissue IF to water can be calculated with the above equations (Table I).

**Table I:** Calculation of Relative Viscosity to Water for Lymph and Adipose Tissue Interstitial Fluid

Health and tissue	C HA (μg/mL)	MW HA g.mol-1	[η] (mL/g)	C[η]	η_rel_
Health lymph ^a^	0.2	3.30E+06	5.27E+03	1.05E-03	1.00
Health lymph ^a^	8.9	3.30E+06	5.27E+03	4.69E-02	1.05
Health lymph ^a^	18	3.30E+06	5.27E+03	9.49E-02	1.10
Health adipose tissue IF ^b^	70	3.30E+06	5.27E+03	3.69E-01	1.43
Health adipose tissue IF ^b^	130	3.30E+06	5.27E+03	6.85E-01	1.88
T2D lymph ^c^	0.2	3.30E+06	5.27E+03	1.05E-03	1.00
T2D lymph ^d^	11.125	3.30E+06	5.27E+03	5.87E-02	1.06
T2D lymph ^e^	27	3.30E+06	5.27E+03	1.42E-01	1.15
T2D adipose tissue IF ^f^	105	3.30E+06	5.27E+03	5.54E-01	1.68
T2D adipose tissue IF ^f^	195	3.30E+06	5.27E+03	1.03E+00	2.47

IF: Interstitial fluid, HA: Hyaluronan, T2D: Type 2 diabetes. a: HA concentration data from human samples from [24], b: HA concentration reported in [20], c: low health lymph data multiplied by 1, d: medium health lymph data multiplied by 1.25, e: high health lymph data multiplied by 1.5, f: health adipose tissue multiplied by 1.5

Values in Table I show that for Type 2 diabetic subjects, the relative viscosity of the IF in the adipose tissue could be 1.7–2.5 times higher than water.

### Variability in Blood and IF Flow

The adipose IF flow is directly related to the blood flow and can be subject to many sources of variation during the day, following changes in posture, sleep, exercise or, external temperature, after an injection, during stress or following a meal. Disease, especially obesity, changes the vasculature reaction to these external factors and also the baseline values. The following provides an overview of IF flow variability. In SubQ-Sim, these relationships are integrated quantitatively to provide for dynamic blood and IF flows through the capillaries as a function of time.

#### Physical Exercise

During physical exercise up to 50% of maximal capacity, the blood flow to the muscles and adipose tissue is multiplied by a ratio of 3–4 in healthy subjects [27–30]. The increase, above a baseline value, is found to be a linear function of exercise intensity. In type 2 diabetic (T2D) patients, this increase is only 1.2 fold [31].

#### Age

Age is another factor which is reported to reduce blood flow in the limbs. A 26% reduction in femoral blood flow was reported in men between 28 years and 63 years [32].

#### Temperature

Astrup *et al.* have studied the impact of external temperature on abdominal SC tissue blood flow [33]. There was a linear increase of the blood flow with skin or SC tissue temperature (Supplemental Fig. 29).

#### Stress

Linde *et al.* reported that the resting blood flow to the adipose tissue in the thigh and abdomen in 30 healthy young men are 3.0 ± 0.6 mL.min^−1^.100 g^−1^ and 7.6 ± 0.9 mL.min^−1^.100 g^−1^ respectively [34], and that mental stress (conflicting mental exercise) would cause these values to increase by 89% and 63% respectively.

#### Posture

The reference cardiac outputs provided in the section above were for a supine position. Blood or IF flow could be increased by 20% for standing or sitting subjects compared to a supine position [15].

#### Sleep

At night-time the IF flow is seen to increase from 80 to 200%.

#### Drugs

GLP-1 agonists elicit vasodilation and higher cardiac output. The abdominal adipose tissue blood flow is increased by 76% [35].

#### Locally at Injection Site

The variation of blood flow following injection shows a transient (1 hour) and localized increase in the blood flow around the puncture hole. A 7-fold increase in the local blood flow is reported which is observed within seconds following needle insertion. This increase in flow will decrease exponentially with a half-life of about 23 minutes [36].

#### Disease

Diabetic subjects show lower blood flow in the adipose tissue compared to healthy volunteers regardless of the site on their body [37]. At 35 °C, the blood flow in a diabetic subject is 62.4% that of a healthy subject (average from multiple locations). The increase of blood flow due to a raised temperature from 35 °C to 45 °C is approximately two-fold lower in diabetic subjects compared to healthy subjects (Supplemental Fig. 34). There is a suspicion that T2D patients who are insulin-resistant show issues with vasodilation which impairs their adaptation to external high temperature [38].

Other factors reported in the literature are the ingestion of food or the ingestion of carbohydrate rich liquids which increase the adipose tissue blood flow in healthy volunteers but not in obese subjects [39, 40]. In healthy subjects the consumption of a meal will roughly double the abdominal adipose tissue blood flow compared to fasted state. This blood flow will then reduce to the baseline fasted state value during the meal digestion (2–3 hours) [40]. In obese subjects, this increase in blood flow is much reduced and the lower capillarity in the tissue of obese subjects was used to account for this phenomenon mechanistically (see supplementary materials). The consequences of external factors on the absorption of insulin from subcutaneous tissue was illustrated by Berger *et al.* [41].

## The Injection and Resulting Depot

A subcutaneous injection is, first of all, a traumatic lesion locally disrupting the skin structure, bringing a foreign object (a needle), and foreign substances (the drug and excipients in the formulation), into contact with components of the innate immune system. Tissue injury triggers a standard inflammatory response involving a local reaction of the vasculature towards a disturbance of tissue homeostasis. Classical signs of tissue inflammation (redness and heat) reflect the enhanced blood vessel perfusion and permeability which follows the injection of the needle. This leads to the extravasation of plasma proteins and to the attraction of leukocytes to the inflamed region, and several biochemical cascades originating in the vascular compartment (e.g., complement, coagulation, and fibrinolytic systems) get access to the invaded interstitial space to combat potential foreign material, stop bleeding and start the tissue repair. Pain is also a common clinical reaction to injection due to direct nerve stimulation or indirectly through cytokine release. Via the concerted action of diverse endogenous mediators and immune cells at the injured site, potentially infectious agents (toxins) are washed out of the interstitial space, tissue debris are removed, tissue repair is initiated and, finally, inflammation is turned off [42]. The increased blood flow following injection is transient, lasting approximately 1 hour, and can be measured, for instance, with doppler imaging as shown by Anderson *et al.* after micro-dialysis probe injection [36]. Although the consequences for a modified release formulation could be limited, for an immediate release formulation, higher blood flow could contribute to earlier absorption. More clinical data is needed to measure skin blood flow at the injection site over time to refine our understanding of the differences observed in PK or also highlight the presence of injection site reactions beyond the transient period of 1 hour. Techniques such as doppler imaging or thermal imaging could be used to this effect.

### Subcutaneous Depot Shape and Characteristics

#### Depot Shape and Geometry

**Fig. 4:**
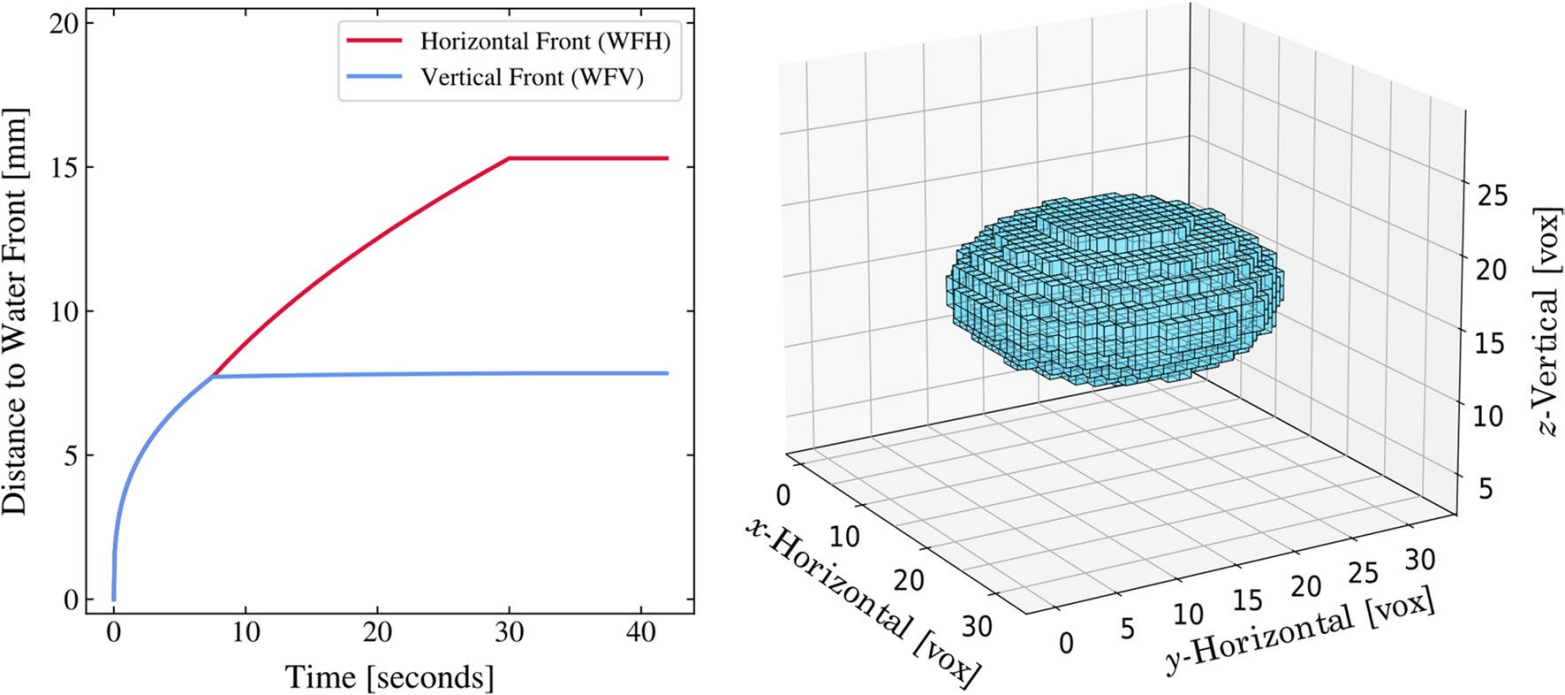
Depot shape in SubQ-Sim for a 1.2 mL injection over 30 seconds of saline. Left panel: Distance from needle tip to waterfront over time. Right panel: Aspect of the depot after injection. One voxel is a cubic spatial element of the tissue (32 voxels in each dimension).

The overall shape reported by various authors for the depots is ellipsoidal with marked differences in horizontal (perpendicular to the needle) versus vertical (parallel to the needle) depot dimensions. In order to accommodate the simulation of non-spherical depots, the injection site and the surrounding tissue are represented in the software by a 3-dimensional array of cubic voxels, typically 50 × 50 × 50. Since no major differences are reported for depot shapes in the plane perpendicular to needle, we will define following the approach by Kim *et al.* [43], a horizontal waterfront in this plane (*WF*_H_), which corresponds to the edge of the formulation depot (radius) and a vertical waterfront (*WF*_V_), which corresponds to the depot edge (radius) in the plane parallel to the needle. All these dimensions are taken from distance 0, which corresponds to the needle tip during injection (Fig. 4).

Kim *et al.* [43] examined the injection of 500 μL of a low viscosity insulin solution marked with iodine at 25 μL/min and 100 μL/min in porcine tissue and measured the relative content of the solution (RCS) in the SC space as a function of time and distance from the needle using real time 2D-X-ray imaging technique. The RCS is higher in the vicinity of the needle tip and decreases to zero at the waterfront, which represents the edge of the injected depot in the SC space. The waterfronts reported by Kim *et al.* in the vertical and horizontal directions over time, allow us to compute an average RCS (ε) using the equation below:

7 $${\displaystyle \begin{array}{c}\upvarepsilon =\frac{Q_{inj}\cdot {t}_{inj}}{V_{depot}}\end{array}}$$

where, *Q*_*inj*_ is the volumetric injection rate, *t*_*inj*_ is the total time taken for the injection and *V*_*depot*_ is the volume enclosed by the water fronts (*WF*_V_ and *WF*_H_):

8 $${\displaystyle \begin{array}{c}{V}_{depot}=\frac{4}{3}\pi \cdot W{F}_V^2\cdot W{F}_H\end{array}}$$

With a more rapid injection rate in subcutaneous tissue of 6 mL/min, the depot shape became more elongated with a waterfront in the horizontal direction which is twice that in the vertical direction. In addition, the RCS in the SC space stays more constant in the horizontal direction over a larger distance which attests for more tissue disruption by the injected fluid closer to the needle tip. The time dependence for tissue penetration is also much smaller in the vertical direction: The depot expands horizontally more than vertically over time. Experimental depot shapes were reported by Jockel *et al.* [44], who measured the shape and size of insulin Lispro® depots (viscosity of 1.106 mPa·s [45]) following injection in fresh porcine tissue within 15 min of slaughter. For the 20–100 μL injection volumes, the ellipsoid elongation ratio formed by the depot is relatively constant at 4.5 to 6 and the RCS ranged from around 0.24 to 0.31, i.e., relatively constant with volume. Comley *et al.* reported an RCS of 0.23 following a 500 μL injection of a water like fluid in porcine tissue [46]. Any RCS value over the IF adipose volume fraction of 0.1, would indicate that the tissue is distended. Conversely, when RCS values fall below the IF adipose volume fraction, it would indicate that the tissue porosity has been preserved and that the formulation has replaced the IF or mixed with the IF in the SC space. Doughty *et al.* [47] evaluated the size of blebs following injections in live swine of volumes ranging from 1 to 10 mL at injection rates from 167 to 12,000 μL/min. The RCS values calculated from the apparent depot size (size of bleb) were not affected by the liquid viscosity and did not depend on the volume or the injection rate. Average values of 0.14 ± 0.04 can be computed for the RCS from the work of Doughty *et al.* The data for depot elongation from Doughty *et al.* [47] and from Kim *et al.* [43] is plotted versus the product of injection volume (mL) and injection rate (μL/min) to the power 1/3 (Supplemental Fig. 40). This data can be fitted with the following equation:

9 $${\displaystyle \begin{array}{c}E(t)=0.574\times {\left(\frac{Q_{inj}\times t}{1000}\times {Q}_{inj}^{1/3}\right)}^{0.4828} \,\,\,\,\textrm{if}\ E(t)\ge 1\end{array}}$$

where *E*(*t*) is the depot elongation and *Q*_*inj*_ is the injection rate in μL/min.

The impact of viscosity on the shape of the depot and backpressure was evaluated by Allmendinger *et al.* [48]. For similar injected volumes, the width and length of the depot were similar and did not show a large dependence on injection rate. The RCS for the data reported by Allmendinger *et al.* is 3 times higher than that reported by Kim or Doughty, with a positive effect on the injection rate on the RCS but no impact of viscosity on the RCS. This data also indicates that the higher viscosity leads to variability in the shape of the depot for a given injection volume regardless of the injection rate.

The overall findings of these experiments are that porcine adipose tissue has less resistance to flow (mechanical resistance) horizontally than vertically. This may be a bias related to the shape of the needle tip which may favor fluid velocity on the horizontal axis. Comley at al. also show that adipocyte lobules are organized in beehive-like structures made of collagen which would confer more mechanical resistance to vertical deformation [49]. This anisotropy of the subcutaneous tissue explains that above a certain volume and injection rate, the depot formed in the tissue starts as a spherical shape and then assumes an ellipsoidal shape. The final formulation depot elongation in the SC space can be predicted using the empirical model described above as a function of total volume and injection rate.

The model for depot formation during the injection time can be summarized below:

10 $${\displaystyle \begin{array}{c}{V}_{app}(t)=\frac{V_{inj}(t)}{\varepsilon }=\frac{Q_{inj}\times t}{\varepsilon }=\frac{4}{3}\pi \times {WFH}^2\times WFV=\frac{4}{3}\pi \times {E}^2\times {WFV}^3\end{array}}$$

where ε is the RCS, *E* the elongation ratio, *WFH* the horizontal waterfront, and *WFV* the vertical waterfront. It can be shown that:

11 $${\displaystyle \begin{array}{c} WFV(t)=\sqrt[3]{\frac{3{Q}_{inj}\times t}{4\pi \varepsilon {E}^2}}\end{array}}$$

12 $${\displaystyle \begin{array}{c} WFH(t)=E(t)\times WFV(t)\end{array}}$$

The elongation ratio is calculated over time using Eq. 9. Typical simulations show the evolution of the depot over time or over injected volume with fixed flow. Simulations show that for a given injection volume, the injection rate will influence the shape of the depot. With high injection rates, the depot moves from a spherical shape to an ellipsoid as observed in the literature (Supplemental Fig. 41). This model can be verified using the kinetic data from Kim *et al.*, the static bleb size from Doughty *et al.* and the data of Allmendinger *et al.* for low viscosity fluids. The predicted vs measured depot dimensions using a fixed average RCS of 0.13 are shown in Supplemental Fig. 42.

#### Impact of RCS on Drug and Protein Concentration in the Depot

**Fig. 5:**
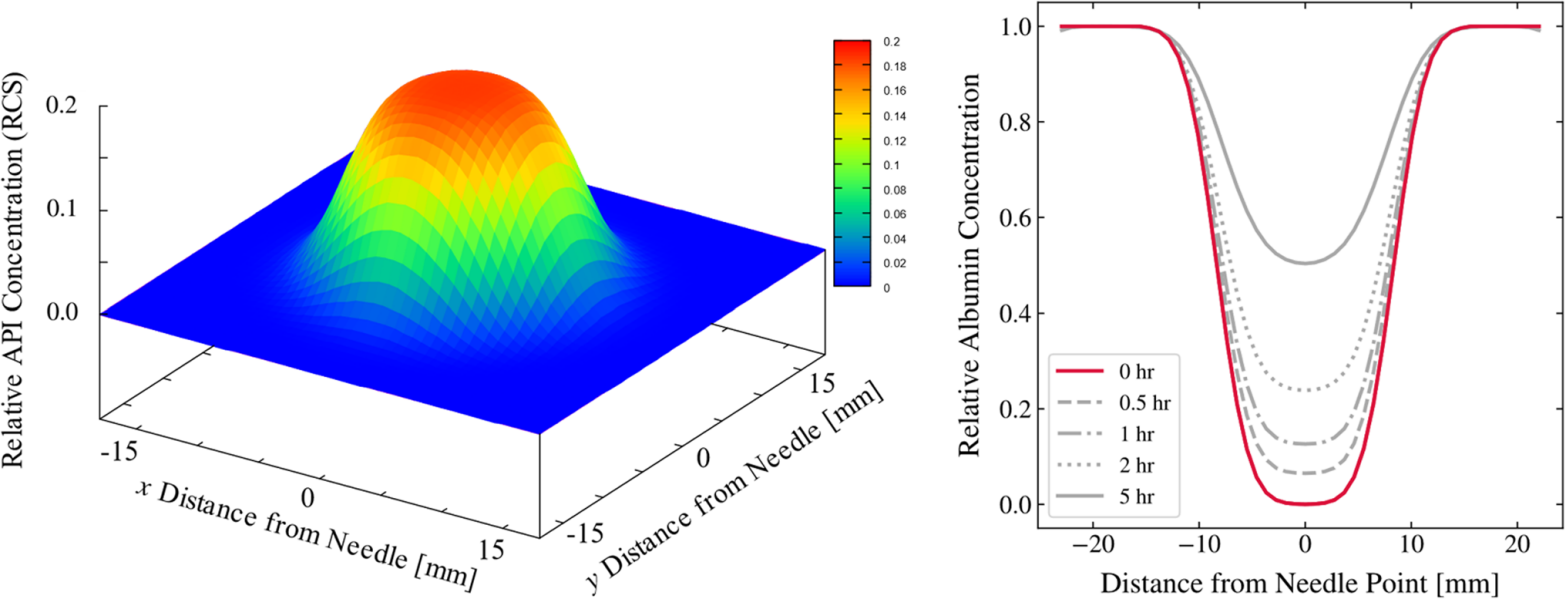
3D Plot of RCS following injection calculated in the horizontal plane (left panel) and protein concentration in the horizontal plane as a function of time (right panel).

In SubQ-Sim 2.0 although an average RCS is used to calculate the depot dimensions, the RCS evolution over distance from the needle tip is also computed. This enables the calculation of the drug concentration throughout the depot and the concentration of protein present in the interstitial fluid immediately after injection and over time, as the protein diffuses back from the surrounding tissue and trough the tissue capillaries. The hypothesis is that the mobile proteins and hyaluronan in the interstitium are flushed out during injection according to the model described below. The RCS measurements from Kim *et al.* [43] show that the drug concentration varies with the distance from the needle point, according to a sigmoidal change in concentration ranging from roughly 0.2 at the point of injection to close to zero as we approach the maximum extent of the waterfront. This concentration data, in both the horizontal and vertical directions, can be reasonably well approximated by fitting an equation of the form of:

13 $${\displaystyle \begin{array}{c} RCS(r)=\frac{RCS_{max}}{2}\left(1+\operatorname{erf}\left(\frac{\alpha WFX-\beta r}{WFX}\right)\right)\end{array}}$$

where erf denotes the Gauss error function, *α*, *β* and *RCS*_max_ are parameters which are derived from fitting, and *WFX* are the waterfronts in either horizontal or vertical directions (Supplemental Fig. 43), and r is the distance from the needle point. An overall scaling factor is then applied to the RCS to ensure that the enclosed mass of drug is exactly equivalent to the injected dose. This empirical equation is used only to establish the initial conditions for the SubQ-Sim model at the time point immediately after the injection. It is assumed that the injection time is relatively short such that the impact of diffusion and absorption via blood vessels and lymph is not significant during this short time period. The derivation of drug concentration in the voxels which comprise the formulation depot after the injection is detailed in the supplementary materials. The resulting illustration of drug concentration following injection in the ellipsoidal depot in the horizontal and vertical planes, is shown in Fig. 5. Proteins that are mobile in the interstitial fluid are assumed to be flushed from the depot and replaced with the drug formulation. The initial concentration profile follows the inverse of what is predicted for RCS i.e., where RCS is RCS_max_, the protein concentration is zero and where RCS is zero, the protein concentration is set to the baseline tissue value (Fig. 5). Over time following injection, the proteins can diffuse back to the depot from the surrounding tissue and from the arterial capillaries as they diffuse from the plasma. The concentration of drug and protein and therefore their ratio over time is dynamic in the model.

### Force Needed to Inject and Tissue Backpressure

#### Gliding Force

The gliding force needed to push the formulation through the needle using the syringe can be calculated according to the following equation [50]:

14 $${\displaystyle \begin{array}{c}F=\frac{32\eta L{Q}_{inj}{D}_S^2}{d^4}\end{array}}$$

where *η* is the dynamic viscosity, *L* is the length of the needle, *Q*_*inj*_ is the injection rate, *D*_*S*_ is the syringe internal diameter and *d* is the needle internal diameter.

#### Modeling Tissue Backpressure

Doughty *et al.* [47] studied the impact of viscous (13.6 mPa·s) and non-viscous (0.93 mPa.s) solution injections in porcine tissue, and measured the tissue backpressure from 1 mL to 10 mL injections at injection rates of 167 to 12,000 μL/min. In all these experiments the pressure was measured over time, and the data was normalized to eliminate the pressure profile when the injection system was actioned in air. The measurements were done *in vivo* in anesthetized domestic Yorkshire crossbred swine in the abdomen region. For all the conditions tested, the pressure increased initially with time up to a plateau, and then remained roughly constant for the rest of the injection duration. At the end of the injection the pressure dropped to a residual value. The raw data for plateau pressures are found in Supplementary Table 10. Other studies in porcine tissue were performed by Comley *et al.* [46], who reported the maximal backpressure for low viscosity liquids and Patte *et al.*, who performed a human clinical trial with low viscosity liquids at much lower infusion rates that the ones reported by the other authors, and reported the median and max backpressure values [51]. It appears that tissue backpressure increases linearly with injection rate and that the minimal pressure to inject is around 3.75 kPa, which could correspond to the yield pressure of the tissue extracellular matrix prior to tissue distension. The tissue backpressure during the injection could result from a combination of different factors: The dynamic pressure due to the liquid travelling in the pores of the SC tissue, the crack opening of the beehive ECM structures surrounding the adipocyte lobules and distension of the ECM to accommodate the formulation injected, and finally the muscle or skin distension due to the formation of a bleb in the SC tissue. Owing to the shape of the pressure time profiles immediately after injection, it is clear that even if the muscle or skin distension could contribute to the overall backpressure, this contribution must be small since the pressure drops rapidly to values below 3 kPa after injection of a 2 mL volume regardless of the injection rate [47]. The skin distension will therefore be used to calculate bleb residual pressure after the needle is removed from the injection site (see below). The data generated by Comley *et al.* [46] show that using a 27 or 21 gauge needle does not influence the tissue backpressure for a given injection rate. The data measured by Doughty *et al.* [47] and Allmendinger *et al.* [48] indicate that the volume injected does not seem to influence the tissue backpressure significantly after a certain time. This observation is not intuitive unless one hypothesizes that the tissue porosity is not constant and that the tissue opens up during injection to accommodate the depot volume such that the maximal resistance occurs at the edge of the depot where the tissue resistance is maximal. This hypothesis is also based on the observations of Kim *et al.* [43]: near the needle, the RCS is maximal and higher than the IF volume fraction in the subcutaneous tissue, which indicates that the depot “created” space in the tissue through opening and dilation of the extracellular matrix (ECM). Moving away from the needle, the RCS goes down and becomes less than the IF tissue volume fraction. This indicates that the formulation can penetrate the tissue without distending the ECM only towards the outer edge of the depot. At and beyond the waterfront of the depot, the RCS is equal to zero, the tissue is intact and does not contain the formulation. The mechanical tissue resistance to depot expansion during injection is therefore most likely to be mediated solely by the space immediately before the edge of the depot and inside the depot, where the tissue porosity is minimal. This hypothesis is analogous to the force needed to open a zip, which is constant and independent on time after the initial movement at given speed.

**Fig. 6:**
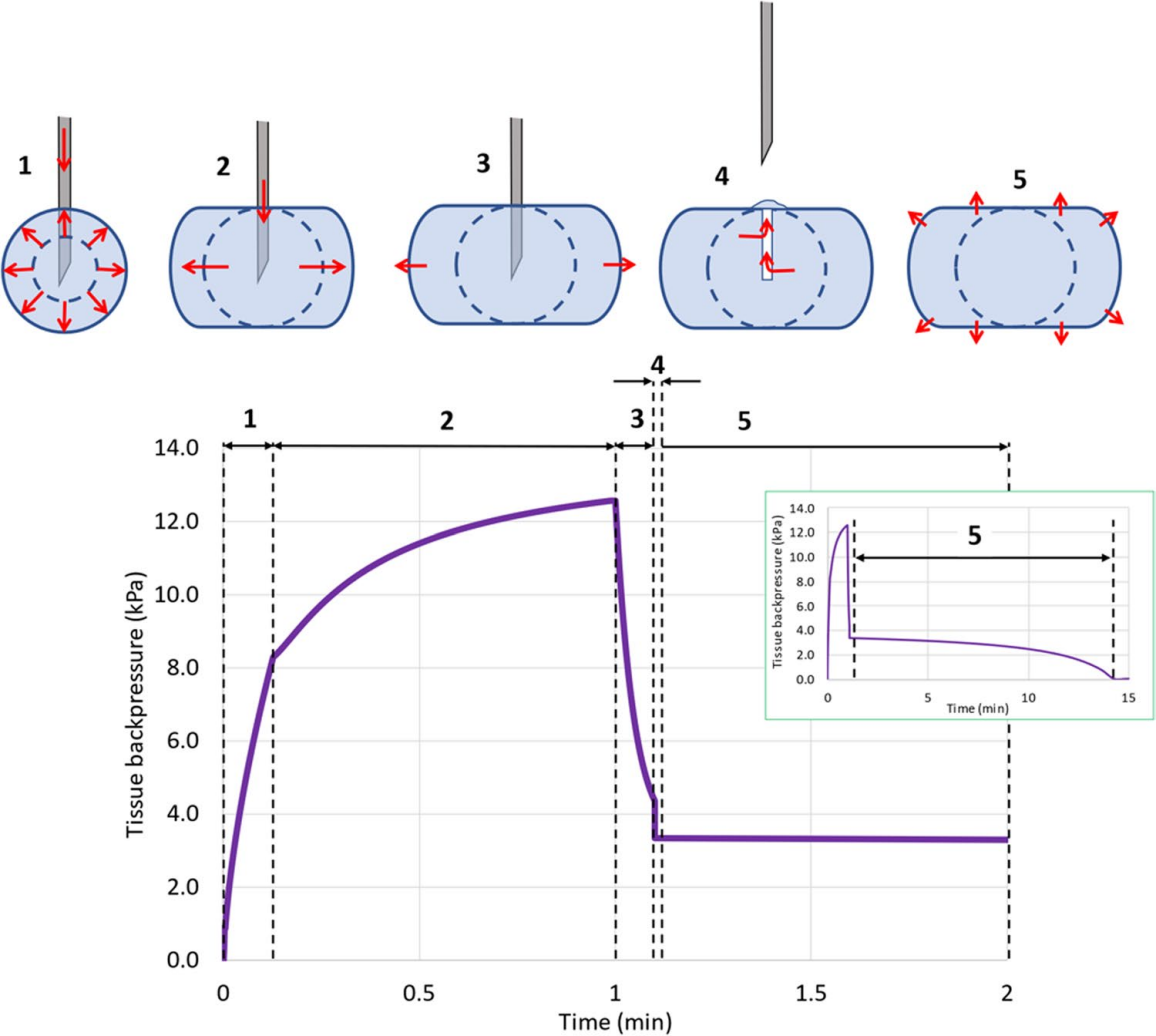
Schematic representation of the five phases of pressure recording in a subcutaneous depot and illustration of depot volume and shape changes. Insert represents longer simulation time.

The overall tissue backpressure during injection, post injection whilst the needle is still in place and during premature removal of the needle is illustrated in Fig. 6. Initially, the depot grows in all directions and the shape is spherical (phase 1 in Fig. 6). This phase could correspond to the build-up of pressure in the depot (the pressure increases with time or volume injected) and the formulation dilates the tissue in all directions. Then, the volume and injection rate cause the depot to elongate in the horizontal direction, but no further in the vertical direction, and the resistance to displacement becomes less volume dependent, if we hypothesize that only the horizontal edges of the ellipsoidal depot contribute to the tissue resistance (phase 2 in Fig. 6). This is the “zip” phase where the tissue backpressure shows less dependence on the volume injected. At the end of the injection when no more liquid is pumped into the tissue but whilst the needle is still in place, the pressure will drop since the formulation will diffuse, following the hydraulic conductivity of the tissue and the pressure difference (phase 3 in Fig. 6). Throughout the injection this diffusion is taking place but during phase 3 this is the only mechanism which explains the reduction in pressure. If the needle is prematurely removed during this latter phase, the residual pressure will be dissipated through the puncture hole that the needle has left in the tissue, since this is the path of least resistance to liquid flow. Phase 4 in Fig. 6 illustrates the backflow of formulation through the puncture hole and the rapid reduction of tissue pressure.

After a short time following needle removal, the puncture hole will collapse and the formulation which is trapped in the tissue will exert a residual pressure due to the bleb formation and skin elasticity. This phase 5 in Fig. 6 will last until the pressure goes back to zero due to slow fluid diffusion from the injected depot to the surrounding tissue. We will discuss how we model these 5 phases of tissue backpressure in the next sections.

##### Tissue Backpressure during Injection (Phases 1–2)

To model the tissue backpressure build up during phases 1 and 2 and post injection during phase 3, whilst the needle is still in place, equations based on the specific hydraulic conductivity of the tissue can be proposed. The specific hydraulic conductivity of the rat subcutaneous tissue was measured using a cell transfer model described by Swabb *et al.* [52]. The value for k was of 7.12E-18 m^2^ with an RSD of 29% on *n* = 3 samples of subcutaneous tissues from freshly sacrificed rat. Levick [53] proposed equations to calculate the tissue permeability on the basis of the extra-cellular matrix composition in terms of glycosaminoglycan (GAG), collagen and proteoglycan content and properties. The author used the Carman-Kozeny equation which relates the specific hydraulic conductivity of the tissue *K*, to the fractional tissue void volume *θ*_*EC*_, the wetted surface area per unit volume *S* (m^−1^), and the Kozeny factor *G* (dimensionless):

15 $${\displaystyle \begin{array}{c}K=\frac{\theta_{EC}^3}{G{S}^2}\end{array}}$$

An equation was proposed by Happel and Brenner to express the Kozeny factor for a random arrangement of cylindrical fibres and is shown below [53]:

16 $${\displaystyle \begin{array}{c}G=\frac{1}{3}\times \frac{2{\theta}_{EC}^3}{\left(1-{\theta}_{EC}\right)\left\{2 \ln\left(\frac{1}{1-{\theta}_{EC}}\right)-3+4\left(1-{\theta}_{EC}\right)-{\left(1-{\theta}_{EC}\right)}^2\right\}}\\ {}+\frac{2}{3}\times \frac{2{\theta}_{EC}^3}{\left(1-{\theta}_{EC}\right)\left\{ \ln\left(\frac{1}{1-{\theta}_{EC}}\right)-\frac{1-{\left(1-{\theta}_{EC}\right)}^2}{1+{\left(1-{\theta}_{EC}\right)}^2}\right\}}\end{array}}$$

The *G* calculated for a *θ*_*EC*_=0.103 (See Table II) is 4.84.

**Table II:** Calculation of Specific Hydraulic Conductivity for the Subcutaneous Tissue

Fiber	GAG	Proteoglycan	Collagen	Cells	IF
Fiber radius (m) [53]	1.48E-07	1.16E-07	3.70E-08	5E-05	NA
% w/w ECM mass [20, 53]	0.26	0.55	15.1	NA	NA
Amount of fluid taken up by the fiber (mL/g) [53]	0.65	0.65	1.735	NA	NA
Volume fraction in the tissue	2.37E-04	5.01E-04	3.67E-02	8.60E-01	1.03E-01
S (m^−1^)	3.20E+03	8.63E+03	1.98E+06	3.44E+04	NA
K (m^2^)	2.18E-11	2.99E-12	5.67E-17	1.88E-13	NA
1/K (m^−2^)	4.58E+10	3.34E+11	1.76E+16	5.31E+12	NA

The wetted surface area for each fiber can be given by:

17 $${\displaystyle \begin{array}{c}S=\frac{2\phi }{r_f}\end{array}}$$

where *ϕ* is the fractional fibre volume occupation of the space where diffusion occurs and r_f_ is the fibre radius. The apparent K (K_app_) for a tissue where multiple fibers are present is given by:

18 $${\displaystyle \begin{array}{c}{K}_{app}=\frac{1}{\sum_i\left(\frac{1}{K_i}\right)}\end{array}}$$

The typical values needed to calculate the apparent specific hydraulic conductivity for the subcutaneous space with data from Levick [53] and Wiig *et al.* [20] are shown in Table II.

The apparent specific hydraulic conductivity for the subcutaneous tissue is therefore given at 5.67E-17-m^2^ and is only determined in this calculation by the action of collagen fibers which have the smallest radius. This value is less than 10 fold higher than the specific hydraulic conductivity measured by Swabb *et al.* [52] reported above, and the average specific hydraulic conductivity of the intact SC tissue will be taken at 5.67E-17 m^2^.

Our simulations showing the time at which the vertical growth of the depot is halted (based on Eq. 9) align well with the observations made by Doughty *et al.* and Kim *et al.* on their dynamic pressure-profiles measurements for when the plateau in backpressure is reached, i.e., the transition between phase 1 and phase 2 in Fig. 6. This indicates that the tissue backpressure built-up only occurs during phase 1 in Fig. 6. To model tissue backpressure, we utilized the Darcy’s law, which relates the flow rate *Q* (m^3^/s) through a cylinder of length *L* (m), and surface area *A* (m^2^), to *k*, the specific hydraulic conductivity (m^2^), the liquid dynamic viscosity *η* (Pa·s) and the pressure difference between each end of the cylinder Δ*P* (Pa).

19 $${\displaystyle \begin{array}{c}Q=\frac{kA\Delta P}{\eta L}\end{array}}$$

If the subcutaneous tissue resistance to movement occurs through the volume where the RCS is above 0 but less than the IF volume fraction in the tissue, then this volume is anticipated to be a small spherical “onion ring” at the edge of the depot. In addition, the interstitial fluid is pushed away from the advancing front of the depot, through the extracellular matrix system of the intact subcutaneous space. The viscosity of the fluid which is moved in this high resisting onion ring is anticipated to be a mixture between the formulation viscosity (*η*_*formulation*_) and IF viscosity (*η*_*IF*_). An RCS level of 0.1 is used to calculate the apparent viscosity of this fluid mixture (assuming that the mixture is immediate):

20 $${\displaystyle \begin{array}{c}{\eta}_{app}=0.1\times {\eta}_{formulation}+0.9\times {\eta}_{IF}\end{array}}$$

The pore area (*A*_*pore*_) for liquid flow is anticipated to be constant, and the length of the “tube” in the Darcy’s law is anticipated to be proportional to the surface area of the depot. Since the pores are found at the surface on the edge of the depot, we have assumed that these pores can be put together in series to increase the length L through which the fluid has to flow to simulate the increasing resistance to flow that the advancing waterfronts create. Equation 19 can therefore be rewritten as:

21 $${\displaystyle \begin{array}{c}P(t)=\frac{Q_{inj}{\eta}_{app}L(t)}{k{A}_{pores}}\end{array}}$$

To capture pressure build up during phase 1 and relative constancy after the depot starts to elongate (phase 2), the value of *L*(*t*) is anticipated to be proportional to the spherical portion of the depot: 4*π* × *WFV*(*t*)^2^. A proportionality factor (*F*) in m^−3^ is introduced which expresses the length to surface ratio divided by the pore area. The equation predicting pressure evolution during phase 1 and 2 is therefore:

22 $${\displaystyle \begin{array}{c}{P}_{1-2}(t)=\frac{F\times {Q}_{inj}{\eta}_{app}4\pi WFV{(t)}^2}{k}\end{array}}$$

With this equation, *P*_1 − 2_(*t*) is integrated over time to provide for a backpressure vs time profile. *F* was correlated to values reported by Doughty *et al.* [47] and was estimated with the following equation where *V*_*inj*_ is the volume injected in mL and *Q*_*inj*_ the injection rate in μL/min:

23 $${\displaystyle \begin{array}{c}F=2\ \ln\left(\frac{Q_{inj}}{{V_{inj}}^2}\right)+5\end{array}}$$

In addition to tissue resistance to flow, the buildup of a bleb under the skin, combined with skin’s elasticity will create a small additional pressure which will contribute to the backpressure during and after injection (phase 1–5 of Fig. 6). This bleb pressure (*P*_*b*_) will also be the last measurable pressure during phase 5. It is simply computed by the angular skin deformation (*δ*), and skin stiffness (*St*_*skin*_):

24 $${\displaystyle \begin{array}{c}{P}_b(t)=\delta (t)\times {St}_{skin}\end{array}}$$

Supplementary Section 5 details how the skin stiffness is measured and predicted based on population covariates and injection sites. During phases 1 and 2 the pressures due to tissue resistance to flow and bleb pressure are summed.

Throughout this phase, liquid from the depot is allowed to flow outside of the depot towards the tissue and therefore reduce its apparent size. This flow of fluid which starts immediately after the start of injection and as long as there is pressure exerted, i.e., even after the end of injection, is governed by Eq. 26.

The average fold error for backpressure calculation of experimental data using Eq. 22 is 1.01. The average prediction error is 44% which is less than the average variability in the measurements (66%) (Supplemental Fig. 47). Allmendinger *et al.* [48] measured a force from which the pressure can be calculated based on the diameter of the syringe utilized in their experimental setting. With viscous solutions used by these authors and especially at high injection speed, it was difficult to differentiate the tissue backpressure from the pressure needed to push the formulation through the injection device in air, which could explain why the tissue backpressure is overestimated with the current model.

##### Tissue Backpressure Post Injection with Needle in Place (Phase 3)

Immediately after the end of injection the pressure diminishes as the fluid continues to distribute in the surrounding tissue. This process lasts a few seconds and contributes to further apparent volume occupation for the depot. This phenomenon is more pronounced for large volume injections as shown by Koulakis *et al.* [54]. In the proposed model, it is hypothesized that the pressure build-up is related to only a small portion of the depot from the surface. It is assumed that 2.5% of the depot’s total volume at the edge of the depot will comprise fluid under stress. This stressed fluid volume is therefore calculated by:

25 $${\displaystyle \begin{array}{c}{V}_{stress}(t)=0.025\times \frac{4}{3}\pi\ \varepsilon WFH{(t)}^2\times WFV(t)\end{array}}$$

The flow of fluid from the surface of the depot towards the intact tissue is expected to happen through the surface area of the entire depot. Similar to the previous equation for pressure build-up during phases 1 and 2, the tissue relaxation (fluid flow in the tissue) is expressed with the following equation:

26 $${\displaystyle \begin{array}{c}Q(t)=\frac{k\times \left({P}_{1-2}(t)+{P}_b(t)\right)}{F\times {\eta}_{app}\times 4\pi {\left(\frac{WFH{(t)}^{3.2}+2{\left( WFH(t) WFV(t)\right)}^{1.6}}{3}\right)}^{\frac{1}{1.6}}}\end{array}}$$

The pressure after injection (at *t*_*end*_) and until time of needle removal (*t*_*rem*_) is given by:

27 $${\displaystyle \begin{array}{c}{P}_3(t)={P}_{1-2}\left({t}_{end}\right)\times \left(1-\frac{\int\limits_{t_{end}}^{t_{rem}}Q(t)\, \mathrm{d}t}{V_{stress}\left({t}_{end}\right)}\right)+{P}_b(t)\end{array}}$$

**Fig. 7:**
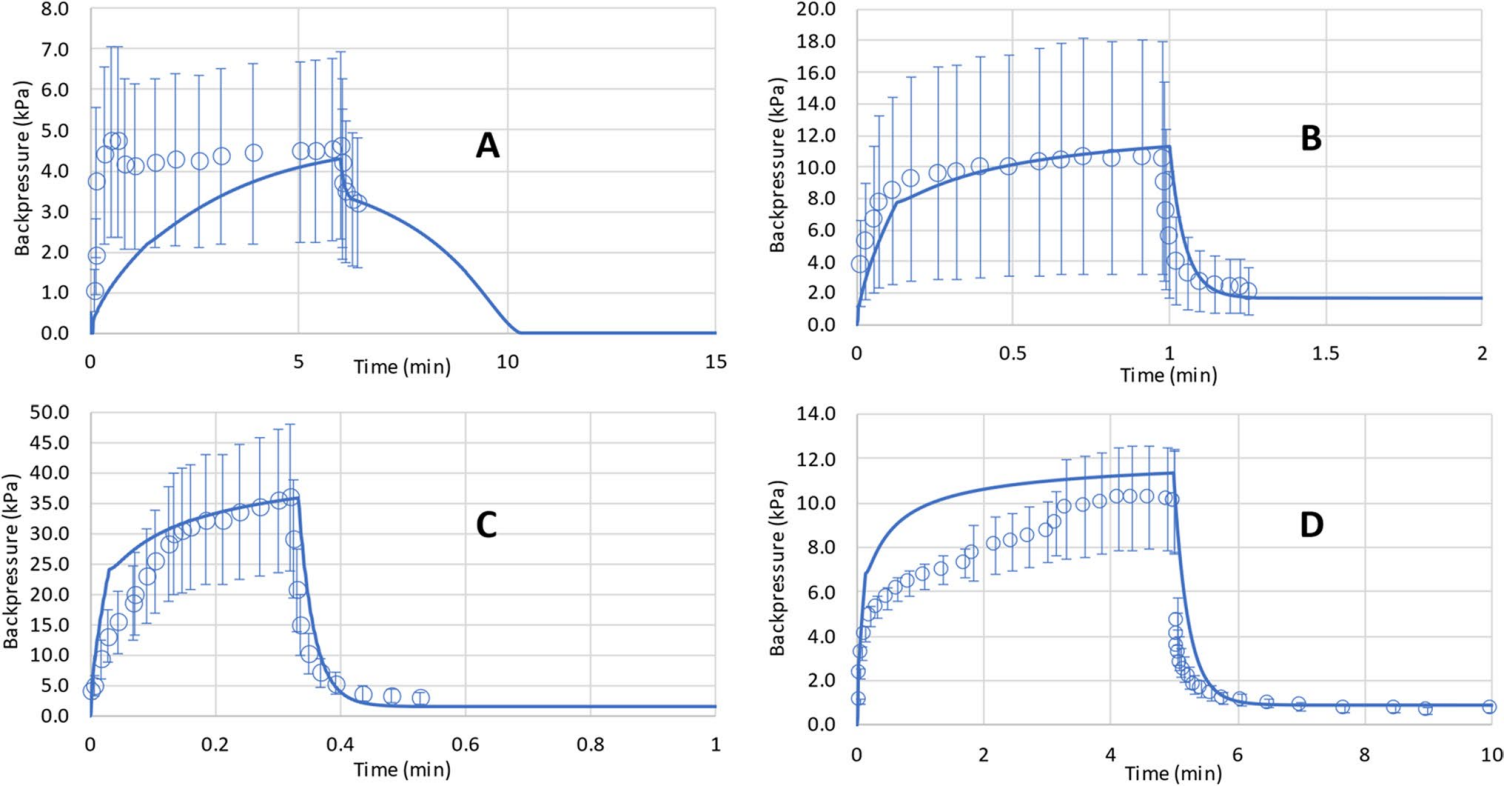
Predicted and measured pressure time profiles for 2 mL saline injections in anesthetized Yorkshire crossbred pigs at (**A**) 20 mL/h, (**B**) 120 mL/h, and (**C**) 360 mL/h *n* = 3 from [47], and for 10 mL 7.2 mPa.s IgG solution injections in anesthetized Yucatan minipigs at 120 mL/h (**D**) *n* = 24 from [55].

Figure 7 shows the pressure profile predictions for pig live injections generated from the set of above equations and the values measured by Doughty *et al.* [47] and Kang *et al.* [55].

##### Premature Removal of the Needle Post Injection (Phase 4)

One advantage of the mechanistic model above is that the impact of premature needle removal on the formulation backflow can be predicted. The needle is assumed to have punctured the skin to a distance equivalent to its length and with a diameter equivalent to the needle external diameter regardless of the injection angle. If the needle is removed at any time during injection or after injection, there may be residual pressure in the tissue which can lead to formulation leakage through the puncture cylinder. The puncture hole is allowed to leak fluid for ∆*t *= 25 milliseconds.

The dead volume of the needle puncture cylinder is given by:

28 $${\displaystyle \begin{array}{c}{V}_{dead}=\pi {L}_N{R}_{ext}^2\end{array}}$$

where *L*_N_ is the needle length and *R*_ext_ is the external needle radius. The flow rate through this cylinder is given by the Hagen–Poiseuille equation:

29 $${\displaystyle \begin{array}{c}Q=\frac{\Delta P\pi {R}_{ext}^4}{8\eta {L}_N}\end{array}}$$

The loss of volume through this puncture hole is therefore given by:

30 $${\displaystyle \begin{array}{c}{V}_{loss}(t)=\int\limits_{t_{rem}}^{t_{rem}+\Delta t}\frac{P(t)\pi {R}_{ext}^4}{8{\eta}_{form}{L}_N}\ \mathrm{d}t+{V}_{dead}\end{array}}$$

where *t*_*rem*_ is the time of needle removal from the injection site. The above equation assumes that the formulation volume inside the puncture hole is expelled from the body as the puncture hole collapses. The impact of this additional volume loss on tissue backpressure is calculated from:

31 $${\displaystyle \begin{array}{c}{P}_4(t)={P}_3\left({t}_{rem}\right)\left(1-\frac{\int\limits_{{\textrm{t}}_{end}}^{t_{rem}+\Delta t}Q(t)\ \mathrm{d}t+\int\limits_{{\textrm{t}}_{rem}}^{t_{rem}+\Delta t}\frac{P(t)\pi {R}_{ext}^4}{8{\eta}_{form}{L}_N}\ \mathrm{d}t}{V_{stress}(t)}\right)+{P}_b(t)\end{array}}$$

The volume loss during premature removal of the needle is used to recalculate bleb deformation and bleb pressure. The percent dose loss due to needle removal can be calculated from the volume loss. If the removal happens before the end of the injection time, the volume still contained in the device is added to the dose lost from the puncture hole:

32 $${\displaystyle \begin{array}{c}{P}_{loss}\left(\%\right)=\frac{V_{device}+{V}_{loss}\left({t}_{rem}+\Delta t\right)}{V_{inj}}\times 100\end{array}}$$

**Fig. 8:**
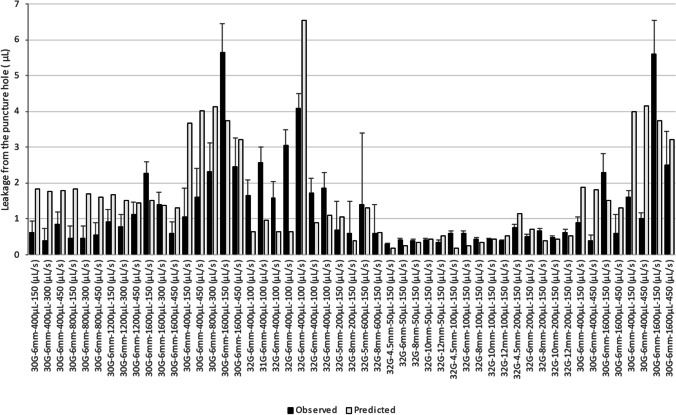
Predicted and measured leakage through puncture holes. Data from Supplemental Table 11.

The validation of formulation losses through the puncture hole by tissue backflow was done on the basis of data generated by Præstmark, *et al.* [56], Ignaut *et al.*, [57], Wittmann *et al.* [58], Heise *et al.* [59], in diabetic patients and in pigs. In humans two injection sites were chosen (abdomen and thigh) that were tested by Heise *et al.* and Præstmark, *et al.* In the model, skin stiffness relative to these two injection sites is changed to account for differences in bleb related pressure (Supplemental Section 5). For diabetic patients, they were all assumed to be type 2 diabetic subjects and the IF viscosity was taken at 2.08 mPa.s. For pigs, the IF viscosity is taken at 1.66 mPa.s, which correspond to a healthy human average value (Table I). The raw data for model validation is presented in Supplemental Table 11. The predictions results are shown in Supplemental Table 11 and Fig. 8.

**Fig. 9:**
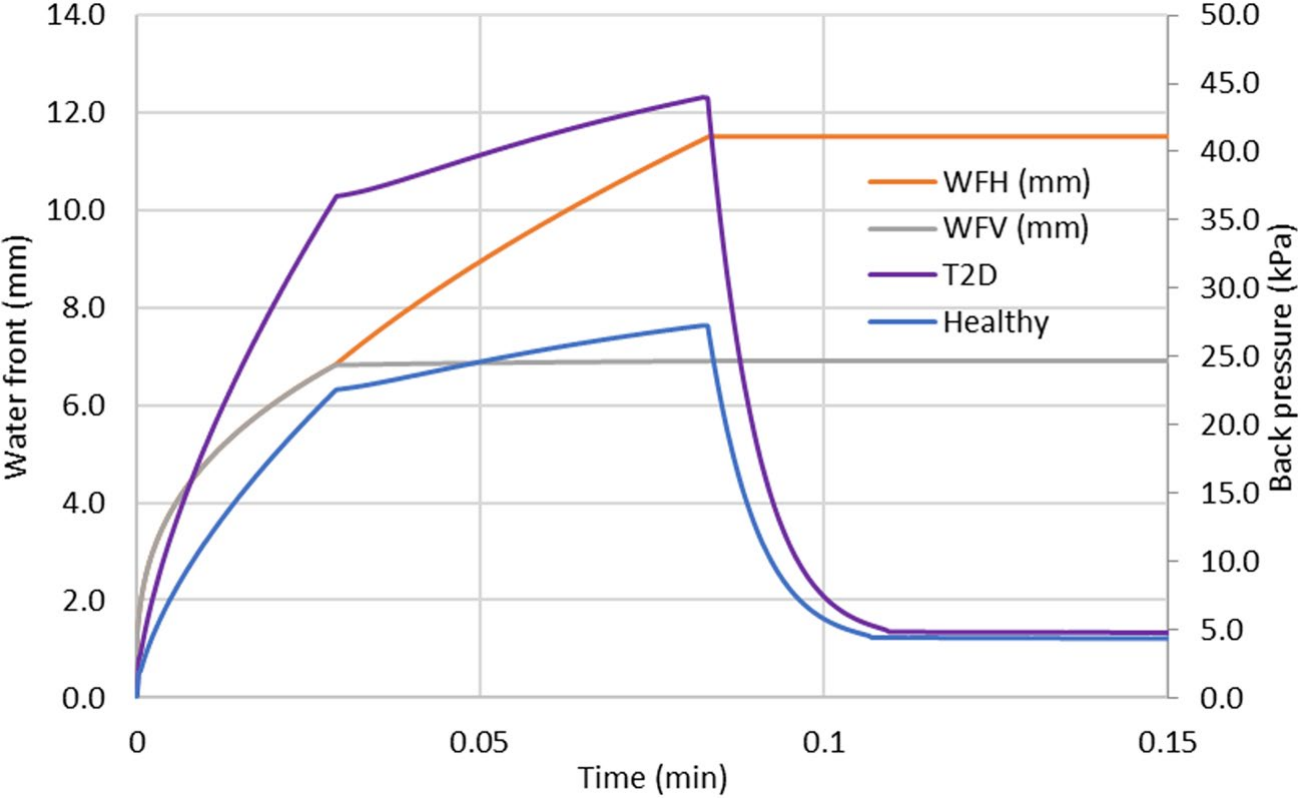
Predicted tissue backpressure in a type 2 diabetic and healthy subject using the injection parameters of Table III and the extreme relative interstitial fluid viscosities of Table III.

The AFE for leakage prediction is 1.19. The absolute average prediction error is 47%. In Supplemental Table 11, it is observed that there is always a small leakage, which corresponds to the amount of formulation found in the puncture hole. Is it assumed that the puncture hole closes itself quickly after needle removal and that the volume present in the hole is lost outside of the body. This represents a small amount of fluid, typically around 0.5 μL. It is also interesting to see the higher the volume injected, the larger the leakage. In addition, higher injection rates for similar injection volumes lead to lower leakage. The losses in the puncture holes are adequately predicted by the model for various configurations of needle gauges and lengths (Fig. 8). Also, the reduction of leakage when the injection rate increases for a given volume is adequately predicted by the model. This is related to the higher pressure existing at the end of injection which pushes more liquid inside the tissue per unit time during the relaxation phase compared to lower injection rates. For very slow injections the loss is restricted to that of the puncture hole. This is aligned with recommendations to minimize leakage by injecting slowly [60]. A small needle length or large needle diameter are associated with greater losses. The formulation viscosity does not impact the formulation backflow since the pressure increases proportionally with viscosity whilst the flow through the puncture hole is inversely proportional to the viscosity. The impact of disease in predicted tissue backpressure was illustrated by simulating the injection of 0.5 mL saline at 6 mL/min using a 6 mm 32Gauge needle (Table III), in healthy or type 2 diabetic (T2D) subjects (Fig. 9).

**Table III:** Parameters for Simulation of Impact of Disease on Tissue Backpressure

Parameter	Symbol	Value	Units
Injection Volume	*V*_inj_	500	μL
Injection Rate	*Q*_inj_	6000	μL/min
Injection Time	*t*_inj_	5.0	s
Needle External Radius	*R*_ext_	0.311	mm
Needle Internal Diameter	*d*	0.318	mm
Needle Injection Angle	*α*	90	Degrees
Needle Length	*L*_N_	6.0	mm
Syringe Barrel Internal Diameter	*D*_s_	12.07	mm
Formulation Viscosity	*η*_form_	1	mPa·s
Formulation Density	*ρ*_*form*_	1000	kg / m^3^

As seen in Fig. 9, the tissue maximum backpressure in a T2D subject is anticipated to be 60% higher than in a healthy subject. This is related to the difference in hyaluronan concentration in these populations in the interstitial fluid (Table I).

##### Residual Pressure Post Needle Removal (Phase 5)

In the pressure time measurements carried out by Doughty *et al.* [47], after the end of the injection and tissue relaxation, and whilst the pressure measuring device was still in place the pressures did not return to their baseline value and a residual pressure of around 3 kPa was measured in the pig. This residual pressure can be related to the size of the bleb under the skin, which leads to skin deformation and results in exerted pressure. The injected formulation creates a strain in the subcutaneous space which leads to a residual pressure. Over time, the fluid will dissipate in the tissue and the residual backpressure will subside. To model the residual pressure, the skin deformation due to the presence of a depot is calculated and used with the skin stiffness (Supplemental Section 5) to estimate the pressure on the depot. As seen in Supplemental Section 5, the predicted bleb residual pressures predicted from skin deformation and skin stiffness are not significantly different from the data measured by Doughty *et al.* [47]. Since these predictions were obtained using human skin stiffness data, they would have to be redone with animal skin stiffness measurements. However, these predictions are encouraging and additional pressure data in animal or human together with skin stiffness measurement at the site of injection would be needed to characterize the prediction ability for this model.

#### Modeling Time-Dependent Effect of Hyaluronidase on Backpressure

The impact of hyaluronidase on injection backpressure can be modelled mechanistically since it is expected to reduce the molecular weight of hyaluronan (HA) and therefore facilitate the flow of interstitial fluid during the injection step. Recombinant human hyaluronidase PH20 (rHuPH20) has a short half-life once it has reached the systemic circulation, a molecular weight of 61 kDa and an activity unit to μg dose ratio of 110 [61]. Once injected in the subcutaneous space, rHuPH20 will induce a rapid degradation of the hyaluronan contained in the extracellular space of the subcutaneous tissue, producing fragments of 20 kDa size. The rHuPH20 will then be eliminated from the tissue, leading to hyaluronan replenishment in 24–48 hours [61]. Fang *et al.* measured the kinetic degradation of large molecular weight hyaluronan with rHuPH20 [62]. From their measurements, they determined catalytic constants of *V*_max_ = 1.7 nM·s^−1^, *K*_m_ = 0.9 mg/mL and *k*_cat_ = 41 s^−1^. The *in-situ* degradation of hyaluronan can therefore be modelled with the following equation:

33 $${\displaystyle \begin{array}{c}v(t)=\frac{\left[ HA\right](t)\times {V_{max}}}{\left[ HA\right](t)+{K}_m}\end{array}}$$

The 20 kDa fragments produced by the catalytic action of rHuPH20 on HA ([*P*](*t*)) are calculated by:

34 $${\displaystyle \begin{array}{c}\left[P\right](t)=\int\limits_0^tv(t)\, \mathrm{d}t\end{array}}$$

The remaining concentration of HA is given by:

35 $${\displaystyle \begin{array}{c}\left[ HA\right](t)={\left[ HA\right]}_0-\int\limits_0^tv(t)\, \mathrm{d}t\end{array}}$$

The apparent molecular weight of hyaluronan in the tissue is given by:

36 $${\displaystyle \begin{array}{c} MW(t)=\frac{\left[ HA\right](t)\times {MW}_0+\left[P\right](t)\times {MW}_f}{{\left[ HA\right]}_0}\end{array}}$$

where *MW*_0_ is the initial molecular weight of the HA in the tissue, which for the SC tissue is taken at 330 kDa, and *MW*_*f*_ is the catalytic product (fragment) molecular weight of 20 kDa. This molecular weight is also used to correct the pressure factor in Eq. 22 using the following equation which is valid until the HA molecular weight has reached a value of 10^6^ Da (*MW*_*end*_).

37 $${\displaystyle \begin{array}{c}F(t)=\frac{F\times MW(t)}{MW_0}\end{array}}$$

This value is arbitrary and further model development beyond the scope of this article could explain mechanistically the impact of mechanical interlocking of large HA molecules in the collagen mesh formed in the extracellular matrix. Indeed, this pressure factor accounts for fixed pore size and length to make use of the Darcy equation and relate pressure to solution viscosity. As shown in Table I, the IF viscosity shows only a minor increase compared to that of water at the same temperature, and the mechanical resistance to flow in normal conditions is comprised in the pressure factor F.

**Fig. 10:**
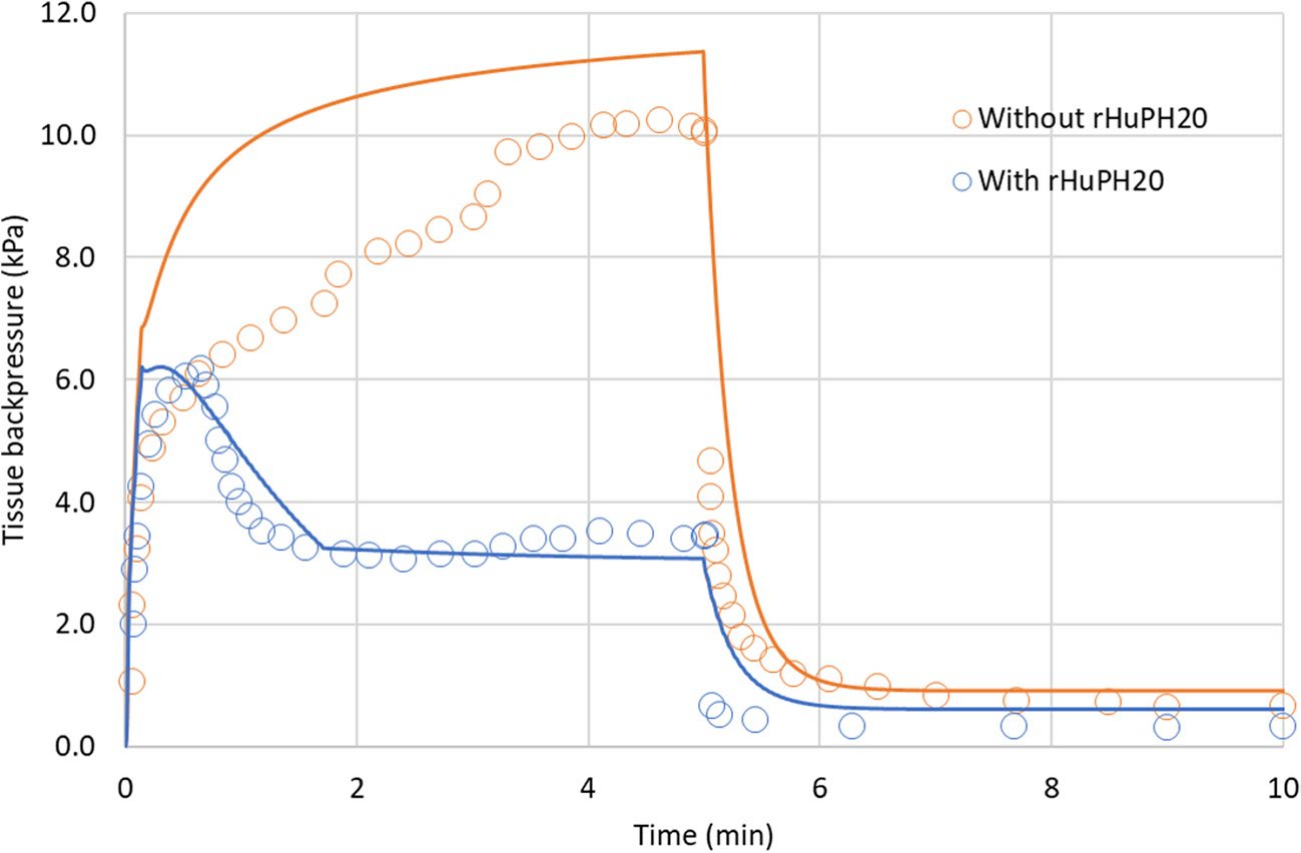
Backpressure time profile prediction for a 10 mL injection of a 7.2 mPa.s solution at 2 mL/min with and without 25,000 U/mL rHuPH20 in healthy subjects.

Kang *et al.* [55] measured the tissue backpressure following injection of 10 mL of a 7.2 mPa·s IgG solution in pigs with and without 25,000 U/mL rHuPH20 at 2 mL/min. The pressure time profile was initially comparable between the two formulations, but after approximately 50 seconds for the formulation comprising rHuPH20, the tissue backpressure dropped to stabilize around a value three times less than that observed for the reference formulation without rHuPH20. This experiment was modelled with the SubQ-Sim model for backpressure. The original *V*_max_ measured by Fang *et al.* was subjected to a sensitivity analysis by multiplying it by a factor of up to 10. In addition, the injection rates were also varied by 4-fold (See Supplemental Fig. 49). The proposed model with a 2-fold higher *V*_max_ than measured by Fang *et al.* is shown in Fig. 10.

The starting concentration of hyaluronan in the interstitial fluid was taken at 100 μg/mL, i.e., the average concentration for a healthy human (Table I). Interestingly, the qualitative shape of the pressure vs time profile observed by Kang *et al.* in minipigs is well reproduced by the model for both formulations. In the formulation comprising rHuPH20, the pressure initially follows that observed for the formulation without hyaluronidase. Then there is a decrease of pressure until another plateau is observed related to the reduction in hyaluronan molecular weight.

## Discussion and Outlook

**Fig. 11:**
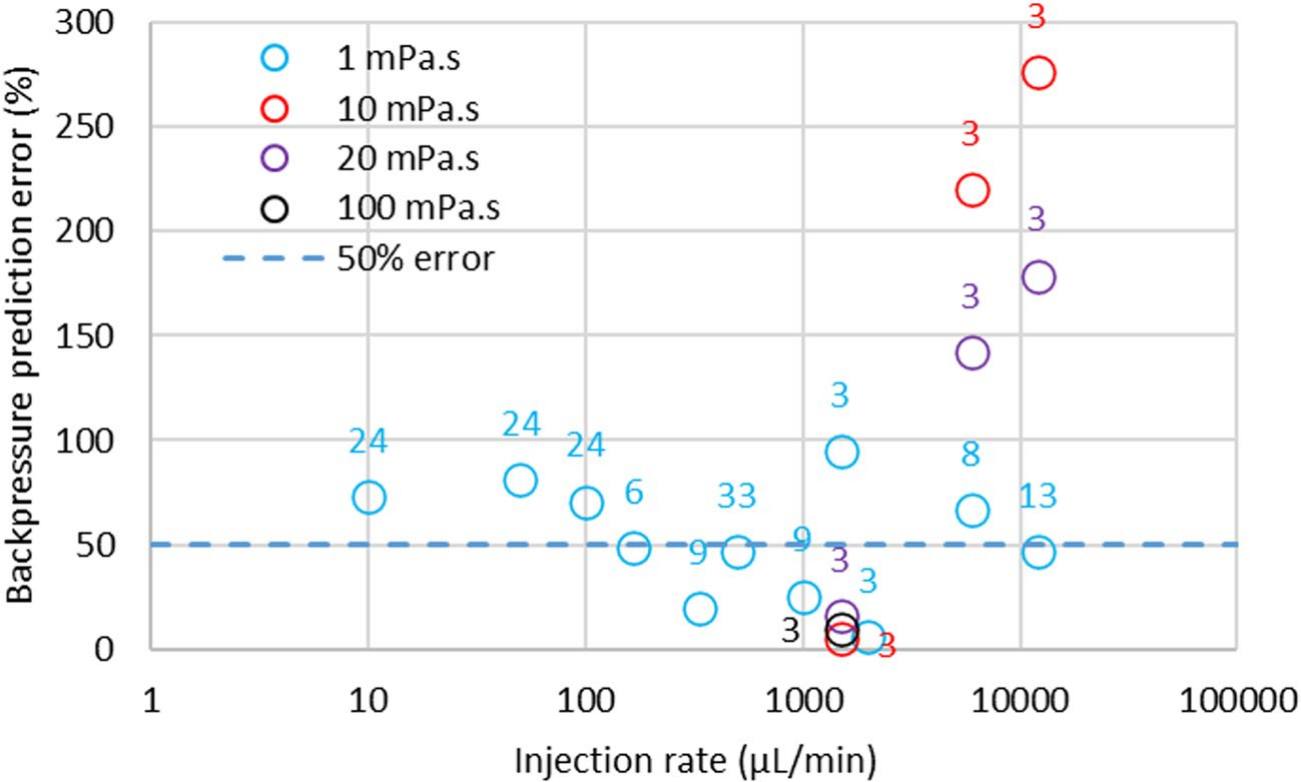
Backpressure prediction errors as function of injected solution viscosity and injection rate. Number of experiments appear on the graph next to symbols.

This article starts to describe the construction of a physiologically based biopharmaceutics model for the subcutaneous space, which can be applied to each subject in both healthy and diseased states. The focus for this article was the description of the depot following the injection and the tissue backpressure that results. Semi-mechanistic models were proposed to explain all the phases of the injection including the backflow resulting from the premature removal of the needle after injection. The prediction of maximum tissue backpressure, tissue relaxation and backflow are linked to one another since a higher pressure will lead to more rapid tissue relaxation and a reduced backflow. Overall prediction performance is adequate with average prediction errors ranging from 20 to 40%, i.e., much less than the observed variability in the measurements. However, there are important gaps which will need to be addressed in future versions. The main gap is to be able to predict tissue backpressure with highly viscous solutions or solutions which have non-Newtonian viscous behaviour. For example, this work could benefit from the multiphysics model introduced by Zheng *et al.* [63] and Hou *et al.* [64] to gain additional mechanistic insights into fluid flow through tissue as well as the impact of viscosity and mechanical interlocking of hyaluronan. This latter point will greatly benefit from a more mechanistic fluid flow model since the degradation rate of hyaluronan following infusion of formulations comprising rHuPH20 will dynamically alter the resistance to fluid flow. Other developments that could be of interest relate to the effective viscosity of injected formulations. For example, high-dose antisense oligonucleotide aqueous solutions are rheo-thinning and would need a specific treatment in the model, such as the use of the shear forces at the needle tip to predict actual viscosity at the point of injection. For viscous solutions, literature shows that the tissue backpressure is hard to measure, i.e., it does not add additional pressure compared to that needed to push the formulation through the syringe and needle. Doughty *et al.* found that the pressure related to the tissue backpressure could not be measured for a povidone solution of 13.6 mPa.s [47]. Allmendinger *et al.*, showed in live minipigs that from 10 mPa·s, and between 0.025 mL/s to 0.2 mL/s, the force needed to inject a solution subcutaneously is the same as the force needed to push the formulation through the injection device outside the animal body [48]. The reason behind these observations could be that the tissue mechanical resistance to the shear forces resulting from waterfront movement at high speed will be lower than the force or pressure needed to push the viscous formulation through the device, hence the inability to measure the tissue backpressure. The shapes of injected depots in the SC space are very similar regardless of the formulation viscosity [47, 48], which would indicate that the tissue mechanical resistance was overcome for both low and high viscosity fluids and that the tissue accommodated the formulation in the same apparent space. Allmendinger *et al.* nevertheless reported tissue back force values, which were transformed to tissue backpressure and compared to model predictions. If the backpressure prediction for a 100 mPa.s solution injected at 1.5 mL/min is correctly predicted by the model, when the injection rate increases for viscous solutions of 10 to 20 mPa.s, the model tends to overpredict the observed data (Fig. 11).

In the current model, the depot elongation and final shape depends only on the volume injected and injection rate. The current model is validated for low to high (1 to 100 mPa·s) viscosity formulations for low injection rates (below 2 mL/min). An apparent formulation and interstitial fluid viscosity is used in the backpressure calculation in the modified Darcy’s Eq. 19 using a fixed 10:90 formulation:IF ratio, which predicts an increase in tissue backpressure with formulation viscosity. This model is well verified at low injection rates however, it is likely that the tissue mechanical resistance will impose a cap to the calculated tissue backpressure. As for any material, the subcutaneous tissue will display a yield pressure where the stress strain relationship will not be linear anymore. Further studies will be needed with similar experimental settings as the work by Doughty *et al.* to refine the model for tissue backpressure. Another explanation for the apparent prediction errors at high injection rates is that the number of independent experiments is too low to allow for a meaningful quantification of the tissue backpressure.

In view of these observations, SubQ-Sim 2.0 can be used with high confidence to estimate the tissue backpressure for low to high viscosity solutions up to 2 mL/min injection rates and for low viscosity solutions up to 12 mL/min injection rates. Since the depot shape and RCS calculation are not impacted by formulation viscosity, SubQ-Sim can be used to determine the starting depot drug distribution in the subcutaneous tissue.

The formulation backflow is adequately predicted by the current model proposed in SubQ-sim. Being able to predict the impact of human factors virtually will help improve the robustness of injection devices and allow a better training of patients to avoid potential misuse of the device.

Finally, the successful prediction of the impact of *in situ* hyaluronan degradation offers the possibility to explore virtually the effect of hyaluronidase concentration on the tissue backpressure, in all patients with all needle configurations. This could reduce the number of animal or human studies needed to develop devices and formulations compatible with the administration of highly viscous solutions or large volumes to the subcutaneous space.

Overall, SubQ-Sim v2.0 provides important starting conditions for predicting drug absorption from the SC depot: depot shape, drug distribution, leakage from puncture hole and protein distribution. These conditions are needed to calculate over time the competition between drug protein binding and drug self-association as oligomers which will impact the diffusion through the extracellular matrix and the time needed to reach the systemic circulation [65].

In the next article, the drug absorption parameters with special regard to oligomerisation and protein binding will be discussed. The absorption pathways will be described, and the model validation will be presented. Many physiological parameters and “life events” are anticipated to modify the subcutaneous system physiology, which may influence the drug absorption rate and extent from the subcutaneous space. This justifies the development of a mechanistic physiologically based biopharmaceutics model to link device and formulation quality attributes to the drug product’s *in vivo* performance.

### Supplementary Information

ESM 1 (DOCX 11941 kb)
