# Supplementary material for: SubQ-Sim: A Subcutaneous Physiologically Based Biopharmaceutics Model. Part 1: The Injection and System Parameters
Source: Pharm Res. 2023 Aug 27;40(9):2195–214. doi: 10.1007/s11095-023-03567-0 (PMC10547635; doi:10.1007/s11095-023-03567-0)
Supplement: Supplementary file 1 — (DOCX 11941 kb) [file 11095_2023_3567_MOESM1_ESM.docx]

SubQ-Sim: A subcutaneous physiologically based biopharmaceutics model. Part 1: the injection and system parameters.

Supplementary materials

# System parameters for individualized modeling approach

Many parameters such as the distance between capillaries, the size of adipocytes and the blood flow to the adipose tissue, are influenced by anthropometric considerations even for a healthy population. Diseases like diabetes or obesity will also modulate physiological parameters relevant to the absorption of drugs from the subcutaneous tissue. Note that in the values below, when data was given in kg, the transformation to liters was achieved by dividing kg by the adipose tissue density of 0.92 kg/L.

## Body mass index and total body fat

The body mass index of a human (BMI) is defined below

$$\begin{aligned} BMI=\frac{Weight (kg)}{{Height (m)}^{2}}\#\left( SEQ Equation \backslash* ARABIC 1 \right) \end{aligned}$$

The total body fat is generally obtained by total body scan using a relevant label and is in the order of tens of litres. The relationship between body fat and BMI is illustrated in Figure 1.

Figure 1 Relationship between body fat (L) and BMI (kg.m-2). Data from pooled n= 3894 humans age 6-71.8. From [1-7]


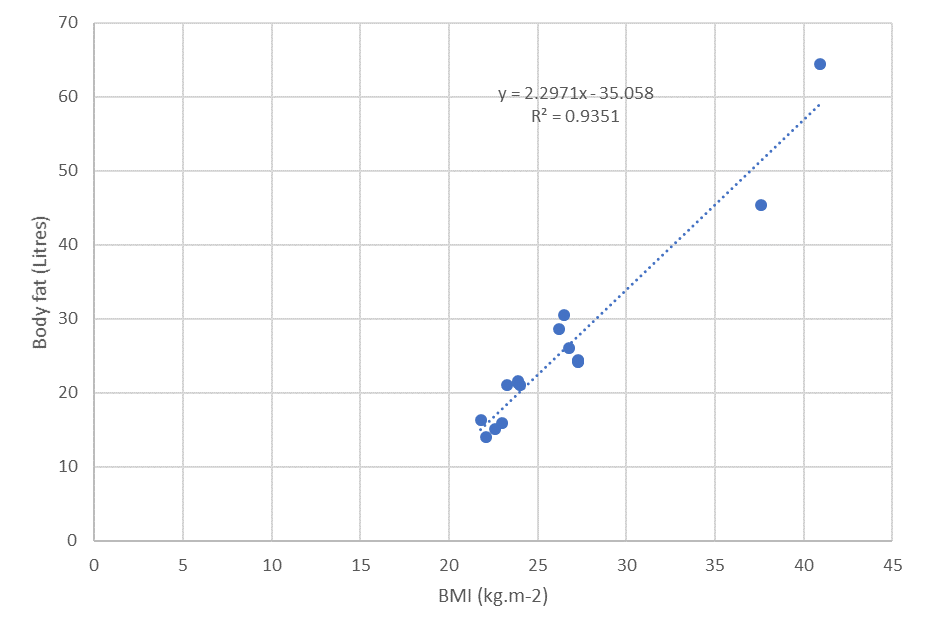


The total fat of the body is used in the calculation of the body fat percentage, by dividing the total fat mass by the body weight.

## Total body fat percentage

Gallagher et al. have established correlations to estimate the percentage body fat based on ethnicity, gender, age and BMI [8]. The following relationships can be used:

For white and African-American subjects, the percentage body mass is given by:

$$\begin{aligned} BF\left( \% \right)=64-\frac{848}{BMI}+0.079\times Age-16.4\times Sex+0.05\times Sex\times Age+39\times\frac{Sex}{BMI}\#\left( SEQ Equation \backslash* ARABIC 2 \right) \end{aligned}$$

Where sex = 1 for men and 0 for women

For Asian women, the relationship is:

$$\begin{aligned} BF\left( \% \right)=64.8-\frac{752}{BMI}+0.016\times Age\#\left( SEQ Equation \backslash* ARABIC 3 \right) \end{aligned}$$

For Asian men, it is:

$$\begin{aligned} BF\left( \% \right)=51.9-\frac{740}{BMI}+0.026\times Age\#\left( SEQ Equation \backslash* ARABIC 4 \right) \end{aligned}$$

## Adipocyte diameters

Tchoukalova et al. have reported the evolution of abdominal, gluteal and femoral subcutaneous adipocyte size as a function of total body fat percentage and gender [9]. The following relationships can be used for estimation of the adipocyte diameters (Figure 2, Figure 3, and Figure 4).

Figure 2 Abdominal adipocyte diameter versus body fat for men (left) and women (right). From [9]


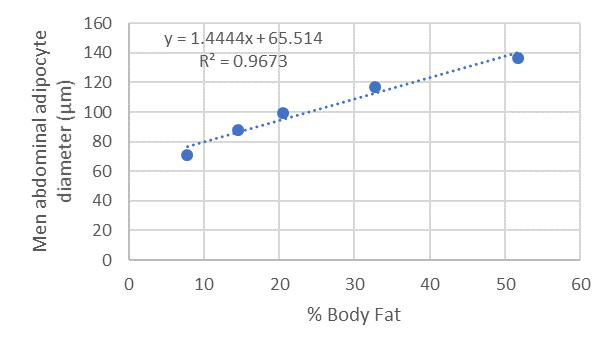

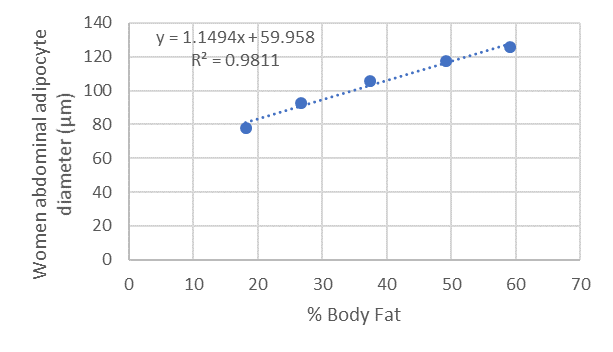


Figure 3 Gluteal adipocyte diameter versus body fat for humans. From [9]


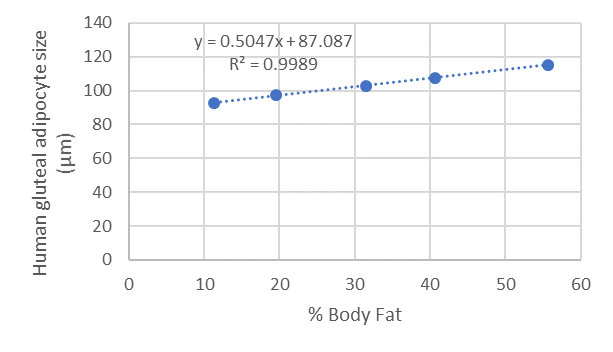


Figure 4 Femoral adipocyte diameter versus body fat for men (left) and women (right). From [9]


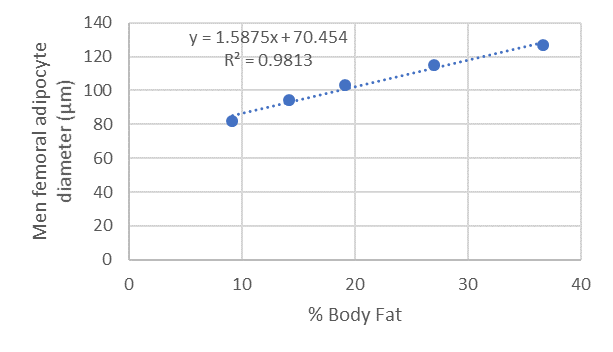

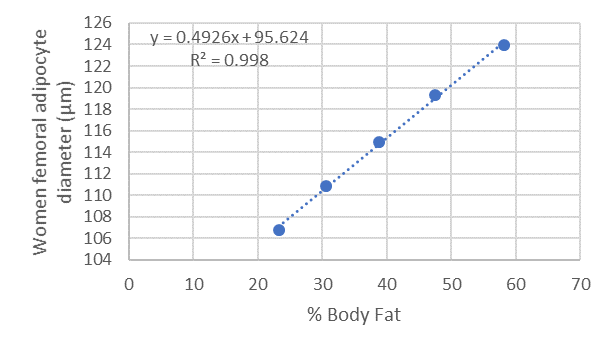


## Impact of BMI and sex on skin thickness and adipose tissue thickness

The skin thickness and adipose tissue thickness were measured by Jain et al. [10] in 101 type-2 diabetic insulin naïve adult patients. There are differences based on sex and BMI as illustrated below. Females have thinner skin whilst they have thicker adipose tissues for a given BMI. In this analysis, the skin thickness is that of the epidermis and dermis added.

Figure 5 Abdomen skin thickness as a function of BMI and sex


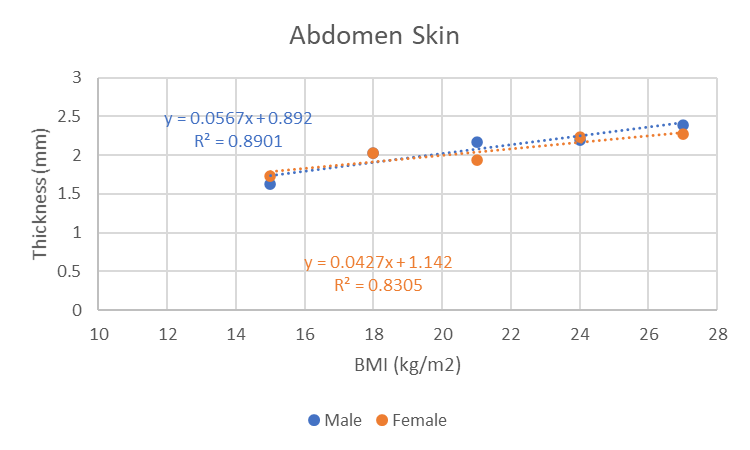


Figure 6 Thigh skin thickness as a function of sex and BMI


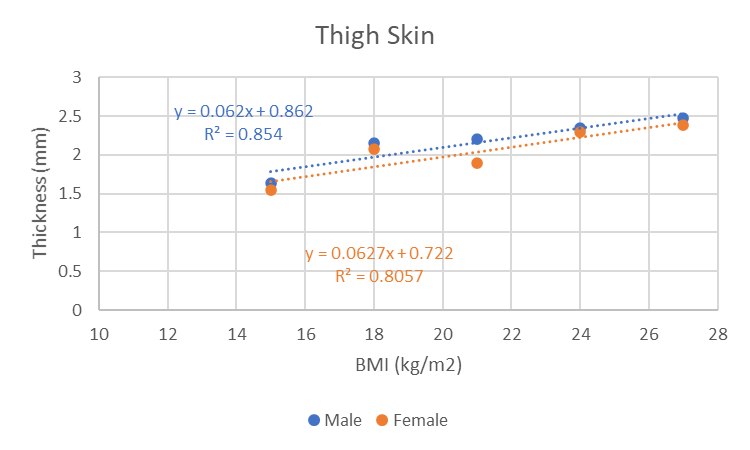


Figure 7 Arm skin thickness as a function of sex and BMI


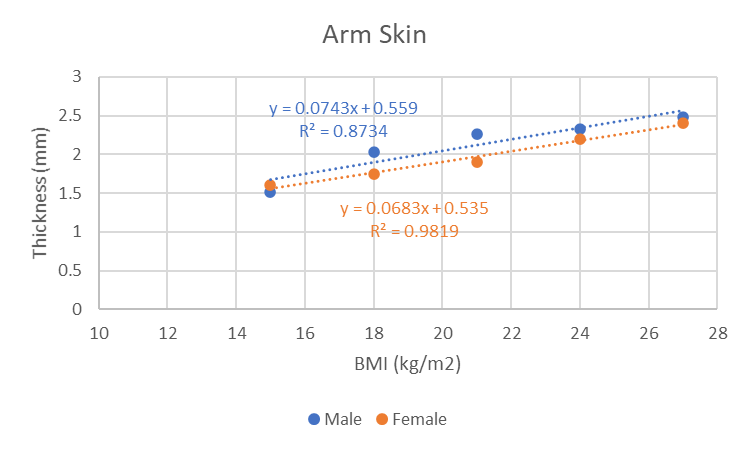


Figure 8 Abdomen SC tissue thickness as a function of BMI and sex


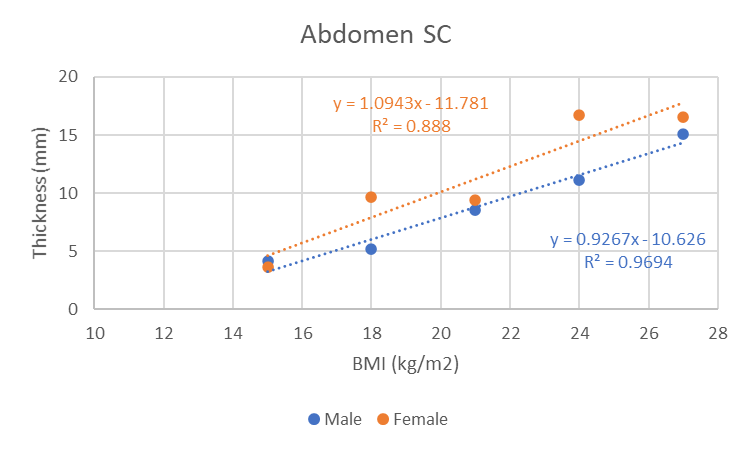


Figure 9 Thigh SC tissue thickness as a function of sex and BMI


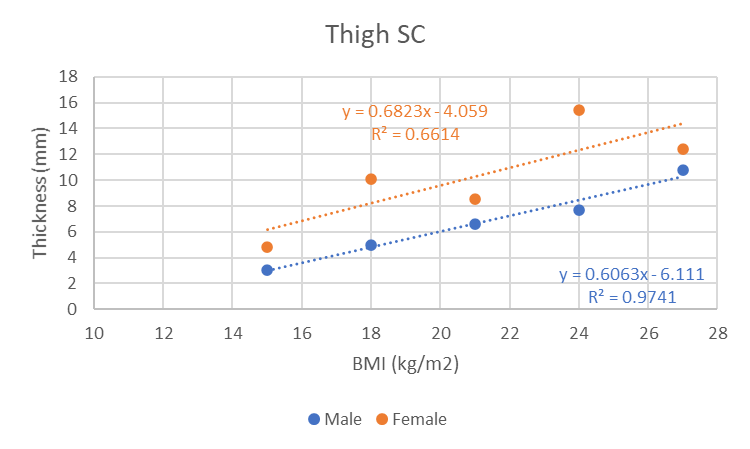


Figure 10 Arm SC tissue thickness as a function of sex and BMI


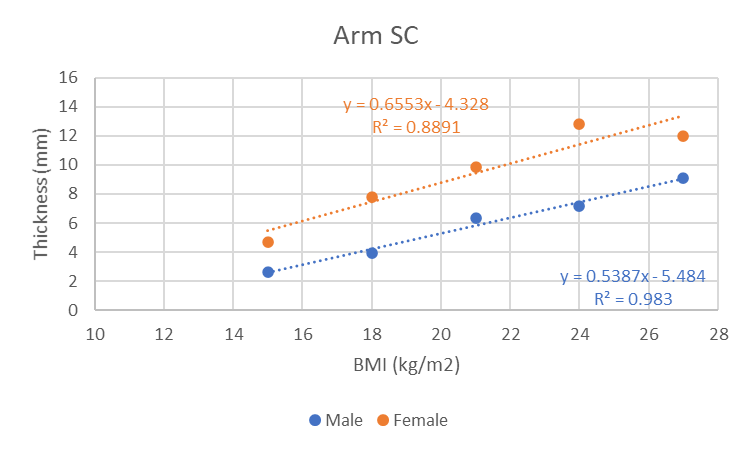


## Impact of injection site on skin elasticity and calculation of skin stiffness based on deformation angle

The skin elasticity was measured with Cutometers by various authors in human in different locations. Ezure et al. [11] have shown that there is a negative correlation between elasticity and thickness of the adipose tissue for facial measurements in women. Cutometers® (Courage & Khazaka, Cologne, Germany) apply a negative pressure on the skin over a given time with a hollow cylinder probe of given diameter. The skin deformation (skin raising in the cylinder) is measured with optical sensors and several parameters can be reported. U_f_, is the measure of the maximum skin deformation under pressure, which we can use to define a stiffness.

Figure 11 Schematic measurement principle of a Cutometer®


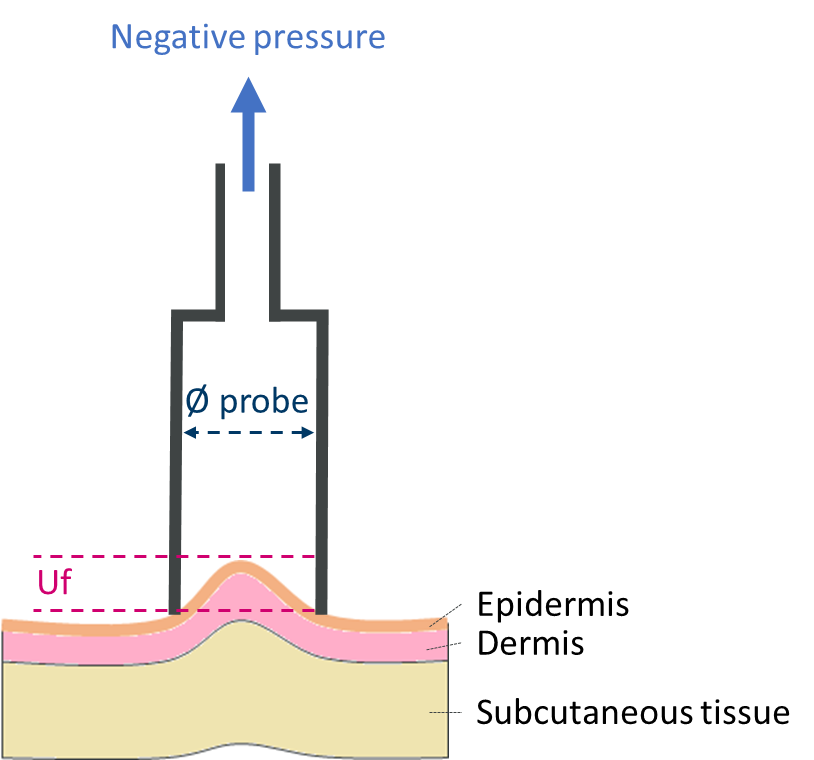


Figure 12 Typical skin deformation in a Cutometer®


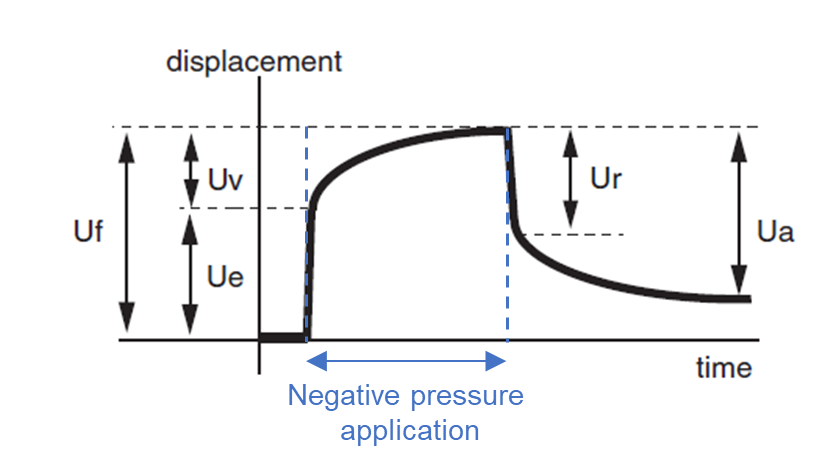


In order to analyze the Cutometer data, an angular skin deformation is calculated based on the values of U_f_ and probe diameter, assuming that the shape of the pinched skin surface is spherical (Figure 13).

Figure 13 Definition of angular skin deformation from Cutometer data


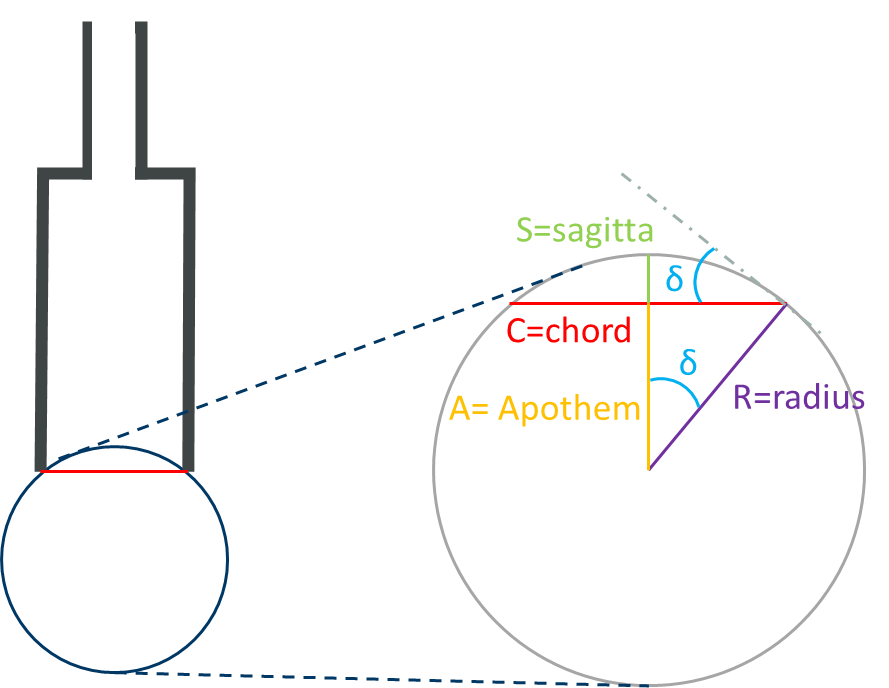


The chord (C) is equivalent to the probe diameter, the sagitta (S) defined by the chord is the U_f_ measurement from the Cutometer®. The radius of the circle defined by chord and sagitta (R), is simply given by:

$$\begin{aligned} R=\frac{S}{2}+\frac{C^{2}}{8S}\#( SEQ Equation \backslash* ARABIC 5) \end{aligned}$$

The Apothem (A) is given by:

$$\begin{aligned} A=R-S=R-U_{f}\#( SEQ Equation \backslash* ARABIC 6) \end{aligned}$$

And the deformation angle δ is given by:

$$\begin{aligned} \delta=acos\left( \frac{A}{R} \right)\#( SEQ Equation \backslash* ARABIC 7) \end{aligned}$$

The negative applied pressure is used to calculate the skin stiffness (St_skin_) with:

$$\begin{aligned} {St}_{skin}=\frac{\Delta P}{\delta}\#( SEQ Equation \backslash* ARABIC 8) \end{aligned}$$

Examples of measured skin stiffness are shown in Table 1.

Table 1 Measured skin stiffness from various body locations on five adult healthy japanese volunteers (2 males and 3 females). An 8mm probe applying 450 mBar negative pressure Cutometer® was used. From [12]

| Location | U_f_ (mm) | R values (mm) | Apothem (mm) | Deformation (degrees) | Skin stiffness (mBar/degree) |
| --- | --- | --- | --- | --- | --- |
| Forehead | 0.65 | 7.26 | 6.61 | 24.4 | 16.4 |
| Superior eyelid | 0.90 | 5.46 | 4.56 | 33.4 | 12.0 |
| Lower jaw | 0.98 | 5.06 | 4.08 | 36.3 | 11.0 |
| Ear lobe | 1.01 | 4.98 | 3.98 | 37.0 | 10.8 |
| Deltoid | 0.79 | 6.10 | 5.31 | 29.5 | 13.6 |
| Outside of upper arm | 0.85 | 5.73 | 4.89 | 31.5 | 12.7 |
| Medial side of upper arm | 1.10 | 4.65 | 3.55 | 40.2 | 9.9 |
| Palm | 0.55 | 8.47 | 7.92 | 20.7 | 19.3 |
| Scapular region | 0.77 | 6.24 | 5.47 | 28.7 | 13.9 |
| Anterior chest | 0.75 | 6.34 | 5.58 | 28.2 | 14.2 |
| Upper abdomen | 0.98 | 5.09 | 4.11 | 36.1 | 11.1 |
| Lateral abdomen | 0.99 | 5.05 | 4.06 | 36.5 | 11.0 |
| Lower abdomen | 0.90 | 5.44 | 4.53 | 33.5 | 11.9 |
| Lateral thigh | 0.73 | 6.50 | 5.76 | 27.5 | 14.5 |
| Anterior lower leg | 0.47 | 9.88 | 9.41 | 17.7 | 22.6 |
| Planta | 0.40 | 11.41 | 11.01 | 15.2 | 26.2 |

Table 1 shows that the skin stiffness of the abdomen is relatively constant and similar to measurements done on the lower jaw. However, the arm shows a level of stiffness which is approximately 15% higher than the abdomen, and the thigh has 32% higher stiffness compared to the abdomen. This higher stiffness for arm and thigh compared to abdomen cannot be related to skin thickness (Section 4) and therefore must be generated either by changes in composition of either the skin or the underlying tissues. A 2-fold higher backflow was measured in the thigh compared to the abdomen and the skin stiffness could contribute to a higher bleb pressure for given injection conditions.

To predict the skin thickness for any individual, the BMI, sex and injection site are used to calculate the thickness of the adipose tissue (Section 4). The thickness of the adipose tissue is then used to predict the stiffness of lower jaw skin using the data generated by Enzure et al. [11] (Figure 14).

Figure 14 Lower jaw skin thickness based on adipose tissue thickness measured using a 2 mm probe and 400 mBar negative pressure on a Coutometer®. From [11]


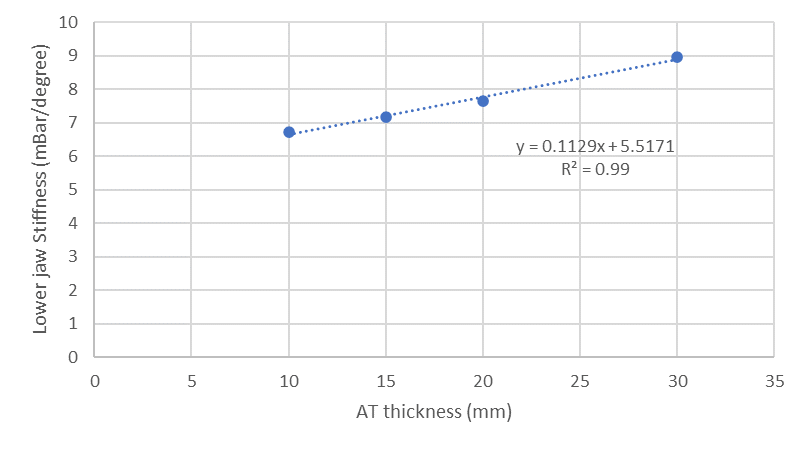


The resulting stiffness is multiplied by 1.03 to predict abdomen skin stiffness, by 1.152 to predict arm skin stiffness, and by 1.321 to predict thigh skin stiffness, according to the correlations established from Table 1.

The depot is assumed to deform the skin depending on the depth of injection. To calculate the angular deformation on the skin, two angles are calculated. The first one is (δ_i_), the angle made between a horizontal line through the centre of the depot and the line connecting the edge of the depot and the highest centre point of the bleb (Figure 15).

Figure 15 Position of the bleb following SC injection.


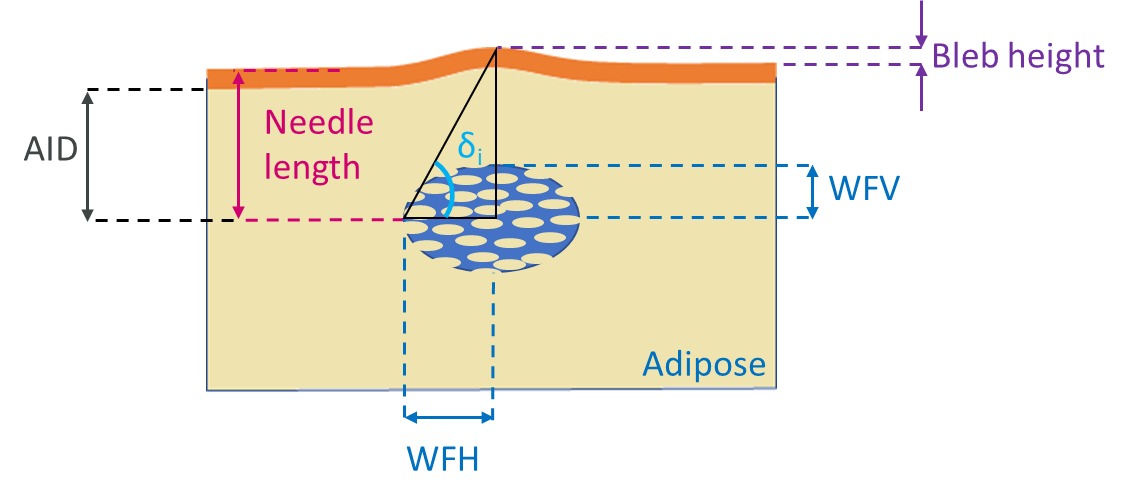


WFH = Waterfront Horizontal, WHV = Waterfront Vertical, AID = Adipose Injection Depth.

A second angle (δ_0_) is similarly calculated assuming that there was no bleb following injection. (Figure 16).

Figure 16 Angular deformation due to bleb following SC injection.


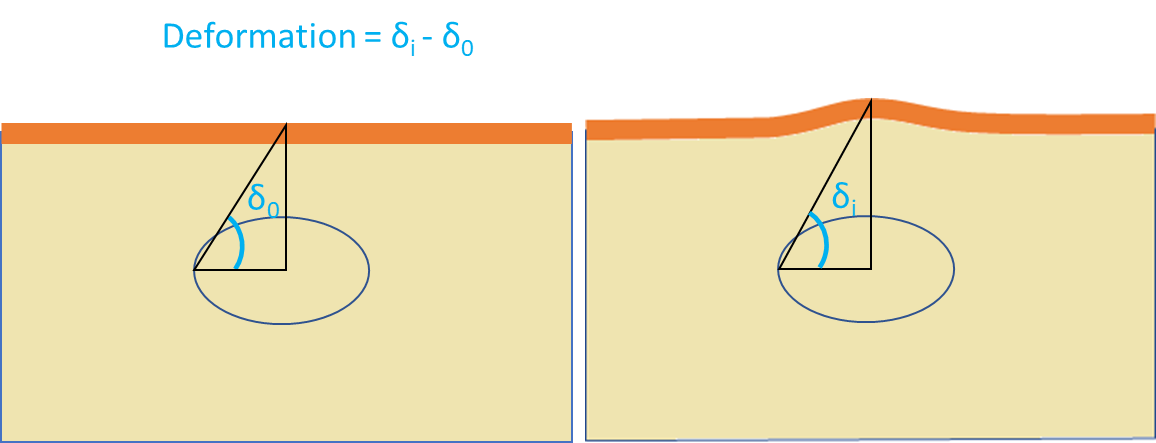


The final angular deformation is given by:

$$\begin{aligned} \delta=\delta_{i}-\delta_{0}\#\left( SEQ Equation \backslash* ARABIC 9 \right) \end{aligned}$$

$$\begin{aligned} \delta=\tan^{-1} \left( \frac{WFV\times\left( RCS-\phi_{IF} \right)^{\frac{1}{3}}+NL\times\sin\alpha}{WFH} \right)-\tan^{-1} \left( \frac{NL\times\sin\alpha}{WFH} \right)\#\left( SEQ Equation \backslash* ARABIC 10 \right) \end{aligned}$$

Where $NL$ is the needle length and $\alpha$ is the injection angle with the skin ($\alpha=\frac{\pi}{2}$for a perpendicular injection). The bleb residual pressure is then given by multiplying the angular deformation with the tissue stiffness as defined in Table 1.

Figure 17 Comparison between bleb residual pressure measurements and prediction for 2 mL saline injection in pigs at three injection rates from Doughty et al. [13]


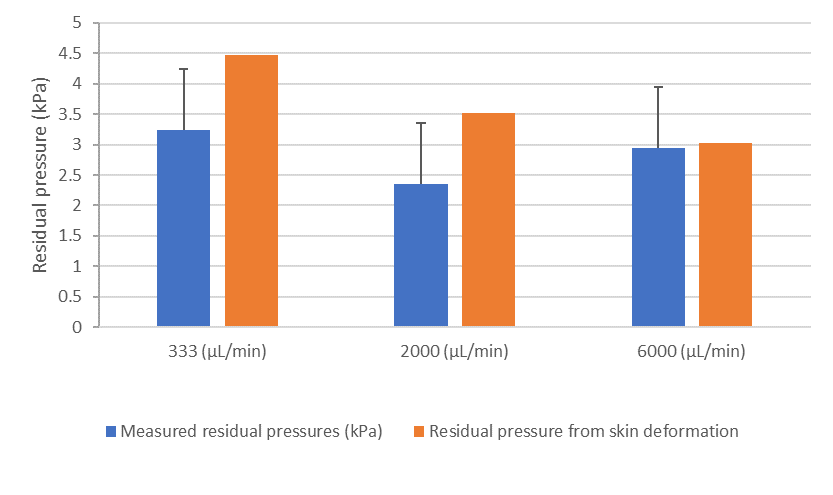


## Capillarity and distance between 2 capillaries

The number of capillaries per adipocyte (Cap) has been measured in various populations [14, 15]. It is proposed to calculate its value depending on the BMI. For values below a BMI of 24, a constant value of 0.65 capillary per adipocyte is used. Above this BMI, the capillarity is proposed to reduce linearly with BMI with the following relationship

$$\begin{aligned} {Cap}_{adipocyte}=1.0077-0.0155\times BMI\#\left( SEQ Equation \backslash* ARABIC 11 \right) \end{aligned}$$

The distance between two capillaries is then simply given by the diameter of the adipocyte (see previous section) divided by the capillarity.

## Typical distance between two lymph collectors

The lymph ducts drain to lymph collectors of larger diameter (500-1000 µm) where the velocity of lymph can be measured (See Section 13). It is assumed that the diffusion of lymph up to the lymph collectors will be the rate limiting step to reaching the lymph main circulation (lymph collectors). After the lymph collectors are reached, the lymph velocity will define the time needed to reach systemic circulation as a function of the injection sites and lymph velocity (See Section 13). Schacht et al have reported the distance to reach a lymph collector in the thigh at 6 mm [16]. Based on graphs and techniques reported by Suami et al. [17, 18], the distance to reach a lymph collector in the upper abdomen is estimated to be 7 mm, in the lower abdomen 15 mm and 10 mm in the arm.

## Typical surface area between capillaries and tissue

Knowing the volume of blood in the capillaries and assuming a 7 µm diameter for the capillaries, it is easy to estimate the surface area for exchange between adipose capillaries and interstitial space. Since the volume of a cylinder is given by $L\pi r^{2}$ and its surface area by $L\pi2r$, the surface to volume ratio of a cylinder is of 2/r. Hence the surface area for exchange (S_cap_) is given by

$$\begin{aligned} S_{cap}=\frac{2}{r_{cap}}\times\frac{V_{Blood}}{M_{adipose}}\times\frac{{PBlood}_{cap}}{100}\times\frac{{PCO}_{adipose}}{100}\#\left( SEQ Equation \backslash* ARAB\mathrm{IC}12 \right) \end{aligned}$$

S_cap_ is estimated at 7.7 [5.8-1.1] cm^2^/g tissue in males and females.

## Typical surface area between depot and tissue

The surface area of exchange between a depot and the surrounding tissue is expected to depend on the volume injected but also on the shape of the depot. Thomsen et al. [19].reported shapes of insulin depots following injections of 0.1, 0.4 and 0.7 mL in the neck of pigs using X-ray tomography and iodine marking. The pictures range from a dense spherical depot to tree like architecture with more tissue penetration and small canals comprising the liquid formulation stemming from the needle tip. The minimum exchange surface between the depot and the surrounding tissue at the time of injection would be the injected volume V_inj_ using a spherical morphology:

$$\begin{aligned} S_{min}=4\pi\left( \frac{3V_{inj}}{4\pi} \right)^{\frac{2}{3}}\#\left( SEQ Equation \backslash* ARABIC 13 \right) \end{aligned}$$

The maximal exchange surface between the depot and the surrounding tissue would be to assume that the injected liquid perfuses the tissue and replaces the interstitial fluid without disrupting the proteoglycan space. The exchange surface would be that area formed by the cells in the apparent volume that the injected formulation occupies. This apparent volume is simply given by the injected volume divided by the IF volume fraction.

With the adipocyte diameter (d_adipocyte_) and assuming spherical morphology, the maximal exchange surface can be calculated with:

$$\begin{aligned} S_{max}=V_{inj}\times\frac{\theta_{cell}}{\theta_{ISF}}\times\frac{6}{d_{adipocyte}}\#\left( SEQ Equation \backslash* ARABIC 14 \right) \end{aligned}$$

For a 1 mL injection, the surface of contact of the depot with the tissue spans from 4.8 cm^2^ for S_min_ to 5160 cm^2^ for S_max_ using 0.86 and 0.1 for cell and IF volume fraction respectively, and 100 μm as a mean adipocyte diameter [20, 21].

| Table 2 Adipocyte estimated size from Salans et al.[21] | | | |
| --- | --- | --- | --- |
| Type of subjects | Region of sampling | Lipid content  (µg lipid per cell) | Adipocyte diameter^A^ (µm) |
| Non-obese | Gluteal | 0.49 | 101 |
|  | Abdomen | 0.45 | 98 |
|  | Triceps | 0.37 | 92 |
| Obese | Gluteal | 0.93 | 125 |
|  | Abdomen | 0.89 | 123 |
|  | Triceps | 0.83 | 120 |

A: calculated from a lipid density of 0.92 g/mL assuming a cell spherical morphology

The values reported by Salans et al.[21] are close to those independently measured by Löfgren et al. [22] (Table 3).

Table 3 Adipocyte size in healthy and obese men and women. From [22]

| N | Age (years) | BMI | Adipocyte volume (pL) | Gender | Adipocyte diameter (µm) |
| --- | --- | --- | --- | --- | --- |
| 17 | 40 | 23.5 | 378 | Men | 90 |
| 25 | 37 | 22.5 | 449.8 | Women | 95 |
| 10 | 42 | 40 | 840.6 | Men | 117 |
| 13 | 39 | 44.9 | 911.2 | Women | 120 |

For obese subjects, the size of the adipocytes is larger (around 123 μm ) than for normal subjects and the max surface area for a 1 mL injection would then be 4195 cm^2^.

## Typical volume occupied by the depot

The min and max volumes occupied by the depot (including surrounding tissue) are provided by:

$$\begin{aligned} V_{min}=V_{inj}\#\left( SEQ Equation \backslash* ARABIC 15 \right) \end{aligned}$$

$$\begin{aligned} V_{max}=V_{inj}\times\frac{\theta_{cell}}{\theta_{ISF}}\#\left( SEQ Equation \backslash* ARABIC 16 \right) \end{aligned}$$

For a 1 mL injection the volume ranges from 1 mL to 10.1 mL assuming the fractional cell and IF volumes reported in the main manuscript. Experimentally, the injected fluid distends the tissue and the relative concentration of the solution in the depot is higher than the IF volume fraction (See main manuscript). An average relative concentration of the solution in the depot (RCS) of 0.2 with ranges from 0.1-0.3 can be measured from actual depot sizes and therefore a more correct estimation of the max depot volume would be:

$$\begin{aligned} V_{max}=\frac{V_{inj}}{RCS}\#\left( SEQ Equation \backslash* ARABIC 17 \right) \end{aligned}$$

Which would still bring out a max depot volume of 5 mL (range 3-10 mL) for an injection of 1 mL.

## Typical distance between capillaries

In adipose tissue outside of the formulation depot, assuming that the adipose volume can be represented by a cube of approximately L_adipose_ = 24 cm edge, then the number of capillaries in a cross section of that cube, n_cap_ is given by:

$$\begin{aligned} n_{cap}=\frac{V_{Blood}\times\frac{{PBlood}_{cap}}{100}\times\frac{{PCO}_{adipose}}{100}}{\pi\times L_{adipose}\times r_{cap}^{2}}\#\left( SEQ Equation \backslash* ARABIC 18 \right) \end{aligned}$$

The average distance between two capillaries is given by:

$$\begin{aligned} d_{2cap}=\left( \frac{L_{adipose}}{\sqrt{n_{cap}}}-{2r}_{cap} \right)\#\left( SEQ Equation \backslash* ARABIC 19 \right) \end{aligned}$$

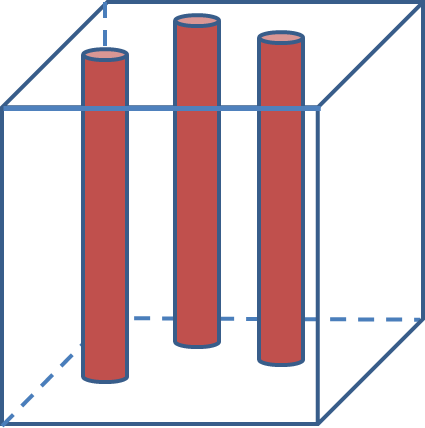


The radius for a capillary was taken at 3.5µm. For a healthy adult, the distance between capillaries is estimated at 188 µm [120-220] µm. Since in non-obese persons, the diameter of an adipocyte is estimated at 100 µm, it means that there is approximately 0.53 capillary per adipocyte, which corresponds to what was measured by Gealekman et al. [14]. Since the number of capillaries is reduced for obese patients, to 0.3 capillary per adipocyte and that the adipocyte diameter also increases to 125 µm, the estimated distance between two capillaries for obese patients is estimated to be of 417 µm. These parameters represent the distance to travel to reach a capillary in healthy or disease adipose tissue but inside the formulation depot, due to branching of formulation canals, this distance is anticipated to be smaller.

## Typical distance between two lymph ducts

Assuming that the adipose volume can be represented by a cube of approximately L_adipose_ = 24 cm edge. Then the number of lymph ducts in a cross section of that cube, n_duct_ is given by:

$$\begin{aligned} n_{duct}=\frac{\frac{M_{adipose}}{D_{adipose}}\times\theta_{Lymph}}{\pi\times L_{adipose}\times r_{duct}^{2}}\#\left( SEQ Equation \backslash* ARABIC 20 \right) \end{aligned}$$

The average distance between two capillaries is given by:

$$\begin{aligned} d_{2ducts}=\left( \frac{L_{adipose}}{\sqrt{n_{duct}}}-2r_{duct} \right)\#\left( SEQ Equation \backslash* ARABIC 21 \right) \end{aligned}$$

For a healthy adult, this distance is estimated at 312 µm, using a lymph duct diameter of 50 µm [23].

## Impact of posture and stature on lymphatic circulation

The posture and stature impact the venous and capillary pressure in the blood circulatory system. Levick et al. reported the effect of position relative to heart level upon vascular pressures in man. Capillary pressures were measured by micro-puncture of nailfold loops in hands and feet. Heart level was taken to be 7 cm below suprasternal notch [24]. Using the lengths of body segments relative to stature measured by Dempster et al. [25], the relative distance to the heart for various sites of the human body were computed in the standing, sitting and supine human body positions. These percent values can be multiplied by the body height to give the absolute distance from the heart. (Table 4).

Table 4 Percent distance to heart (normalized by stature). Positive values indicate positions below the heart and negative values above the heart.

| **Organ or site** | **Percent distance from heart (%)** | | |
| --- | --- | --- | --- |
|  | **Standing** | **Sitting** | **Supine** |
| Heart | 0 | 0 | 0 |
| Abdomen | 20.49 | 20.49 | 0 |
| Shoulder | -1.54 | -1.54 | 0 |
| Thigh (Inner) | 32.45 | 25.79 | 0 |
| Buttock | 25.79 | 25.79 | 0 |
| Thigh (Outer) | 30.23 | 25.79 | 0 |
| Upper arm | 6.66 | 6.66 | 0 |

Since Tansey et al. [26] reported pressure values according to body position, the distance from the heart was recalculated using Dempter et al. relative lengths and compared to those reported by Levick [24] (Figure 18). The agreement across both datasets is very good.

Figure 18 Hydrostatic pressure in veins as a function of distance from heart. From [24] and [26].


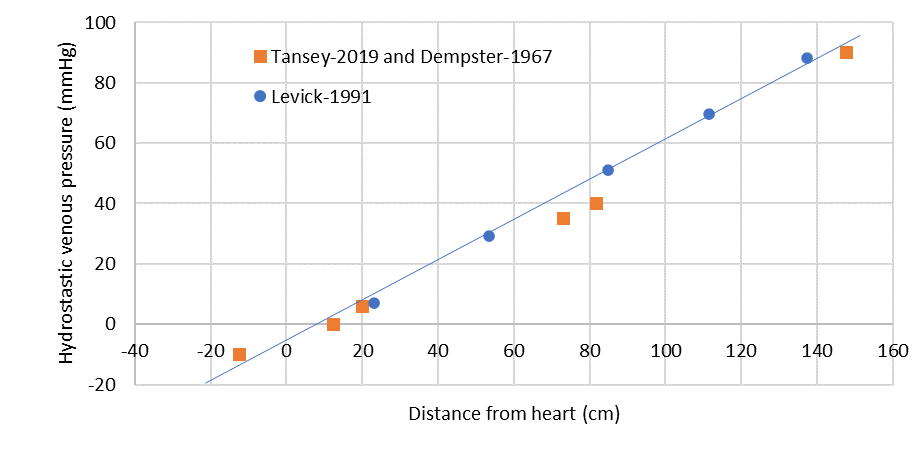


As a result, two relationships are used to calculate the pressure in the capillaries at the injection site due to pressure (Figure 19):

$$\begin{aligned} PV\left( mmHg \right)=7.2956+0.7075\times DMH(cm)\#\left( SEQ Equation \backslash* ARABIC 22 \right) \end{aligned}$$

Figure 19 Hydrostatic pressure in veins as a function of distance from heart. From [24]


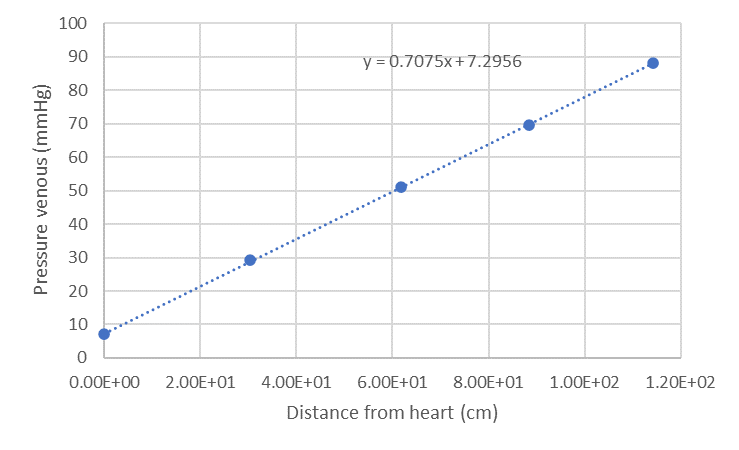


The pressure in the capillaries is calculated from the venous pressure using (Figure 20):

$$\begin{aligned} PC\left( mmHg \right)=25.664+0.7392\times PV\left( mmHg \right)\#\left( SEQ Equation \backslash* ARABIC 23 \right) \end{aligned}$$

Figure 20 Pressure in capillaries as a function of venous pressure in human. From [24]


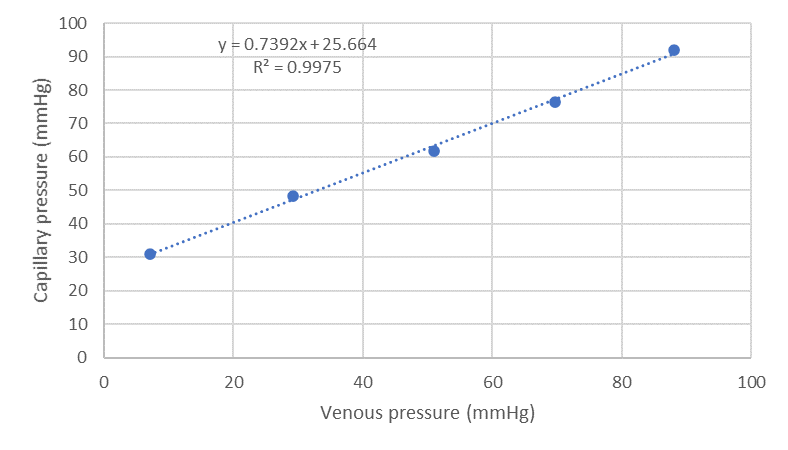


Uren et al. have measured lymph velocity in human as a function of the body site [27]. For each measuring site used by Uren et al. for lymph velocity, the capillary fluid pressure was computed using the above equations assuming a 1.72 m height individual. The capillary pressure is compared to measured lymph flow in Table 5 and Figure 21.

Table 5 Capillary pressure at the measuring sites of the human lymph velocity reported by Uren et al. [27]. Absolute distances computed for a 1.72 m human

| Measuring sites from Uren et al. [27] | Percent distance from heart (%) | distance from heart (cm) | Pressure venous (mmHg) | Pressure capillaries (mmHg) | Lymph velocity (cm/min) |
| --- | --- | --- | --- | --- | --- |
| Head and neck | -7.7 | -13.3 | -2.1 | 24.1 | 1.5 |
| Trunk | 5.6 | 9.55 | 14.0 | 36.0 | 3.35 |
| Arm and shoulder | -1.5 | -2.65 | 5.4 | 29.7 | 2 |
| forearm and hand | 22.9 | 39.41 | 35.2 | 51.7 | 5.5 |
| thigh | 25.8 | 44.36 | 38.7 | 54.3 | 4.2 |
| leg and foot | 71.7 | 123.38 | 94.6 | 95.6 | 10.2 |

Figure 21 Lymph velocity as function of capillary pressure


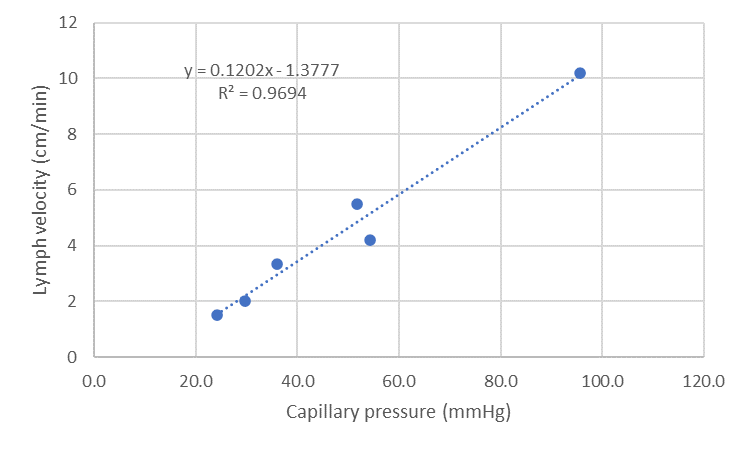


This relationship is expected from the mechanism of plasma filtration through capillaries [28-30]. A similar relationship was observed by Renkin et al. for the dog paw [31]. Assuming that the lymph collectors have 1 mm diameter [18], the lymph velocity reported in Figure 21 can be transformed into flow and compared to the values reported by Renkin (Figure 22). These values are comparable, showing that the filtration mechanism is similar.

Figure 22 Lymph flow for the dog paw and for human lymph collector as function of capillary pressure


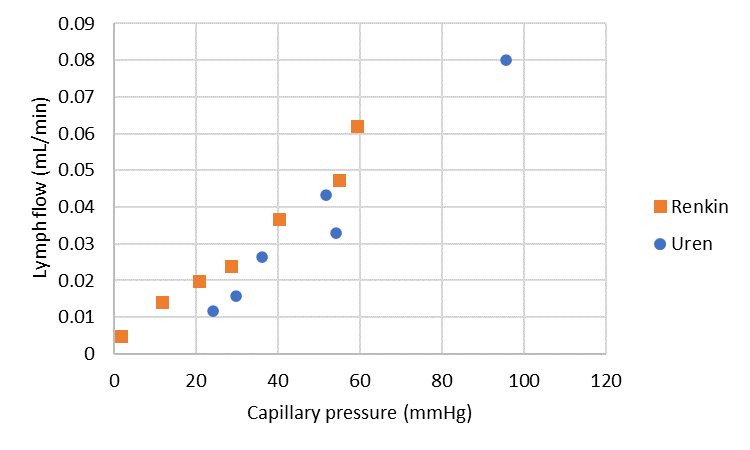


The lymph velocity can be used to calculate the time needed to reach the systemic circulation from the injection assuming that the drug travels through the lymphatic system. The percent distance relative to stature from the injection site to the thoracic duct were estimated using the values reported by Dempster et al. [25], and the lymph velocity was estimated using capillary pressure depending on the body position. Times to reach systemic circulation are reported in Table 6, and shown to range from 7-13 minutes in the sitting or standing position to 9-28 minutes in the supine position.

Table 6 Time to reach systemic circulation (SC) from the injection site via lymph. Absolute values for distance computed for a 1.72 m human.

| **Injection site** | **Distance to SC relative to stature (%)** | **Distance to SC calculated for 1.72 m human (cm)** | **Time to reach SC (min)** | | |
| --- | --- | --- | --- | --- | --- |
|  |  |  | **Standing** | **Sitting** | **Supine** |
| Abdomen | 25.8 | 44.3 | 10 | 10 | 19 |
| Shoulder | 12.9 | 22.2 | 10 | 10 | 9 |
| Thigh (Inner) | 37.7 | 64.9 | 11 | 13 | 28 |
| Buttock | 31.1 | 53.5 | 10 | 10 | 23 |
| Thigh (Outer) | 35.5 | 61.1 | 11 | 12 | 26 |
| Upper arm | 12.0 | 20.6 | 7 | 7 | 9 |

In parallel to estimating the time spent by the drug in the lymphatic circulation, the above relationships can be used to compute the ratio of lymph flow to venous flow as a function of capillary pressure. If one assumes that the lymph to venous flow equals 0.66 at the neck for an upright body position or a the heart level in supine position as reported by Levick et al. [24], then the ratio of lymph to IF flow can be computed for any position based on the capillary pressure (Figure 23).

Figure 23 Lymph flow divided by IF flow as function of capillary pressure


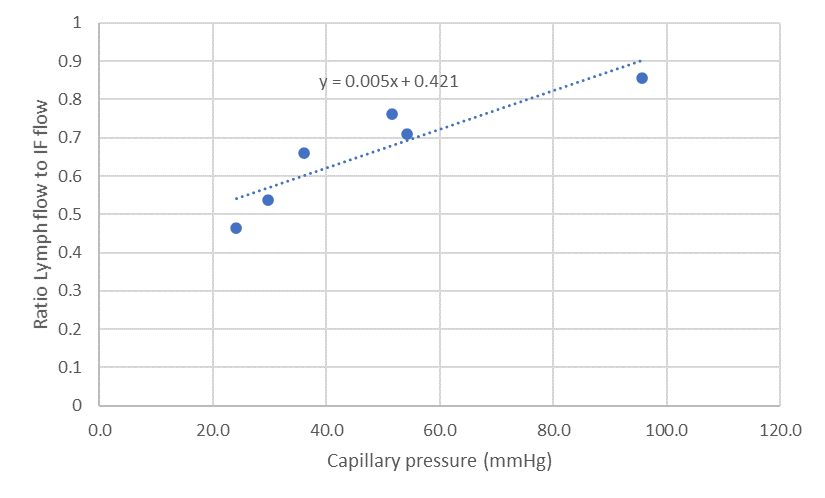

$$\begin{aligned} \frac{Q_{Lymph}}{Q_{IF}}=0.421+0.005\times PC\left( mmHg \right)\#\left( SEQ Equation \backslash* ARABIC 24 \right) \end{aligned}$$

## Temperature of the subcutaneous tissue

The temperature of the subcutaneous tissue was measured on the average at 33.9°C (for a skin temperature of 33.5°C) [32] The difference between skin and subcutaneous tissue was measured in various positions covering the human body [32-34] (Figure 24). The SC tissue is warmer than the skin in all conditions of internal (activity) or external applied temperature.

Figure 24 Relationship between skin temperature and subcutaneous temperature


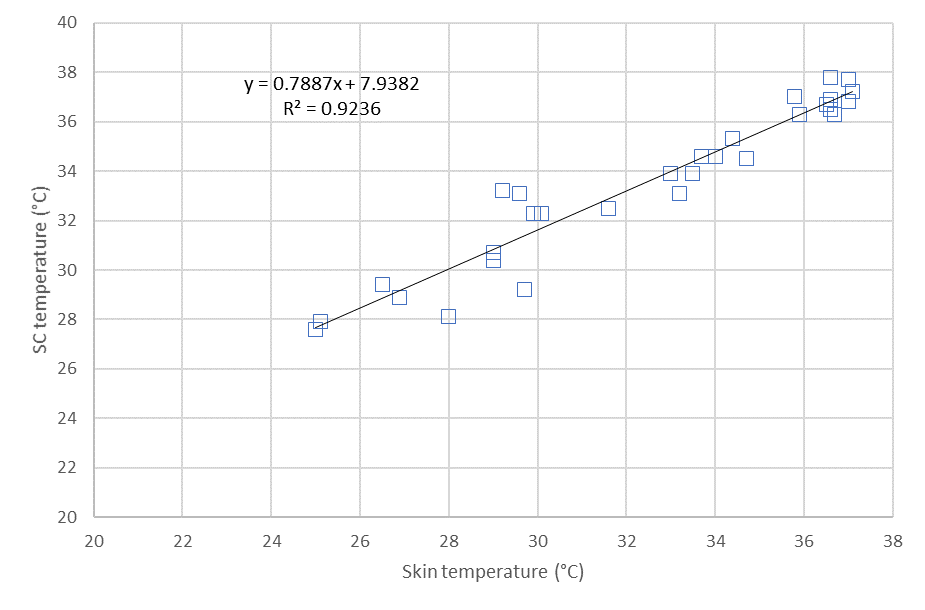


Since skin temperature in normal resting conditions in healthy subjects at room temperature was measured in different parts of the body [32], the corresponding SC temperature can be calculated from the equation presented in Figure 24.

Table 7 Skin temperature reported in normal subjects [32], and calculated SC temperatures (°C). Most frequent SC injection sites are highlighted in blue

| Body position | Skin temp (°C) | SC temperature (°C) |
| --- | --- | --- |
| Toe (volar) | 22.3 | 25.5 |
| Foot (volar,heel) | 25.3 | 27.9 |
| Foot (volar) | 26.3 | 28.7 |
| Foot (dorsal) | 28.6 | 30.5 |
| Finger (volar) | 28.8 | 30.7 |
| Knee | 29.1 | 30.9 |
| Finger (dorsal) | 29.5 | 31.2 |
| Hand (dorsal) | 29.8 | 31.4 |
| Thigh (anterior) | 30.3 | 31.9 |
| Hand (volar) | 30.4 | 31.9 |
| Forearm (dorsal) | 30.5 | 32.0 |
| Upper arm (posterior) | 30.5 | 32.0 |
| Thigh (lateral) | 30.7 | 32.2 |
| Thigh (posterior) | 30.8 | 32.2 |
| Leg (posterior) | 30.8 | 32.2 |
| Upper arm (lateral) | 30.9 | 32.3 |
| Leg (anterior) | 31.2 | 32.6 |
| Leg (lateral) | 31.3 | 32.6 |
| Forearm (ventral) | 31.5 | 32.8 |
| Upper arm (anterior) | 31.7 | 33.0 |
| Back (lumbar) | 31.8 | 33.0 |
| Abdomen | 32.1 | 33.3 |
| Posterior torso | 32.2 | 33.3 |
| Anterior torso | 32.2 | 33.3 |
| Chest | 32.3 | 33.4 |
| Back (scapular) | 32.7 | 33.7 |
| Head (cheek) | 32.7 | 33.7 |
| Neck (posterior) | 33.2 | 34.1 |
| Head (forehead | 34.1 | 34.8 |

In normal room temperature conditions, the abdomen SC temperature is about 1°C higher than the thigh or upper arm. In stressful external temperatures (0-50°C), the skin temperature was measured in humans and the adipose temperature can be calculated from these external measurements, using the relationship shown in Figure 24. The relationship between adipose tissue temperature and externally applied temperature is shown in Figure 25. There is a slight impact of exercise on top of external applied temperature to the skin (and adipose temperature)

Figure 25 Adipose tissue temperature as a function of externally applied temperature. Data from [33, 35]


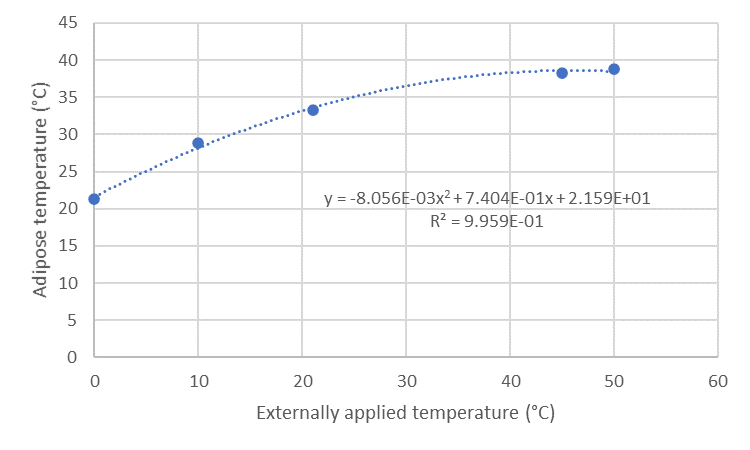


In conclusion, in most conditions (normal external temperature and no exercise), the SC temperature is about 34°C for the abdomen and 33°C for thigh and arm.

## Blood flow through the adipose tissue

To compute the blood flow through the adipose tissue and its contribution to IF flow, the following approach was taken. The basal cardiac output in the supine position is given by:

$$\begin{aligned} {CO}_{supine, basal}(L/min)=0.03469\times BW(kg)+2.291\#\left( SEQ Equation \backslash* ARABIC 25 \right) \end{aligned}$$

Figure 26 Cardiac output as function of body weight. From [36]


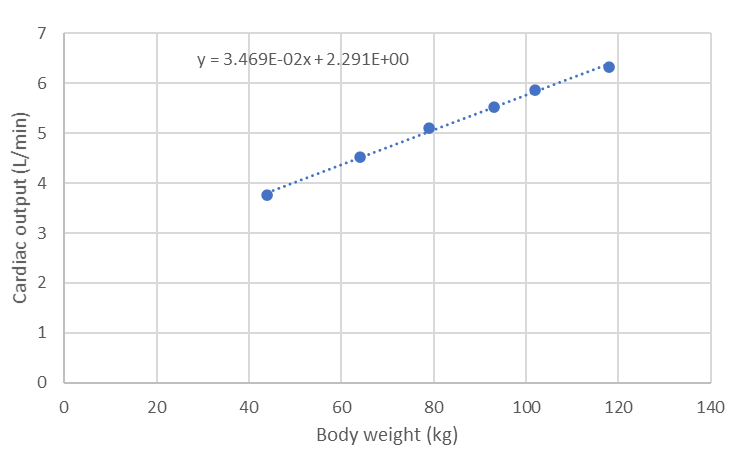


The blood volume is taken as 7.15% of the body weight [37].

The impact of external temperature on cardiac output was calculated from the data reported by Rowell et al. [35] (Figure 27).

$$\begin{aligned} {CO}_{supine, T℃}(L/min)=\left[ \left( T-22 \right)\times0.0071+1 \right]\times{CO}_{supine, basal}(L/min)\#\left( SEQ Equation \backslash* ARABIC 26 \right) \end{aligned}$$

Figure 27 Cardiac output fold increase as function of external body temperature variation. From [35]


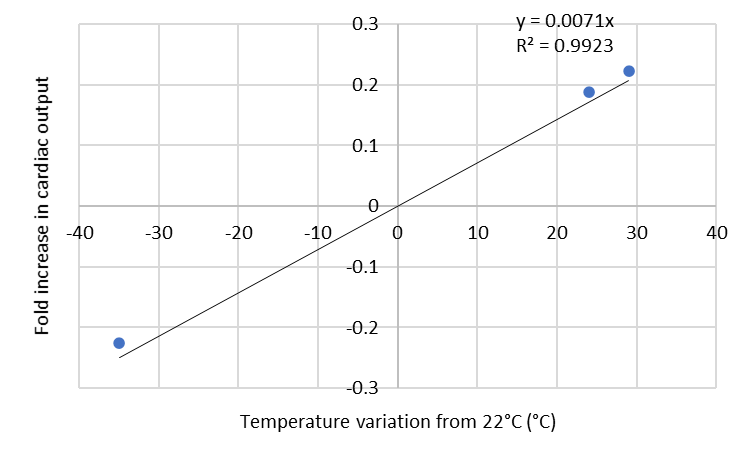


The impact of exercise on blood flow is taken from Flamm et al. [38]

Figure 28 Cardiac output fold increase as function of physical exercise. From [38]


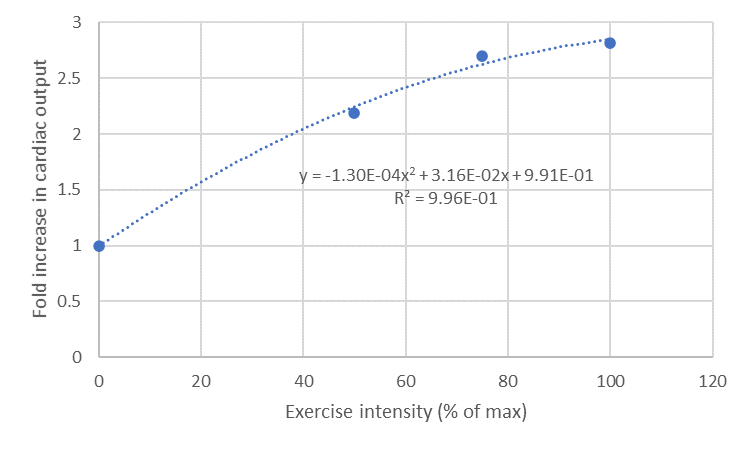


The added effect of body position on the cardiac output was to multiply the cardiac output by 1.2 if the body is not in supine position.

All the above parameters (effect of temperature, position and exercise) are considered to be multiplicative of one another, and lead to the definition of the basal cardiac output for a healthy individual.

The partial cardiac output to the adipose tissue in the abdomen, legs or arms and buttocks are given in Table 8 with literature references.

Table 8 Human partial cardiac output to the adipose tissue.

| Average pCO to SC tissues (%CO per kg of adipose tissue) | Value | Literature reference |
| --- | --- | --- |
| Abdomen | 0.664 | [39] |
| Legs or arms | 0.304 | [39] |
| Buttock | 0.221 | [40] |

These partial cardiac outputs are used to calculate the basal healthy adipose tissue blood perfusion (ATBF) according to the following equation

$$\begin{aligned} ATBF\left( \frac{\frac{mL}{min}}{100g} \right)=\frac{\frac{pCO}{100} {\times\frac{BF\left( \% \right)}{100}\times BW\left( kg \right)\times CO}_{supine, T℃}(L/min)\times1000}{\frac{BF\left( \% \right)}{100}\times BW\left( kg \right)\times1000}\times100\times1.5\#\left( SEQ Equation \backslash* ARABIC 27 \right) \end{aligned}$$

Which simplifies to

$$\begin{aligned} ATBF\left( mL/min/100g \right)=pCO (\%CO/kg tissue){\times CO}_{supine, T℃}(L/min)\times1.5\#\left( SEQ Equation \backslash* ARABIC 28 \right) \end{aligned}$$

The factor of 1.5 is added to scale the results to reference measurements made by Astrup et al. [33]. Up to now the values for ATBF are still concerning healthy volunteers and do not incorporate the effect of externally applied temperature, mental stress, sleep or food on the blood flow.

## Impact of temperature on ATBF

The variation in abdominal SC blood flow as a function of skin temperature in 12 healthy young humans is shown in Figure 29.

Figure 29 Variation in abdominal SC blood flow as a function of skin temperature in 12 healthy young humans from [33]


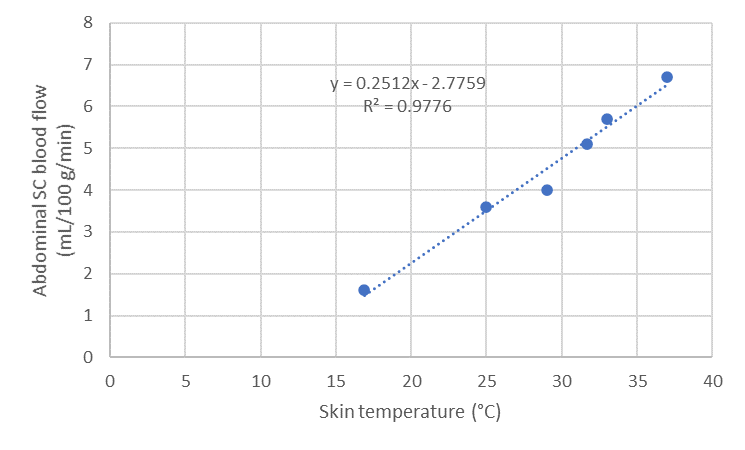


The impact of temperature on ATBF is adapted from the data presented by Astrup [33] by recalculating the fold increase in ATBF as a function of SC tissue temperature using a reference value of 5.7 mL/min/100 g for abdominal ATBF at a reference SC tissue temperature of 34°C. The updated relationship is illustrated in Figure 30.

Figure 30 Increase in abdominal ATBF as a function of tissue temperature variation. Data recalculated from [33]


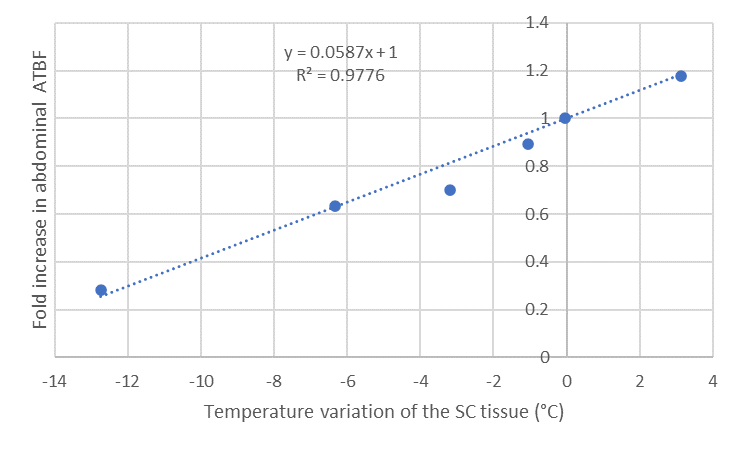


The temperature of the adipose tissue is itself a function of the external environmental temperature (Figure 31) and the extent of physical exercise (Figure 32).

Figure 31 Relationship between adipose tissue temperature and environmental temperature. Data recalculated from [33, 35]


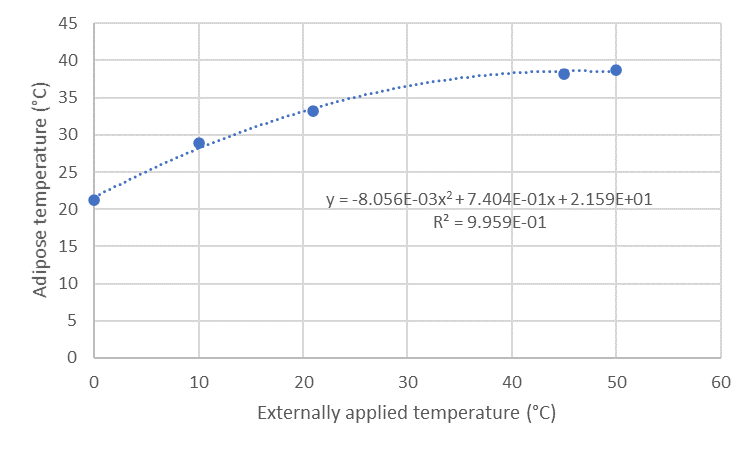


Figure 32 Relationship between adipose tissue temperature with and without exercise. Data from [35]


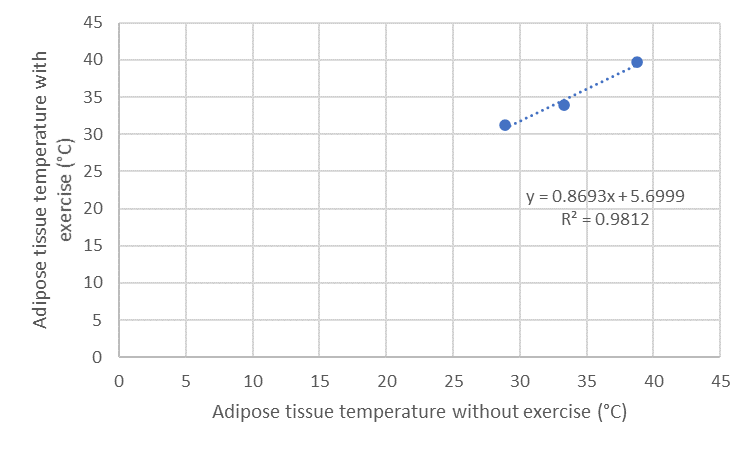


## Impact of mental stress on ATBF

As mentioned previously, the ATBF is multiplied by 1.89 for the legs, arms and buttock and by 1.63 for the abdomen in the presence of mental stress [41].

## Impact of food on ATBF

Based on the observation that meals increase the adipose tissue blood flow in healthy volunteers but not in obese subjects [42, 43], the following relationship is derived for healthy subjects.

Figure 33 Fold increase in abdominal ATBF due to food intake for healthy subjects. Data from [42, 43].


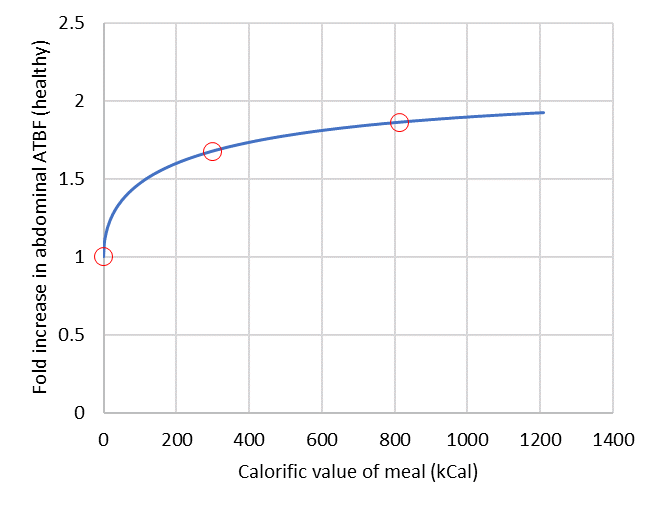
$\begin{aligned} Fold increase ATBF=1+1.06208\times\left( 1-e^{\left( -0.05863\times\sqrt{Cal} \right)} \right)\#\left( SEQ Equation \backslash* ARABIC 29 \right) \end{aligned}$

Where Cal is the food calorific value in kCal.

## Impact of disease on ATBF

Obesity was found to impact capillarity but the blood flow of obese patients does not react to temperature variation, food or stress as much as that of healthy subjects. As a consequence, the increase to the blood flow related to a temperature increase is limited in diabetic compared to healthy subjects (Figure 34).

Figure 34 Increase in blood flow related to external temperature in healthy subjects and diabetic patients per sampling regions.


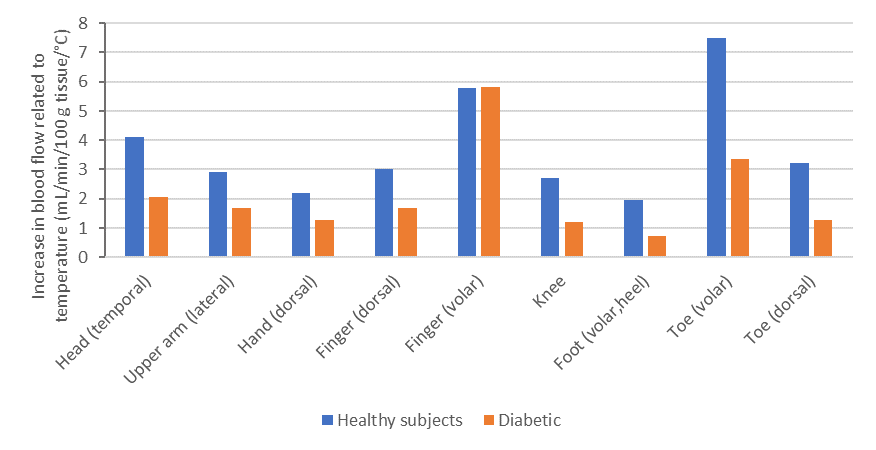


Therefore, it is proposed to use capillarity by mm^2^ cross surface area of tissue to scale the blood flow from a healthy to a diseased population, since the number of capillary lumens are a good representation of the flow rate in the tissue of interest. This capillarity is calculated from adipocyte size and capillarity by adipocyte using the following equation, where $D_{adipocyte}$ is the diameter of the adipocyte in mm in the given tissue (See Section 3) and where ${Cap}_{adipocyte}$ is the capillarity per adipocyte (See Section 6).

$$\begin{aligned} {Cap}_{surf} \left( \frac{Lumen}{{mm}^{2}} \right)=\frac{{Cap}_{adipocyte}}{\pi\left( \frac{D_{adipocyte}}{2} \right)^{2}}\#\left( SEQ Equation \backslash* A\mathrm{RABIC}30 \right) \end{aligned}$$

Using the above equations and anthropometric considerations, a reference value of 108.8 capillaries per mm^2^ of abdominal tissue is calculated (22 year old 1.8 m tall white male of 73 kg). Since obesity is already factored into the calculation of capillarity, the impacts (fold increase in ATBF) of external temperature, food and mental stress are scaled using the patient ${Cap}_{surf}$ divided by the reference value of 108.8 capillaries per mm^2^. For the gluteal area a reference capillarity of 92.3 capillaries per mm^2^ is calculated. For legs, arms and thighs a reference capillarity of 93 capillaries per mm^2^ is calculated.

## Impact of blood flow on pressure and liquid IF flow

The veins and capillaries are not rigid and the blood flow through these vessels does not translate into exerted pressure since the vessels have some capacitance, i.e. they can dilate and accommodate a higher blood volume thereby reducing the exerted pressure.

The flow through the capillaries is driven by the balance between peripheral vein pressure (PVP), central venous pressure (CVP) and the venous resistance (VenR) according to the following equation [44]:

$$\begin{aligned} Q_{Blood}=\frac{PVP-CVP}{VenR}\#\left( SEQ Equation \backslash* ARABIC 31 \right) \end{aligned}$$

The MCFP (Peripheral vein pressure in the absence of flow) in humans is around 8.6 mmHg [45] and the central venous pressure is on the average 3 mmHg [26]. A vascular resistance for the capillaries was measured at 0.22 mmHg/(mL/min/100g) by Johnson [46]. A similar value of 0.188 mmHg/(mL/min/100g) is found from the data of Guyton [47] by normalizing the resistance data with whole dog body (10 kg). Therefore the vein pressure related to flow can be computed from the previous equation.

The fluid flux across a capillary is drive by the following equation [30, 48]:

$$\begin{aligned} J_{V}=A\times L_{p}\times\left( \Delta P-\sigma\Delta\pi\right)\#( SEQ Equation \backslash* ARABIC 32) \end{aligned}$$

The hydrostatic pressure in the respective tissues is made from the addition of hydrostatic pressure and flow related pressure as detailed above and will depend on life events and activity level. First the total vein pressure is calculated by summing contributions of MCFP, hydrostatic and flow related pressure. The capillary pressure is then calculated from venous pressure using the relationship shown in Figure 20.

The interstitial hydrostatic pressure is taken at -2.1 mmHg [30], the plasma oncotic pressure is taken for human subcutaneous tissue at 26.8 mmHg [24] and the interstitial oncotic pressure is taken at 15.6 mmHg for the abdomen, 9.1 mmHg for the thigh and arm and buttock [24]. The reflection coefficient for albumin is taken at 0.85 [24]. Capillary hydraulic conductivities vary widely across experimentalists and tissues.

Table 9 Reported hydraulic conductivities in the literature

| Species | Tissue | Temperature | Lp value (mm/min/mmHg) | Ref |
| --- | --- | --- | --- | --- |
| Frog | Mesentery | 15 | 6.12E-04 | [49] |
| Frog | Mesentery | 15 | 5.38E-04 | [50] |
| Frog | Mesentery | 25 | 1.17E-03 | [51] |
| Frog | Mesentery | 8 | 7.50E-04 | [51] |
| Frog | Mesentery | 15 | 1.42E-04 | [29] |
| Frog | Mesentery | 32 | 1.30E-03 | [29] |
| Mammal | Skeletal muscle | NR | 1.93E-05 | [29] |
| Dog | Lung | NR | 2.69E-05 | [29] |
| Frog | Muscle | NR | 4.54E-05 | [29] |
| Rabbit | Heart muscle | NR | 7.37E-05 | [29] |
| Frog | Mesentery | NR | 3.26E-05 | [29] |
| Frog | Mesentery | NR | 2.22E-04 | [29] |
| Frog | Mesentery | NR | 3.83E-04 | [29] |
| Cat | Salivary gland | NR | 4.83E-04 | [29] |

The values used by Maeda et al. [52] of 1.54E-6 mm/min/mmHg were one log below those measured in the literature and were discarded. The value of hydraulic conductivity reported for mammalian muscle was used in the model (1.93E-05 mm/min/mmHg). The impact of tissue temperature on hydraulic conductivity was also included by using the following relationship (Figure 35)

Figure 35 Fold increase in hydraulic conductivity as a function of temperature with ref at 32°C. Data from [29, 51].


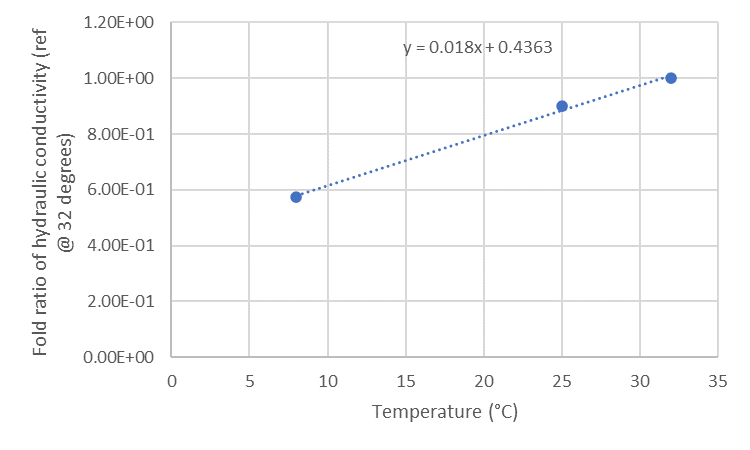


The surface area of the capillaries is calculated for each voxel (volume element) of the model by multiplying the ${Cap}_{surf}$ by the surface area of a voxel (giving the # of capillaries per voxel) and then multiplying this by the surface of one capillary. The liquid flux obtained by Equation $32$ corresponds to the interstitial fluid flux (Q_IF_) and will drive the extravasation of solutes from the plasma. The lymph return flow (Q_Lymph_) is given by multiplying the IF flow by the Lymph to IF ratio calculated based on capillary pressure (Figure 23). The venous return flow (Q_Venous_) is given by the difference between Q_IF_ and Q_Lymph_.

## Impact of disease on peptidase expression

Adipokine dipeptidyl peptidase-4 (DPP4) is a transmembrane glycoprotein and exoprotease that cleaves N-terminal dipeptides from various substrates. Most importantly, DPP4 also cleaves and inactivates the incretins glucagon-like peptide 1 (GLP-1) and gastric inhibitory polypeptide [53].

Figure 36 Expression of DPP-4 as function of BMI. Data from [53].


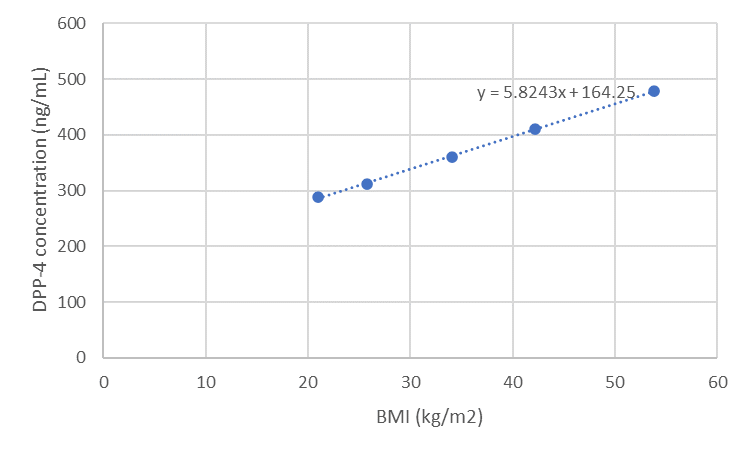


Neprilysin, is a zinc-dependent membrane metallopeptidase located on the cell surface, which inactivates several bioactive peptides, such as natriuretic peptides (NPs). Neprilysin is mainly synthesized in adipose tissue and can be separated from the cell surface to circulate in a soluble, catalytic active form in the blood stream. Pharmacological inhibition of Neprilysin leads to enhanced diuresis and natriuresis, likely because the degradation of NPs is inhibited [54].

Figure 37 Plasma concentration of Neprilysin as function of BMI. Data from [54].


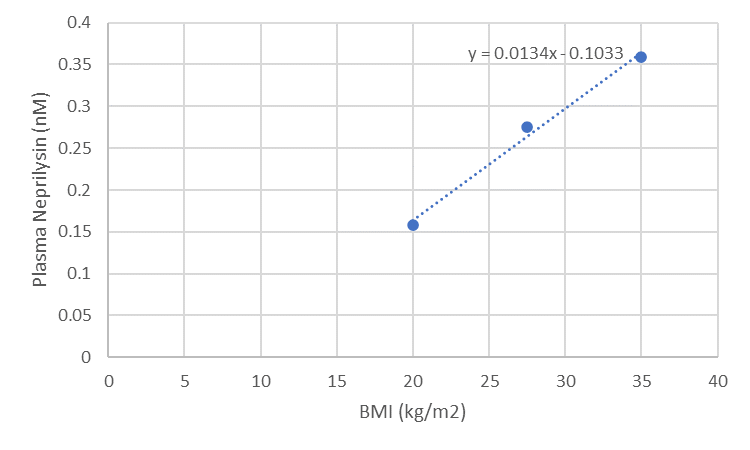


# Representation of the depot in a voxel grid

In order to accommodate the simulation of non-spherical depots, the injection site and the surrounding tissue are represented in the software by a 3-dimensional array of cubic voxels, typically 50 x 50 x 50. As described previously, (ref) the depot can either be spherical in shape or take the form of an oblate spheroid, where the extent of the depot in the direction of the vertical axis perpendicular to the skin surface is lower than in the horizontal direction.

Figure 38 below shows an example of a 30 x 30 x 30 voxel array, in which the shaded voxels are considered to be within the depot and therefore will contain an amount of API immediately after dosing (prior to the start of the simulation).

Figure 38 Example 3-Dimension Depot as Voxel Array (30 x 30 x 30). Here the total number of voxels is 27000 (left panel), however, in this particular case, only 663 (right panel) are initially considered to be part of the depot


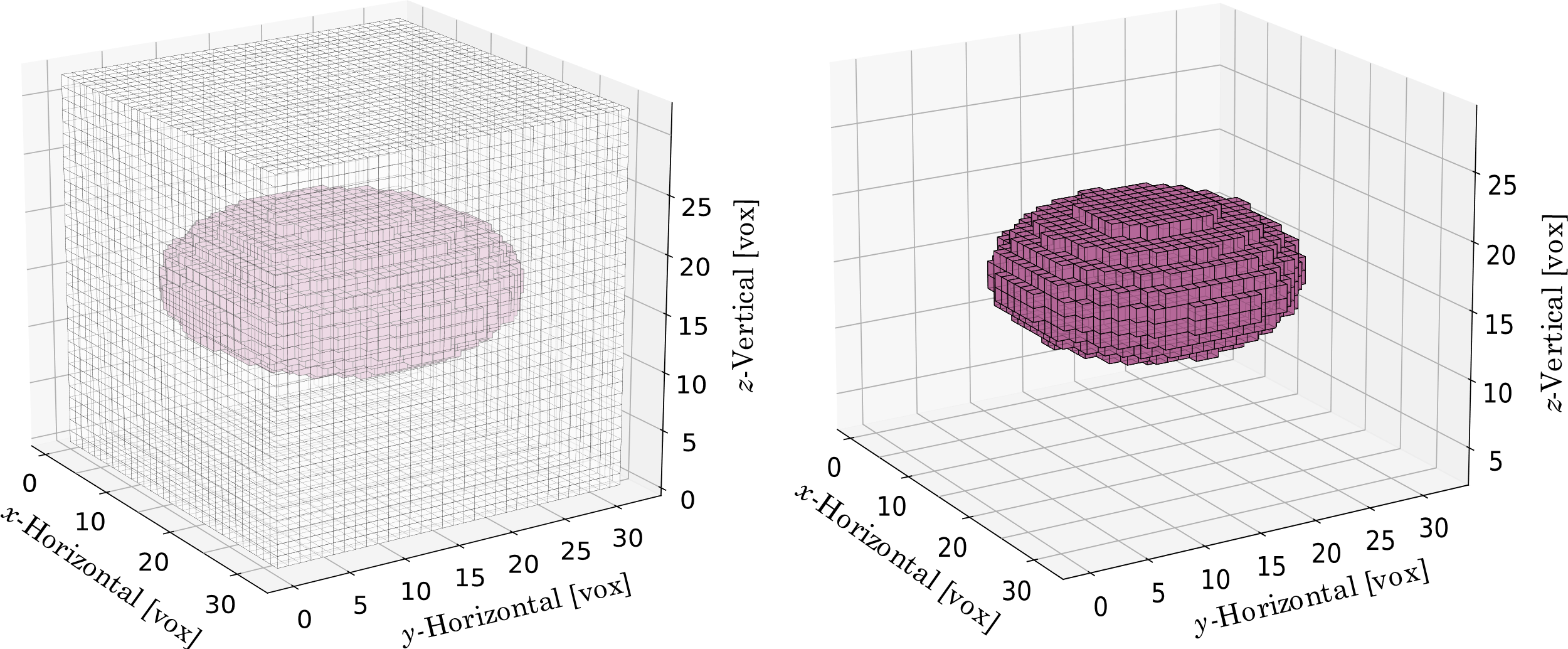


We have used an approach based on the work of Kim et al. [55], where the concept of a wetting front, (wf), is used to describe the maximum distance of penetration of the formulation into the tissue from the needle point. This wetting front at the end of the dosing procedure effectively becomes the outer surface of the depot.

If we take the $x,y$ plane as being parallel to the skin surface and the $z$ direction as the vertical axis, then the equation for the surface of the oblate spheroid is,

$$\begin{aligned} \frac{x^{2}+y^{2}}{wf_{h}^{2}}+\frac{z^{2}}{wf_{v}^{2}}=1\#\left( SEQ Equation \backslash* ARABIC 33 \right) \end{aligned}$$

where $wf_{h}$ and $wf_{v}$ denote the maximum extent of the water front in the horizontal and vertical directions, as shown in Figure 39.

Figure 39 Definition of the Maximum Extent of Wetting Front Parameters $\boldsymbol{w}\boldsymbol{f}_{\boldsymbol{h}}$ and $\boldsymbol{w}\boldsymbol{f}_{\boldsymbol{v}}$The surface of the spheroid represents the position of the wetting front relative to the point of injection


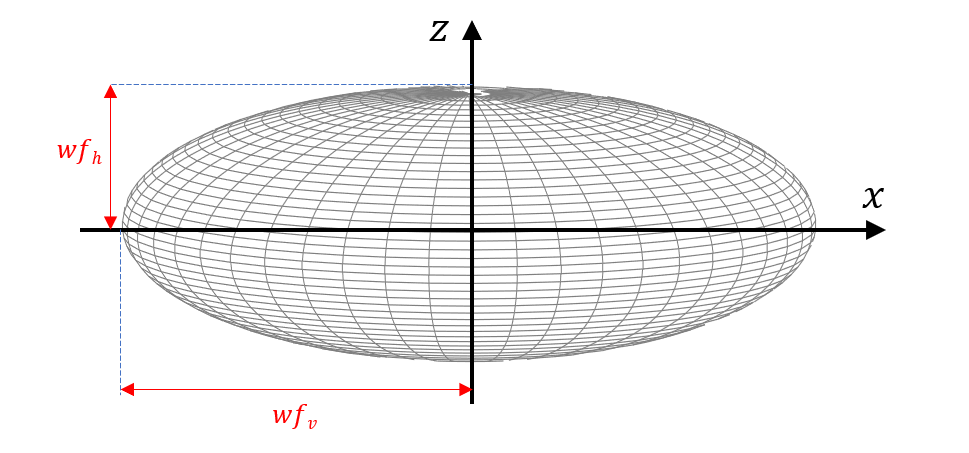


In terms of the indices $i$, $j$, $k$ of the voxel array, $x$, $y$ and $z$ can be expressed as, $x=d\left( i-n/2 \right)$, $y=d\cdot\left( j-n/2 \right)$, $z=d\cdot\left( k-n/2 \right)$, where $n$ is the overall dimension of the array in voxels and $d$ is the physical length of a voxel. The voxel with indices corresponding to $i=n/2$, $j=n/2$ and $k=n/2$ is the effective central point of the depot.

The dimensions of the voxels are typically selected such that the overall grid is 50% larger than $wf_{v}$ to ensure that, even with diffusion of the drug from the initial depot, the drug from the depot will still be contained within the voxel grid (software mass balance checks are in place to ensure this is the case). At the start of the simulation, for every voxel in the array, if the left-hand side of equation ($33$) is less than 1, that voxel is considered to be within the initial depot and will contain drug at $t=0$, otherwise it corresponds to the surrounding tissue. Similarly, all voxels for which the left-hand side of equation ($33$) is greater than 1 are assumed to have baseline levels of all the relevant proteins in the interstitial fluid and do not contain any API prior to the start of the simulation, i.e., at $t=0$.

Figure 40 Depot elongation vs product of volume and injection rate^(1/3). Data from Doughty et al. [13] and from Kim et al.[55] for low viscosity fluids. The solid line is equation (8) in manuscript.


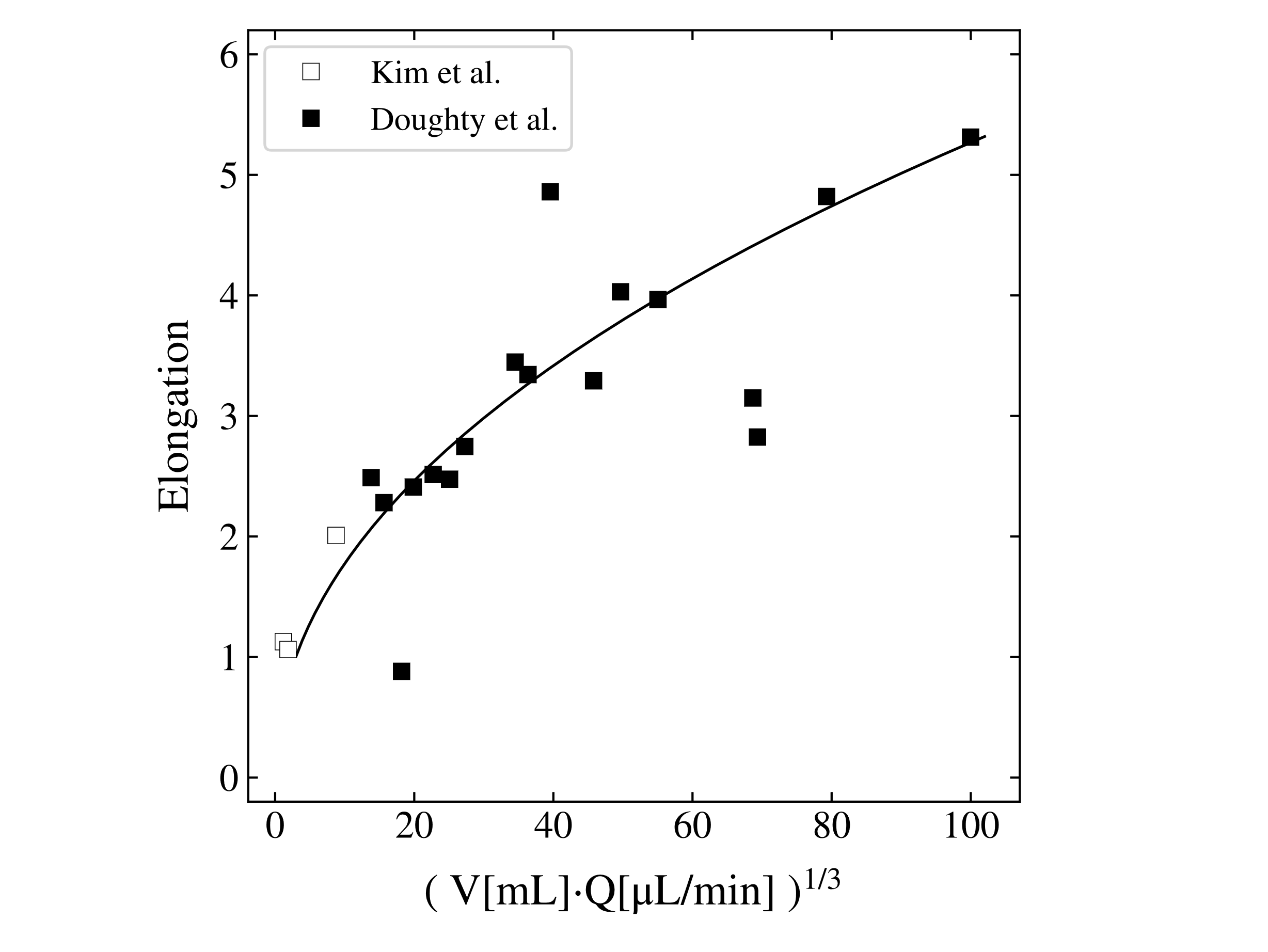


Figure 41 Evolution of depot waterfront in the vertical and horizontal direction for a simulated 500 μL injection at 100 μL/min (left panel) and 6000 μL/min (right panel)


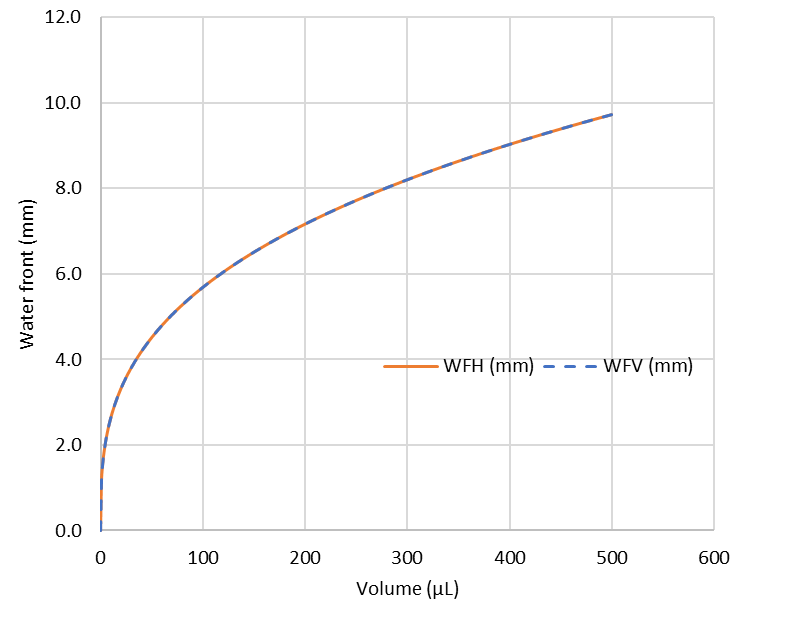

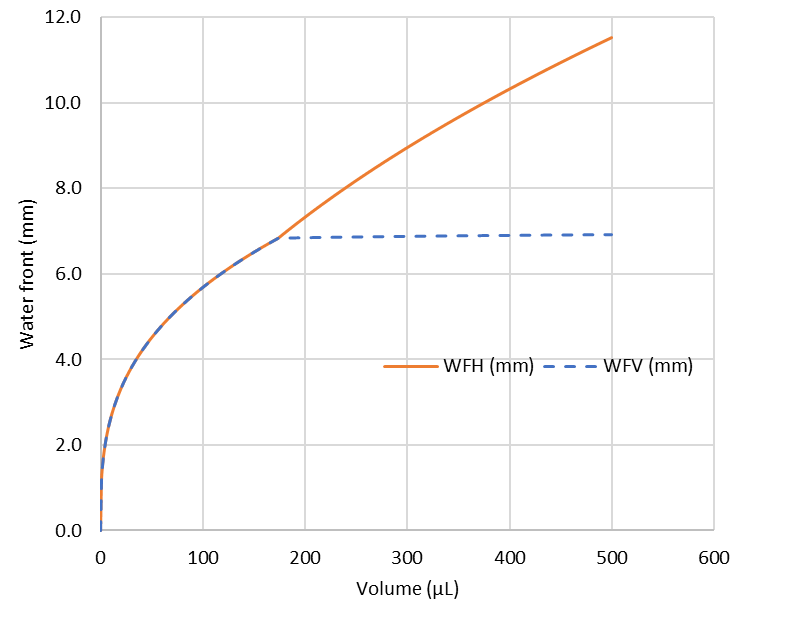


The validation of depot geometry using the injection parameters and the model proposed in the main manuscript is shown in Figure 42.

Figure 42 Verification of predicted depot shapes (vertical and horizontal water fronts) using measured data from references [13, 55, 56]


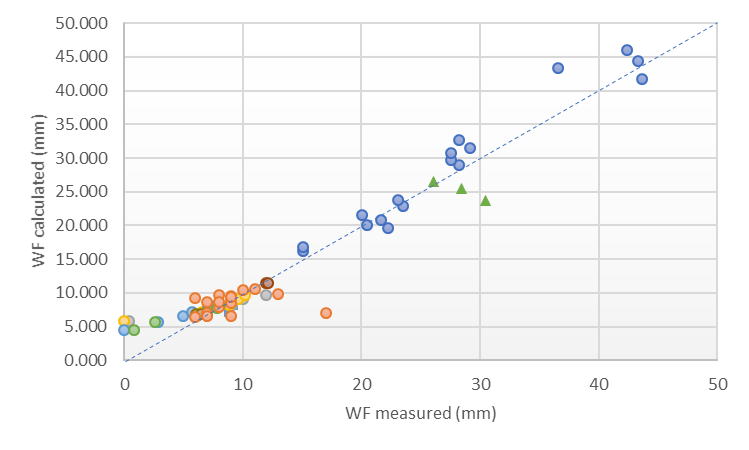


# Relative Concentration (RCS) within Depot

As part of setting the initial conditions for the simulation, we now assign a concentration of API to each voxel which is considered part of the depot. The process for doing this is based on concentration measurements in a subcutaneous injection depot published by Kim et al. [55].

These measurements of the relative concentration of the solution with the distance from the needle point, show a sigmoidal change in concentration ranging from roughly 0.2 at the point of injection to close to zero as we approach the maximum extent of the water front.

This concentration data, in both the horizontal and vertical directions, can be reasonably well approximated by fitting and equation of the form,

$$\begin{aligned} RCS\left( x \right)=\frac{RCS_{max}}{2}\left( 1+\text{erf}\left( \frac{\alpha\cdot wf-\beta x}{wf} \right) \right)\#\left( SEQ Equation \backslash* ARABIC 34 \right) \end{aligned}$$

where $\text{erf(}x)$ denotes the Gauss error function and $\alpha$, $\beta$ and $RCS_{max}$ are parameters which are derived from fitting. The figure below shows the data sampled from reference [55] and the fitted approximation by fitting $\alpha$, $\beta$ and $RCS_{max}$. For both sets of data the same fitted values for these parameters are used ($RCS_{max}=0.185$, $\alpha=2.49$ and $\beta=4.57$).

Figure 43 Data sampled from reference [55] and Fitted Function


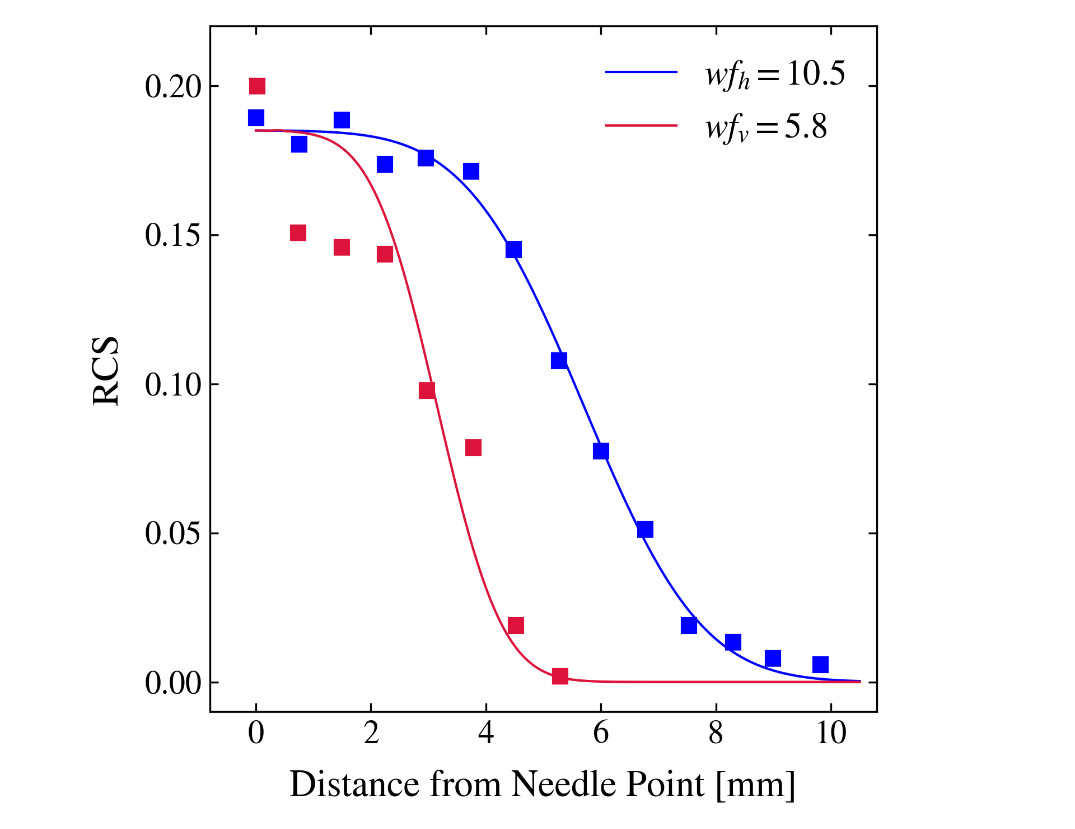


Although the above is effectively an empirical fit, it is nevertheless analogous to constantly infusing a solution into a porous absorbent material, for example, the packed bed of a chromatographic column, in which case, the concentration of the solute as a function of distance from the inlet is known to have the form of a modified error function [57].

We now require a means to calculate the RCS of each voxel in a 3-dimensional spheroidal depot. For a completely spherical depot, the extent of the water front is the same in all directions, however, in the case of the oblate spheroid, we need to calculate a relevant water front for all voxels within the depot. For voxels in the horizontal $x,y$ plane, the relevant water front distance is $wf_{h}$ and for voxels on the vertical $z$-axis it is $wf_{v}$. However, most voxels will not be in either of these locations. For voxels which are neither in the $x,y$ plane nor on the $z$-axis, we need to find a relevant intermediate value for $wf$.

Figure 44 Depot boundary as cross-section across the vertical plane. The points d_z_ and r_f_ are shown (black circles) for the highlighted voxel (red square)


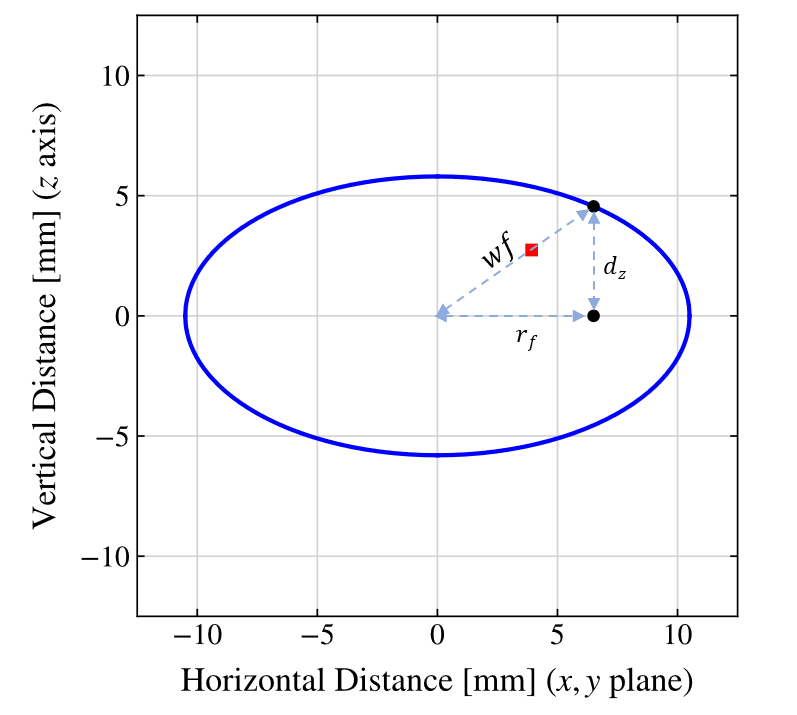


For each voxel at the location defined by $x,y,z$ within the depot, we extend the line defined by the needle point and the center of the voxel until it reaches the effective surface of the depot. We now calculate the radial distance ($r_{f}$) of this point on the surface projected onto the $x,y$ plane using equation (3) (Figure 44).

$$\begin{aligned} r_{f}\left( x,y,z \right)=\sqrt{\frac{wf_{h}^{2}\cdot wf_{v}^{2}\cdot\left( x^{2}+y^{2} \right)}{wf_{h}^{2}\cdot z^{2}+ wf_{v}^{2}\cdot\left( x^{2}+y^{2} \right)}}\#\left( SEQ Equation \backslash* ARABIC 35 \right) \end{aligned}$$

The $z$ co-ordinate of this surface point can now be calculated using equation ($36$).

$$\begin{aligned} d_{z}\left( x,y,z \right)=wf_{v}\sqrt{1-\frac{r_{f}\left( x,y,z \right)}{wf_{h}^{2}}}\#\left( SEQ Equation \backslash* ARABIC 36 \right) \end{aligned}$$

We can now calculate the effective water front distance ($wf$) from equation ($37$).

$$\begin{aligned} w_{f}\left( x,y,z \right)=\sqrt{{r_{f}\left( x,y,z \right)}^{2}+{d_{z}\left( x,y,z \right)}^{2}}\#\left( SEQ Equation \backslash* ARABIC 37 \right) \end{aligned}$$

For each voxel in the depot, we can now calculate RCS using equation (6). Where the fitted parameters have been replaced by their optimized values ($RCS_{max}=0.185$, $\alpha=2.49$ and $\beta=4.57$).

$$\begin{aligned} RCS\left( x,y,z \right)=0.0925\left( 1+\text{erf}\left( \frac{2.49\cdot w_{f}\left( x,y,z \right)-4.57\sqrt{x^{2}+y^{2}+z^{2}}}{w_{f}\left( x,y,z \right)} \right) \right)\#\left( SEQ Equation \backslash* ARABIC 38 \right) \end{aligned}$$

As shown in Figure 45, we can use equation ($38$) plot a 3-dimensional representation of the variation of the initial API concentration within the depot at the start of the simulation (in this example, the figure shows the concentration from a horizontal cross section through the center of the depot).

Figure 45 3D Plot of RCS calculated in the horizontal plane using equation ($\mathbf{38}$)


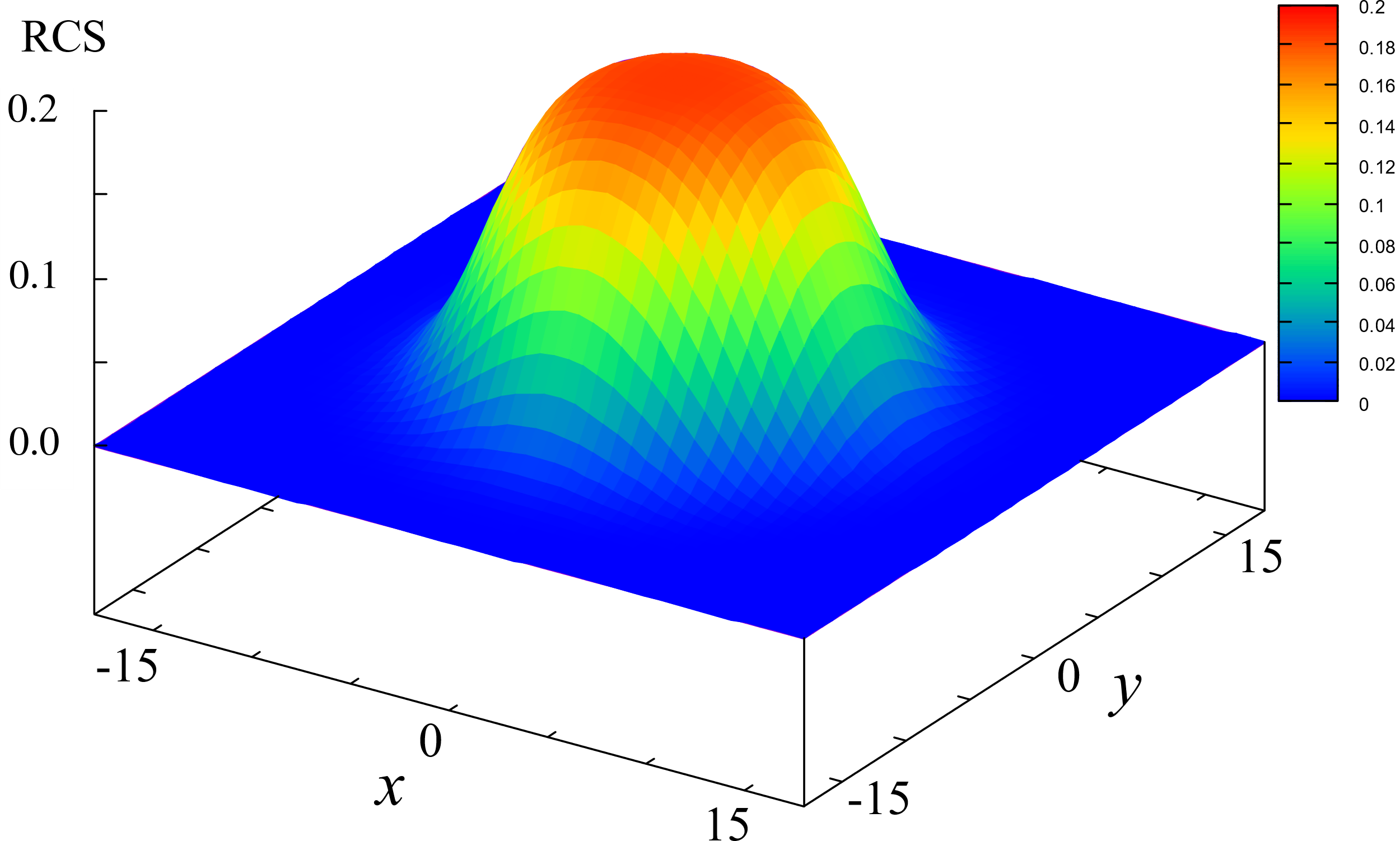


Contour plots generated using equation ($38$) show the contrast between the top-down view and the side on view in accordance with the data from reference [55] (Figure 46).

Figure 46 Contour plots of RCS calculated in the horizontal plane (top-down view) and in the vertical plane (side view) using equation ($\mathbf{38}$)


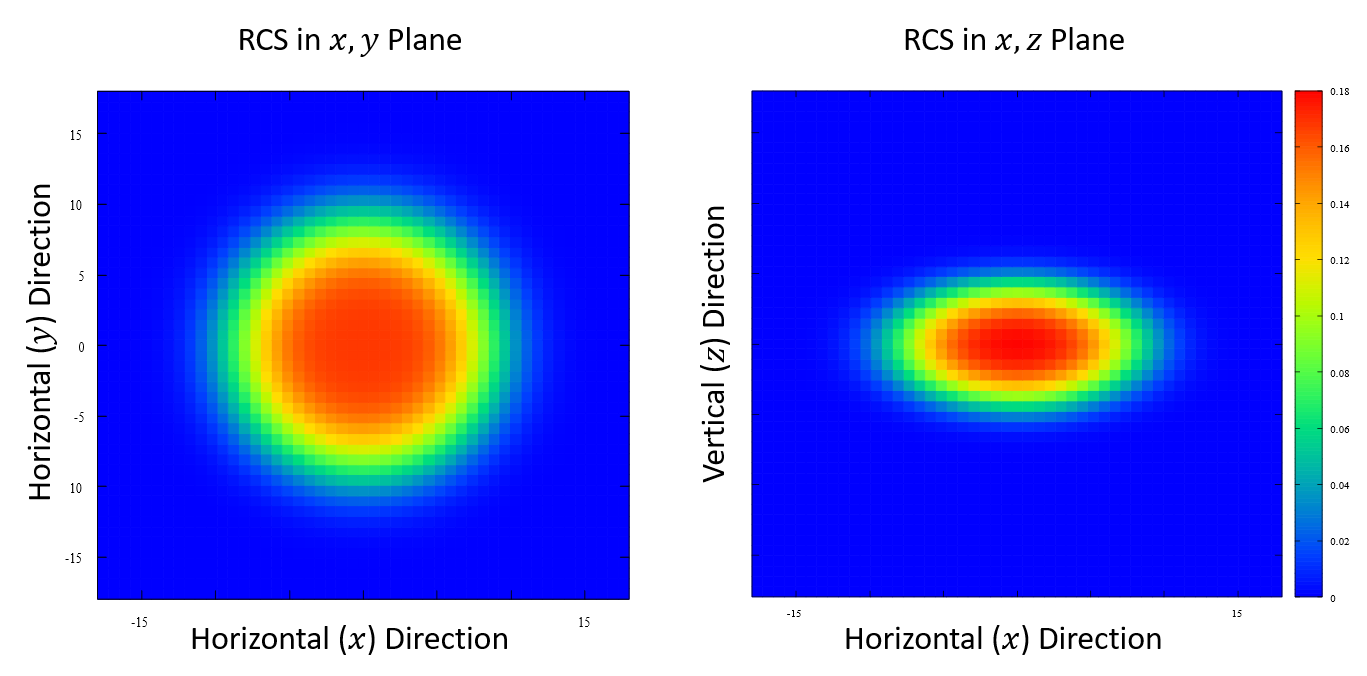


When the voxel array is correctly initialised, the sum of the amount of API in each voxel must be exactly equivalent to the net administered dose (after correcting for any initial losses via the puncture hole). Thus, we can say,

$$\begin{aligned} Dose=f_{c}\cdot C_{f}\cdot V_{vox}\sum_{i=1}^{n} \sum_{j=1}^{n} \sum_{k=1}^{n} \left( RCS\left( i,j,k \right) \right)\#\left( SEQ Equation \backslash* ARABIC 39 \right) \end{aligned}$$

where $C_{f}$ is the concentration of API in the formulation, $V_{vox}$ is the volume of a single voxel and $f_{c}$ a correction factor which is applied in order to ensure that the sum of the amount of drug over all voxels is exactly equivalent to the dose. The initial concentration in a given voxel is there for set to,

$$\begin{aligned} C\left( i,j,k \right)=RCS\left( i,j,k \right)\cdot C_{f}\cdot f_{c}\#\left( SEQ Equation \backslash* ARABIC 40 \right) \end{aligned}$$

During the formation of the depot, the interstitial proteins, which are freely mobile in the ECM, will be displaced from the volume of tissue occupied by the injected formulation. In the core of the depot where the RCS is close to RCS_max_, it is assumed that all of the mobile proteins are completely flushed out by the formulation, but some mixing will occur towards the water front. Thus, concentrations of interstitial proteins, immediately following the dose administration, are considered to follow the inverse of that predicted by equation (36), i.e., where RCS is RCS_max_, the protein concentration is zero and where RCS is zero, the protein concentration is set to the baseline tissue value. Over time, the concentrations of the proteins will recover to their previous baseline values, as a result of secretion from blood capillaries and diffusion from the surrounding tissue.

# Supplementary data for tissue back pressure

Table 10 Average back pressures (kPa) measured by Doughty et al [13], Patte et al. [58] and Allmendinger et al. [56] with standard deviation and predicted values using the model described in main manuscript

| Ref | Formulation Viscosity Pa.s | Volume (mL) | Flow rate (uL/min) | Back pressure (kPa) | Predicted pressure (kPa) | SD measured (kPa) |
| --- | --- | --- | --- | --- | --- | --- |
| [13] | 0.001 | 1 | 6000 | 24 | 29.9 | 3.4 |
| [13] | 0.001 | 1 | 12000 | 48.8 | 55.4 | 30.0 |
| [13] | 0.001 | 2 | 333 | 4.7 | 3.2 | 2.6 |
| [13] | 0.001 | 2 | 500 | 8.6 | 4.1 | 5.3 |
| [13] | 0.001 | 2 | 1000 | 5.7 | 6.6 | 1.2 |
| [13] | 0.001 | 2 | 2000 | 10.7 | 11.3 | 6.4 |
| [13] | 0.001 | 2 | 6000 | 36.7 | 29.2 | 8.3 |
| [13] | 0.001 | 2 | 12000 | 34.2 | 54.1 | 32.6 |
| [13] | 0.001 | 3 | 12000 | 59.1 | 52.4 | 22.0 |
| [13] | 0.001 | 5 | 167 | 3.6 | 2.1 | 1.3 |
| [13] | 0.001 | 5 | 333 | 3 | 2.8 | 0.6 |
| [13] | 0.001 | 5 | 500 | 7.4 | 3.5 | 6.9 |
| [13] | 0.001 | 5 | 1000 | 8.2 | 5.7 | 7.2 |
| [13] | 0.001 | 10 | 167 | 4.2 | 1.8 | 2.6 |
| [13] | 0.001 | 10 | 333 | 4.3 | 2.4 | 2.1 |
| [13] | 0.001 | 10 | 500 | 2.1 | 3.0 | 0.4 |
| [13] | 0.001 | 10 | 1000 | 7.4 | 4.7 | 7.8 |
| [58] | 0.001 | 0.3 | 10 | 0.809 | 0.2 | 0.8 |
| [58] | 0.001 | 0.3 | 50 | 1.828 | 0.3 | 3.1 |
| [58] | 0.001 | 0.3 | 100 | 2.518 | 0.7 | 3.6 |
| [58] | 0.001 | 0.3 | 500 | 6.259 | 3.6 | 6.9 |
| [56] | 0.001 | 2.5 | 1500 | 4.5 | 8.8 | 4.7 |
| [56] | 0.010 | 2.5 | 1500 | 13.7 | 13.0 | 7.4 |
| [56] | 0.020 | 2.5 | 1500 | 15.3 | 17.7 | 5.0 |
| [56] | 0.100 | 2.5 | 1500 | 61.2 | 55.2 | 1.7 |
| [56] | 0.001 | 2.5 | 6000 | 4.1 | 28.7 | 2.3 |
| [56] | 0.010 | 2.5 | 6000 | 13.8 | 44.2 | 7.8 |
| [56] | 0.020 | 2.5 | 6000 | 25.4 | 61.5 | 12.1 |
| [56] | 0.001 | 2.5 | 12000 | 8.6 | 53.2 | 3.5 |
| [56] | 0.010 | 2.5 | 12000 | 22.0 | 82.7 | 15.7 |
| [56] | 0.020 | 2.5 | 12000 | 41.4 | 115.4 | 28.4 |

Figure 47 Calculated vs measured SC tissue backpressure during injection (plateau). Open symbols show measured values ± 1 SD. Solid line represents unity.


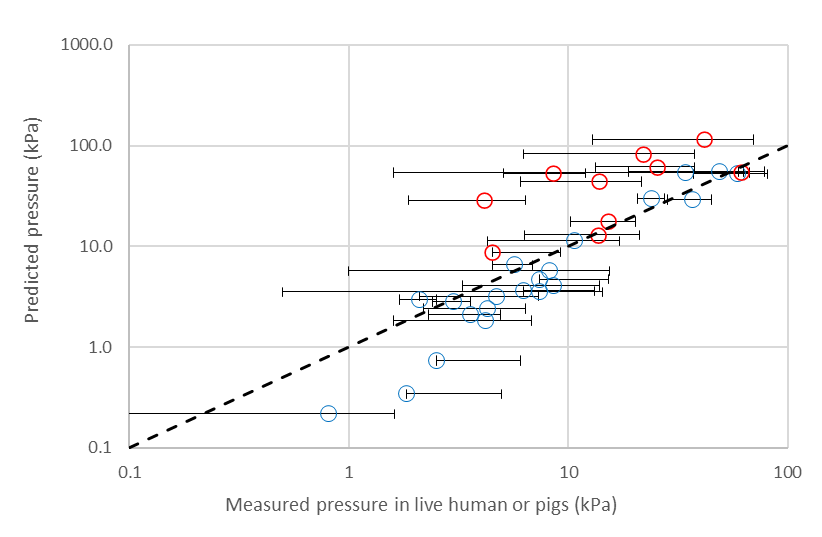


**Table 11 Data for the validation of adipose tissue backflow calculation**

| **Needle length (mm)** | **Needle gauge** | **Volume (μL)** | **Injection speed (μL/s)** | **IF viscosity (mPa.s)** | **Tissue** | **Ref** | **Total time (inj+ wait) (s)** | **Wait time post inj. (s)** | **Observed leakage (μL)** | **Predicted leakage (μL)** | **SD Observed leakage (μL)** |
| --- | --- | --- | --- | --- | --- | --- | --- | --- | --- | --- | --- |
| 6 | 30 | 400 | 150 | 2.08 | Abdomen | [59] | 20 |  | 0.61 | 1.84 | 0.33 |
| 6 | 30 | 400 | 300 | 2.08 | Abdomen | [59] | 20 |  | 0.4 | 1.77 | 0.33 |
| 6 | 30 | 400 | 450 | 2.08 | Abdomen | [59] | 20 |  | 0.86 | 1.79 | 0.33 |
| 6 | 30 | 800 | 150 | 2.08 | Abdomen | [59] | 20 |  | 0.47 | 1.84 | 0.33 |
| 6 | 30 | 800 | 300 | 2.08 | Abdomen | [59] | 20 |  | 0.47 | 1.70 | 0.33 |
| 6 | 30 | 800 | 450 | 2.08 | Abdomen | [59] | 20 |  | 0.55 | 1.61 | 0.33 |
| 6 | 30 | 1200 | 150 | 2.08 | Abdomen | [59] | 20 |  | 0.93 | 1.67 | 0.33 |
| 6 | 30 | 1200 | 300 | 2.08 | Abdomen | [59] | 20 |  | 0.78 | 1.52 | 0.33 |
| 6 | 30 | 1200 | 450 | 2.08 | Abdomen | [59] | 20 |  | 1.13 | 1.44 | 0.33 |
| 6 | 30 | 1600 | 150 | 2.08 | Abdomen | [59] | 20 |  | 2.27 | 1.52 | 0.33 |
| 6 | 30 | 1600 | 300 | 2.08 | Abdomen | [59] | 20 |  | 1.41 | 1.38 | 0.33 |
| 6 | 30 | 1600 | 450 | 2.08 | Abdomen | [59] | 20 |  | 0.59 | 1.30 | 0.33 |
| 6 | 30 | 400 | 150 | 2.08 | Thigh | [59] | 20 |  | 1.05 | 3.67 | 0.80 |
| 6 | 30 | 400 | 450 | 2.08 | Thigh | [59] | 20 |  | 1.6 | 4.01 | 0.80 |
| 6 | 30 | 800 | 300 | 2.08 | Thigh | [59] | 20 |  | 2.33 | 4.13 | 0.80 |
| 6 | 30 | 1600 | 150 | 2.08 | Thigh | [59] | 20 |  | 5.65 | 3.74 | 0.80 |
| 6 | 30 | 1600 | 450 | 2.08 | Thigh | [59] | 20 |  | 2.45 | 3.21 | 0.81 |
| 6 | 32 | 400 | 100 | 1.65 | Anesthetized pigs | [59] |  | 6 | 1.65 | 0.65 | 0.44 |
| 6 | 31 | 400 | 100 | 1.65 | Anesthetized pigs | [59] |  | 6 | 2.58 | 0.97 | 0.43 |
| 6 | 32 | 400 | 100 | 1.65 | Anesthetized pigs | [59] |  | 6 | 1.59 | 0.65 | 0.44 |
| 6 | 32 | 400 | 100 | 1.65 | Anesthetized pigs | [59] |  | 6 | 3.06 | 0.65 | 0.43 |
| 6 | 32 | 400 | 100 | 1.65 | Anesthetized pigs | [59] |  | 0 | 4.08 | 6.53 | 0.43 |
| 6 | 32 | 400 | 100 | 1.65 | Anesthetized pigs | [59] |  | 3 | 1.72 | 0.90 | 0.43 |
| 6 | 32 | 400 | 100 | 1.65 | Anesthetized pigs | [59] |  | 10 | 1.86 | 1.10 | 0.43 |
| 5 | 32 | 200 | 150 | 2.08 | Abdomen | [60] |  | 7 | 0.7 | 1.05 | 0.80 |
| 8 | 32 | 200 | 150 | 2.08 | Abdomen | [60] |  | 7 | 0.6 | 0.40 | 0.90 |
| 5 | 32 | 600 | 150 | 2.08 | Abdomen | [60] |  | 7 | 1.4 | 1.30 | 2.00 |
| 8 | 32 | 600 | 150 | 2.08 | Abdomen | [60] |  | 7 | 0.6 | 0.62 | 0.80 |
| 4.5 | 32 | 50 | 150 | 1.65 | fresh pork rind | [61] |  | 10 | 0.30 | 0.20 | 0.04 |
| 6 | 32 | 50 | 150 | 1.65 | fresh pork rind | [61] |  | 10 | 0.41 | 0.26 | 0.04 |
| 8 | 32 | 50 | 150 | 1.65 | fresh pork rind | [61] |  | 10 | 0.40 | 0.35 | 0.05 |
| 10 | 32 | 50 | 150 | 1.65 | fresh pork rind | [61] |  | 10 | 0.42 | 0.43 | 0.05 |
| 12 | 32 | 50 | 150 | 1.65 | fresh pork rind | [61] |  | 10 | 0.33 | 0.52 | 0.07 |
| 4.5 | 32 | 100 | 150 | 1.65 | fresh pork rind | [61] |  | 10 | 0.59 | 0.20 | 0.07 |
| 6 | 32 | 100 | 150 | 1.65 | fresh pork rind | [61] |  | 10 | 0.59 | 0.26 | 0.07 |
| 8 | 32 | 100 | 150 | 1.65 | fresh pork rind | [61] |  | 10 | 0.43 | 0.35 | 0.05 |
| 10 | 32 | 100 | 150 | 1.65 | fresh pork rind | [61] |  | 10 | 0.43 | 0.43 | 0.03 |
| 12 | 32 | 100 | 150 | 1.65 | fresh pork rind | [61] |  | 10 | 0.38 | 0.52 | 0.04 |
| 4.5 | 32 | 200 | 150 | 1.65 | fresh pork rind | [61] |  | 10 | 0.76 | 1.16 | 0.09 |
| 6 | 32 | 200 | 150 | 1.65 | fresh pork rind | [61] |  | 10 | 0.50 | 0.71 | 0.07 |
| 8 | 32 | 200 | 150 | 1.65 | fresh pork rind | [61] |  | 10 | 0.68 | 0.40 | 0.06 |
| 10 | 32 | 200 | 150 | 1.65 | fresh pork rind | [61] |  | 10 | 0.48 | 0.43 | 0.05 |
| 12 | 32 | 200 | 150 | 1.65 | fresh pork rind | [61] |  | 10 | 0.61 | 0.52 | 0.11 |
| 6 | 30 | 400 | 150 | 2.08 | Abdomen | [62] | 20 |  | 0.9 | 1.87 | 0.15 |
| 6 | 30 | 400 | 450 | 2.08 | Abdomen | [62] | 20 |  | 0.4 | 1.80 | 0.15 |
| 6 | 30 | 1600 | 150 | 2.08 | Abdomen | [62] | 20 |  | 2.3 | 1.52 | 0.52 |
| 6 | 30 | 1600 | 450 | 2.08 | Abdomen | [62] | 20 |  | 0.6 | 1.30 | 0.52 |
| 6 | 30 | 400 | 150 | 2.08 | Thigh | [62] | 20 |  | 1.6 | 4.00 | 0.18 |
| 6 | 30 | 400 | 450 | 2.08 | Thigh | [62] | 20 |  | 1 | 4.15 | 0.18 |
| 6 | 30 | 1600 | 150 | 2.08 | Thigh | [62] | 20 |  | 5.6 | 3.74 | 0.94 |
| 6 | 30 | 1600 | 450 | 2.08 | Thigh | [62] | 20 |  | 2.5 | 3.21 | 0.94 |

Figure 48 Predicted and measured leakage through puncture holes. Data from Supplemental Table 11.


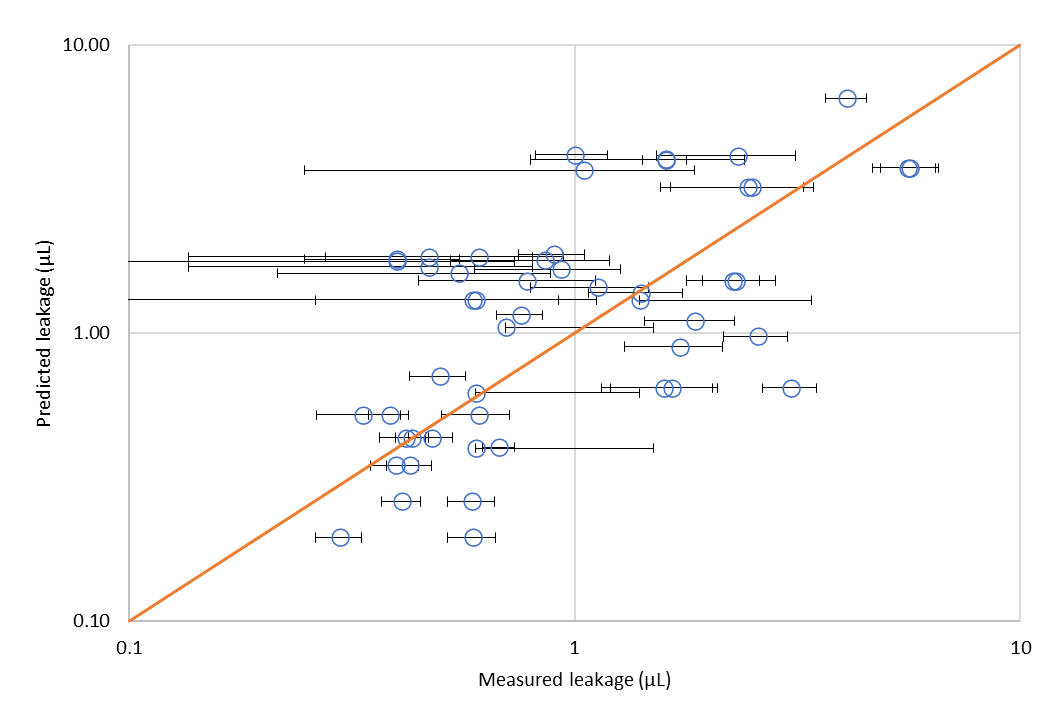


Figure 49 Predicted pressure time profile for a 10mL injection of a 7.2 mPa.s solution with 25,000 U/mL rHuPH20 in pigs. Sensitivity analysis on the hyaluronidase V_max_ (A) and injection rate (B)


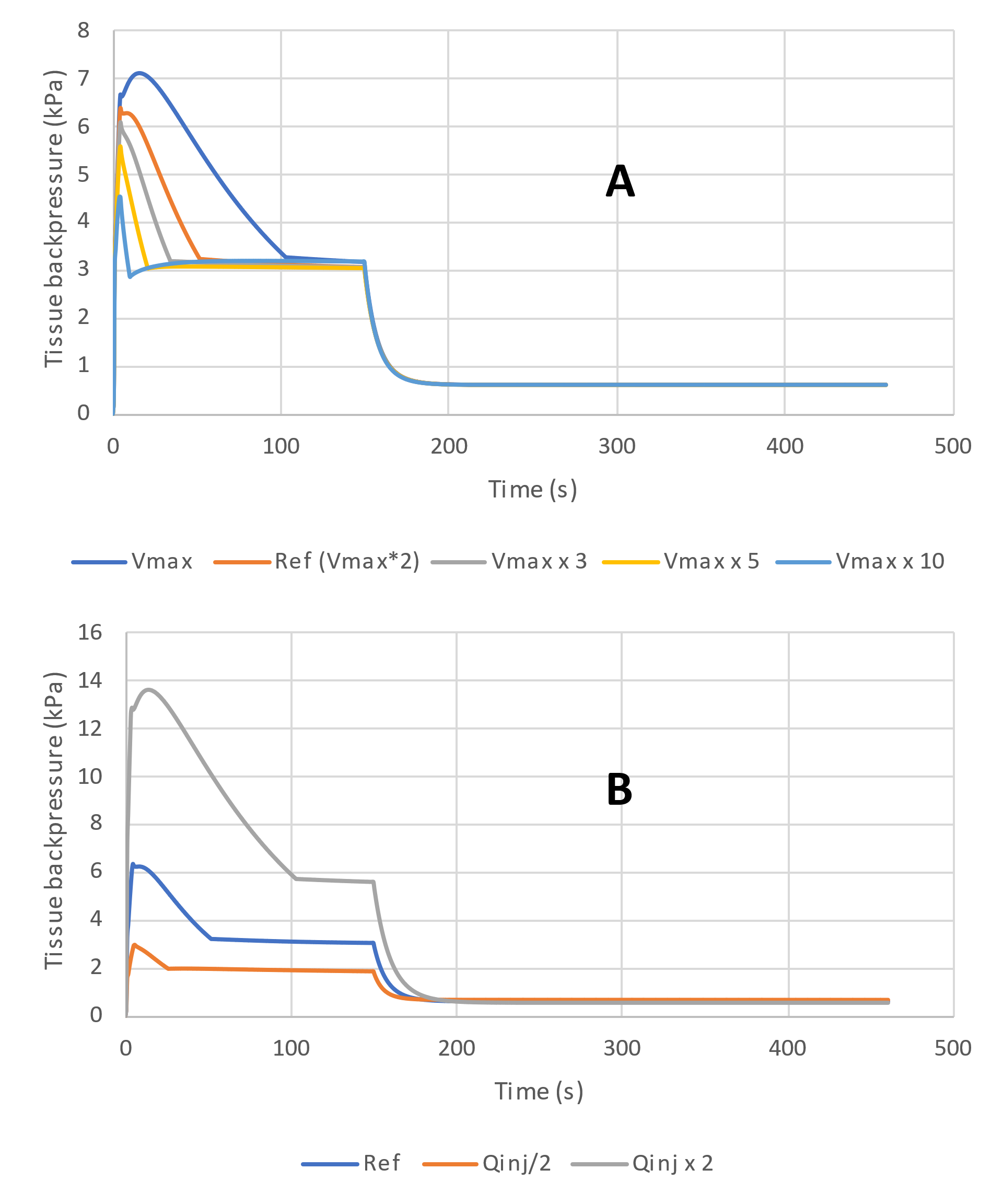


# Shear strain during injection in the SC tissue

Berriaud et al. [63] performed viscosity measurements at different shear rates and have shown that the hyaluronan solutions have a Newtonian region at low shear rates and then become rheo-thinning above a critical shear rate of around 3 s-1. It is hard to estimate the shear rates of the interstitial fluid as it is pushed from the depot through the ECM porosity. Using 0.1 as fractional IF volume (pore filled with IF), the fluid velocity $\vartheta$ is approximated by the surface area of the ellipsoid ($S_{el}$) and injection rate $Q_{inj}$using the following equation:

$$\vartheta=\frac{Q_{inj}}{\left( 0.1\times4\pi\left( \frac{{WFH\left( t \right)}^{3.2}+2\left( WFH\left( t \right)WFV\left( t \right) \right)^{1.6}}{3} \right)^{\frac{1}{1.6}} \right)^{\frac{2}{3}}}$$

The IF velocity drops rapidly as the surface area of the depot increases with time during injection.

The pore size (p) is estimated by:

$$\begin{aligned} p=\left( \pi{\varepsilon WFH\left( t \right)}^{2}WFV\left( t \right)\times0.1 \right)^{\frac{1}{3}}\#\left( SEQ Equation \backslash* ARABIC 25 \right) \end{aligned}$$

Based on the above equation the pore size increases with time. In order to lead provide for a worst-case scenario estimation of the shear rate, we can keep it constant to around 2.2 microns (average on first second of injection). The shear rate is estimated by $\vartheta/p$.

Simulations for example for a 500 µL injection at 6000 µL/min, show that the shear rate is above 3 s^-1^ only for the first 75 milliseconds (for a total injection time of 5 s). Simulations for a 500 µL injection at 12000 µL/min, show that the shear rate is above 3 s^-1^ only for the first 100 millisecond (for a total injection time of 2.5s). It is concluded that in most clinically relevant injection rates, during more than 95% of the injection time, the movement of IF in the tissue pores will be Newtonian.

# References

1. Sjöström, C.D., et al., *Body Compartment and Subcutaneous Adipose Tissue Distribution - Risk Factor Patterns in Obese Subjects.* Obesity Research, 1995. **3**(1): p. 9-22.

2. Kindblom, J.M., et al., *BMI Changes During Childhood and Adolescence as Predictors of Amount of Adult Subcutaneous and Visceral Adipose Tissue in Men: The GOOD Study.* Diabetes, 2009. **58**(4): p. 867-874.

3. Hübers, M., et al., *Association between fat mass, adipose tissue, fat fraction per adipose tissue, and metabolic risks: a cross-sectional study in normal, overweight, and obese adults.* European Journal of Clinical Nutrition, 2019. **73**(1): p. 62-71.

4. Meyer zu Schwabedissen, H.E., et al., *Human multidrug and toxin extrusion 1 (MATE1/SLC47A1) transporter: functional characterization, interaction with OCT2 (SLC22A2), and single nucleotide polymorphisms.* Am J Physiol Renal Physiol, 2010. **298**(4): p. F997-F1005.

5. Ellis, K.J., et al., *Accuracy of dual-energy x-ray absorptiometry for body-composition measurements in children.* Am J Clin Nutr, 1994. **60**(5): p. 660-5.

6. Pietrobelli, A., et al., *Body mass index as a measure of adiposity among children and adolescents: A validation study.* The Journal of Pediatrics, 1998. **132**(2): p. 204-210.

7. Santana, H., et al., *Relation between body composition, fat distribution, and lung function in elderly men.* The American journal of clinical nutrition, 2001. **73**(4): p. 827-31.

8. Gallagher, D., et al., *Healthy percentage body fat ranges: an approach for developing guidelines based on body mass index.* The American journal of clinical nutrition, 2000. **72**(3): p. 694-701.

9. Tchoukalova, Y.D., et al., *Subcutaneous adipocyte size and body fat distribution.* The American journal of clinical nutrition, 2008. **87**(1): p. 56-63.

10. Jain, S.M., et al., *Evaluation of skin and subcutaneous tissue thickness at insulin injection sites in Indian, insulin naïve, type-2 diabetic adult population.* Indian journal of endocrinology and metabolism, 2013. **17**(5): p. 864-870.

11. Ezure, T. and S. Amano, *Influence of subcutaneous adipose tissue mass on dermal elasticity and sagging severity in lower cheek.* J Skin Research and Technology, 2010. **16**(3): p. 332-338.

12. Sano, H., Y. Hokazono, and R. Ogawa, *Distensibility and Gross Elasticity of the Skin at Various Body Sites and Association with Pathological Scarring: A Case Study.* The Journal of clinical and aesthetic dermatology, 2018. **11**(6): p. 15-18.

13. Doughty, D.V., et al., *Understanding Subcutaneous Tissue Pressure for Engineering Injection Devices for Large-Volume Protein Delivery.* Journal of Pharmaceutical Sciences, 2016. **105**(7): p. 2105-2113.

14. Gealekman, O., et al., *Depot-Specific Differences and Insufficient Subcutaneous Adipose Tissue Angiogenesis in Human Obesity.* Circulation, 2011.

15. Belligoli, A., et al., *Characterization of subcutaneous and omental adipose tissue in patients with obesity and with different degrees of glucose impairment.* Scientific Reports, 2019. **9**(1).

16. Schacht, V., et al., *Anatomy of the Subcutaneous Lymph Vascular Network of the Human Leg in Relation to the Great Saphenous Vein.* The Anatomical Record, 2009. **292**(1): p. 87-93.

17. Suami, H. and A. Shinaoka, *The methodology of lymphatic anatomy studies in a cadaver model: an overview.* Plastic and Aesthetic Research, 2019. **2019**.

18. Suami, H. and M.F. Scaglioni, *Anatomy of the Lymphatic System and the Lymphosome Concept with Reference to Lymphedema.* Seminars in plastic surgery, 2018. **32**(1): p. 5-11.

19. Thomsen, M., *Inspection of subcutaneous injections by x-ray absorption tomography*, in *Niels Bohr Institute, University of Copenhagen*. 2011, University of Copenhagen. p. 119.

20. Laforest, S., et al., *Comparative analysis of three human adipocyte size measurement methods and their relevance for cardiometabolic risk: Comparative Analysis of Adipocyte Sizing Methods.* Obesity, 2017. **25**(1): p. 122-131.

21. Salans, L.B., S.W. Cushman, and R.E. Weismann, *Studies of Human Adipose Tissue ADIPOSE CELL SIZE AND NUMBER IN NONOBESE AND OBESE PATIENTS.* Journal of Clinical Investigation, 1973. **52**(4): p. 929-941.

22. Löfgren, P., et al., *Major gender differences in the lipolytic capacity of abdominal subcutaneous fat cells in obesity observed before and after long-term weight reduction.* The Journal of clinical endocrinology and metabolism, 2002. **87**(2): p. 764-71.

23. Wiig, H. and M.A. Swartz, *Interstitial fluid and lymph formation and transport: physiological regulation and roles in inflammation and cancer.* Physiol Rev, 2012. **92**(3): p. 1005-60.

24. Levick, J.R., *Capillary filtration-absorption balance reconsidered in light of dynamic extravascular factors.* Exp Physiol, 1991. **76**(6): p. 825-57.

25. Dempster, W. and G. Gaughran, *Properties of Body Segments Based on size and weight.* American Journal of Anatomy, 1967. **120**(1): p. 33-54.

26. Tansey, E.A., et al., *Understanding basic vein physiology and venous blood pressure through simple physical assessments.* 2019. **43**(3): p. 423-429.

27. Uren, R.F., *Lymphatic drainage of the skin.* Annals of surgical oncology, 2004. **11**(3 Suppl): p. 85S.

28. Michel, C.C., T.E. Woodcock, and F.-R.E. Curry, *Understanding and extending the Starling principle.* 2020. **64**(8): p. 1032-1037.

29. Michel, C.C., *Capillary permeability and how it may change.* The Journal of Physiology, 1988. **404**(1): p. 1-29.

30. Levick, J.R. and C.C. Michel, *Microvascular fluid exchange and the revised Starling principle.* Cardiovasc Res, 2010. **87**(2): p. 198-210.

31. Renkin, E.M., et al., *Influence of venous pressure on plasma-lymph transport in the dog's paw: convective and dissipative mechanisms.* Microvasc Res, 1977. **14**(2): p. 191-204.

32. Taylor, N.A., M.J. Tipton, and G.P. Kenny, *Considerations for the measurement of core, skin and mean body temperatures.* J Therm Biol, 2014. **46**: p. 72-101.

33. Astrup, A., J. Bülow, and J. Madsen, *Skin temperature and subcutaneous adipose blood flow in man.* Scandinavian Journal of Clinical and Laboratory Investigation, 1980. **40**(2): p. 135-138.

34. Webb, P., *Temperatures of skin, subcutaneous tissue, muscle and core in resting men in cold, comfortable and hot conditions.* European Journal of Applied Physiology and Occupational Physiology, 1992. **64**(5): p. 471-476.

35. Rowell, L.B., et al., *Human Cardiovascular Adjustments to Rapid Changes in Skin Temperature during Exercise.* Circulation Research, 1969. **24**(5): p. 711-724.

36. Stelfox, H.T., et al., *Hemodynamic monitoring in obese patients: The impact of body mass index on cardiac output and stroke volume*.* Critical Care Medicine, 2006. **34**(4).

37. Valentin, J., *Basic anatomical and physiological data for use in radiological protection: reference values.* Annals of the ICRP, 2002. **32**(3-4): p. 1-277.

38. Flamm, S.D., et al., *Redistribution of regional and organ blood volume and effect on cardiac function in relation to upright exercise intensity in healthy human subjects.* Circulation, 1990. **81**(5): p. 1550-9.

39. Williams, L.R. and R.W. Leggett, *Reference values for resting blood flow to organs of man.* Clinical Physics and Physiological Measurement, 1989. **10**(3): p. 187-217.

40. Tan, G.D., et al., *Upper and lower body adipose tissue function: a direct comparison of fat mobilization in humans.* Obes Res, 2004. **12**(1): p. 114-8.

41. Linde, B., et al., *Adipose tissue and skeletal muscle blood flow during mental stress.* American Journal of Physiology-Endocrinology and Metabolism, 1989. **256**(1): p. E12-E18.

42. Frayn, K.N. and F. Karpe, *Regulation of human subcutaneous adipose tissue blood flow.* Int J Obes (Lond), 2014. **38**(8): p. 1019-26.

43. McQuaid, S.E., et al., *Downregulation of adipose tissue fatty acid trafficking in obesity: a driver for ectopic fat deposition?* Diabetes, 2011. **60**(1): p. 47-55.

44. Berger, D. and J. Takala, *Determinants of systemic venous return and the impact of positive pressure ventilation.* Annals of Translational Medicine, 2018. **6**(18).

45. Rothe, C.F., *Mean circulatory filling pressure: its meaning and measurement.* Journal of Applied Physiology, 1993. **74**(2): p. 499-509.

46. Johnson, P.C., *Effect of Venous Pressure on Mean Capillary Pressure and Vascular Resistance in the Intestine.* Circulation Research, 1965. **16**(3): p. 294-300.

47. Guyton, A.C., *Determination of Cardiac Output By Equating Venous Return Curves With Cardiac Response Curves.* 1955. **35**(1): p. 123-129.

48. Rippe, B. and B. Haraldsson, *Transport of macromolecules across microvascular walls: the two-pore theory.* Physiological Reviews, 1994. **74**(1): p. 163-219.

49. D. J. Meyer, J. and V.H. Huxley, *Differential sensitivity of exchange vessel hydraulic conductivity to atrial natriuretic peptide.* 1990. **258**(2): p. H521-H528.

50. Huxley, V.H., et al., *Increased capillary hydraulic conductivity induced by atrial natriuretic peptide.* 1987. **60**(2): p. 304-307.

51. Curry, F.E., *Effect of temperature on hydraulic conductivity of single capillaries.* 1981. **240**(1): p. H29-H32.

52. Maeda, A., et al., *Regulation of the glucose supply from capillary to tissue examined by developing a capillary model.* The Journal of Physiological Sciences, 2018. **68**(4): p. 355-367.

53. Sell, H., et al., *Adipose Dipeptidyl Peptidase-4 and Obesity: Correlation with insulin resistance and depot-specific release from adipose tissue in vivo and in vitro.* Diabetes Care, 2013. **36**(12): p. 4083-4090.

54. Standeven, K.F., et al., *Neprilysin, obesity and the metabolic syndrome.* International journal of obesity (2005), 2011. **35**(8): p. 1031-1040.

55. Kim, H., H. Park, and S.J. Lee, *Effective method for drug injection into subcutaneous tissue.* Scientific Reports, 2017. **7**(1).

56. Allmendinger, A., et al., *Measuring Tissue Back-Pressure - In Vivo Injection Forces During Subcutaneous Injection.* Pharmaceutical Research, 2015. **32**(7): p. 2229-2240.

57. Glueckauf, E., *Theory of chromatography. Part 9. The “theoretical plate” concept in column separations.* Transactions of the Faraday Society, 1955. **51**(0): p. 34-44.

58. Patte, C., et al., *Effect of infusion rate and indwelling time on tissue resistance pressure in small-volume subcutaneous infusion like in continuous subcutaneous insulin infusion.* Diabetes technology & therapeutics, 2013. **15**(4): p. 289-94.

59. Præstmark, K.A., et al., *Injection Technique and Pen Needle Design Affect Leakage From Skin After Subcutaneous Injections.* J Diabetes Sci Technol, 2016. **10**(4): p. 914-22.

60. Ignaut, D.A. and H. Fu, *Comparison of Insulin Diluent Leakage Postinjection Using Two Different Needle Lengths and Injection Volumes in Obese Patients with Type 1 or Type 2 Diabetes Mellitus.* Journal of Diabetes Science and Technology, 2012. **6**(2): p. 389-393.

61. Farkas, K., et al., *New therapeutic targets in ulcerative colitis: the importance of ion transporters in the human colon.* Inflamm Bowel Dis, 2011. **17**(4): p. 884-98.

62. Heise, T., et al., *Impact of injection speed and volume on perceived pain during subcutaneous injections into the abdomen and thigh: a single-centre, randomized controlled trial.* Diabetes, Obesity and Metabolism, 2014. **16**(10): p. 971-976.

63. Berriaud, N., M. Milas, and M. Rinaudo, *Rheological study on mixtures of different molecular weight hyaluronates.* International journal of biological macromolecules, 1994. **16**(3): p. 137-42.
